# Versatile Antagonistic Activities of Soil-Borne *Bacillus* spp. and *Pseudomonas* spp. against *Phytophthora infestans* and Other Potato Pathogens

**DOI:** 10.3389/fmicb.2018.00143

**Published:** 2018-02-13

**Authors:** Simon Caulier, Annika Gillis, Gil Colau, Florent Licciardi, Maxime Liépin, Nicolas Desoignies, Pauline Modrie, Anne Legrève, Jacques Mahillon, Claude Bragard

**Affiliations:** ^1^Phytopathology-Applied Microbiology, Earth and Life Institute, Université Catholique de Louvain, Louvain-la-Neuve, Belgium; ^2^Laboratory of Food and Environmental Microbiology, Earth and Life Institute, Université Catholique de Louvain, Louvain-la-Neuve, Belgium

**Keywords:** *Bacillus* spp., bacilysin, bio-control, lipopeptides, potato diseases, *Phytophthora infestans*, *Pseudomonas* spp., siderophores

## Abstract

The world potato is facing major economic losses due to disease pressure and environmental concerns regarding pesticides use. This work aims at addressing these two issues by isolating indigenous bacteria that can be integrated into pest management strategies. More than 2,800 strains of *Bacillus*-like and *Pseudomonas*-like were isolated from several soils and substrates associated with potato agro-systems in Belgium. Screenings for antagonistic activities against the potato pathogens *Alternaria solani, Fusarium solani* (BCCM-MUCL 5492), *Pectobacterium carotovorum* (ATCC 15713), *Phytophthora infestans* (CRA-W10022) and *Rhizoctonia solani* (BCCM-MUCL 51929) were performed, allowing the selection of 52 *Bacillus* spp. and eight *Pseudomonas* spp. displaying growth inhibition of at least 50% under *in vitro* conditions, particularly against *P. infestans*. All 60 bacterial isolates were identified based on 16S rRNA gene sequencing and further characterized for the production of potential bio-active secondary metabolites. The antagonistic activities displayed by the selected strains indicated that versatile metabolites can be produced by the strains. For instance, the detection of genes involved bacilysin biosynthesis was correlated with the strong antagonism of *Bacillus pumilus* strains toward *P. infestans*, whereas the production of both bio-surfactants and siderophores might explain the high antagonistic activities against late blight. Greenhouse assays with potato plants were performed with the most effective strains (seven *Bacillus* spp. and four *Pseudomonas* spp.) in order to evaluate their *in vivo* antagonistic effect against *P. infestans*. Based on these results, four strains (*Bacillus amyloliquefaciens* 17A-B3, *Bacillus subtilis* 30B-B6, *Pseudomonas brenneri* 43R-P1 and *Pseudomonas protegens* 44R-P8) were retained for further evaluation of their protection index against *P. infestans* in a pilot field trial. Interestingly, *B. subtilis* 30B-B6 was shown to significantly decrease late blight severity throughout the crop season. Overall, this study showed that antagonistic indigenous soil bacteria can offer an alternative to the indiscriminate use of pesticide in potato agro-systems.

## Introduction

The increasing production of potato (*Solanum tuberosum* L.) is still facing important losses due to bacteria, fungi and fungus-like microorganisms, insects and viruses (Locke, 2002). Among these pathogens, the oomycete *Phytophthora infestans* (Mont.) de Bary, is responsible for causing late blight disease. The management of potato diseases, particularly late blight, is based on a massive use of chemical pesticides (i.e., fungicides such as mancozeb), causing tremendous costs, both direct and environmental (Cooke et al., 2011). The search for an environmental-friendlier pest management approach has led to study microbial agents with antagonistic capacities. Bacteria from both *Bacillus* and *Pseudomonas* genera are known to be appropriate candidates to be used in a bio-control approach due to their predominance in various environments, resilience and survival ability, but also for the number of bio-active molecules they are potentaially able to produce (Kloepper et al., 2004; Haas and Defago, 2005; Raaijmakers et al., 2010). Most of these bio-active compounds are secondary metabolites that exhibit direct and/or indirect antagonistic effects mediated through mechanisms such as antibiosis, competition, stimulation of plant growth and/or defenses (Emmert and Handelsman, 1999; Ongena and Jacques, 2008; Mavrodi et al., 2010).

Bacteria belonging to the genus *Bacillus*, particularly *Bacillus thuringiensis* strains with insecticidal properties, have been used in bio-control strategies since the mid-1930s (Bravo et al., 2011). Since then, the interest in *Bacillus* spp. has grown and numerous remarkable agricultural applications have been found. Some of them are currently exploited by the phytopharmaceutical industry which is commercializing some *Bacillus*-derived products: EcoGuard® by Novozymes (Gladsaxe, Hovedstaden, Denmark), Serenade® by Bayer (Leverkusen, North Rhine-Westphalia, Germany) or Kodiak® by Gustafson (Plano, TX, USA) as plant growth promoters and/or antagonists of phytopathogens with variable efficacy (Brannen and Kenney, 1997; Jacobsen et al., 2004; Kong et al., 2010; Lahlali et al., 2012). These claimed activities mostly rely on the enormous diversity of secondary metabolites produced by these bacteria, which can be divided into ribosomally synthesized peptides (e.g., bacteriocins), non-ribosomally synthesized peptides (e.g., lipopeptides, siderophores), polyketides (macrolides, polyenes) and volatile in-/organic compounds (hereinafter referred to as VIC and VOC). These compounds produced by *Bacillus* spp. are accompanied by various modes of action. Lipopeptides and VOCs, for instance, have direct antifungal activities as it is the case for the lipopeptide iturin A on *Rhizoctonia solani* (Yu et al., 2002) or volatiles pyrazine (2,5-dimethyl), benzothiazole and phenol-(4-chloro-3-methyl) against *Alternaria solani* and *Botrytis cinerea* on tomato plants (Gao et al., 2017). But other VOCs (2,3-butanediol, methyl jasmonate or methyl salicylate), as well as other lipopeptides (surfactin, fengycin) are also able to induce systemic resistance in plants through an ethylene-dependent pathway (Ryu et al., 2004; Ongena et al., 2007; Jourdan et al., 2009). Besides these activities, plant growth promotion was also reported using volatiles (2,3-butanediol, acetoin) on *Arabidopsis thaliana* (Ryu et al., 2003). Other compounds produced by *Bacillus* spp. like phytohormones (cytokinin, auxin) (Hussain and Hasnain, 2009; Lim and Kim, 2009) or siderophores (Yu et al., 2011) showed similar growth promoting effects on cucumber or red-pepper. In this last study, for example, the catecholic siderophore bacillibactin was proved to be involved in reducing the incidence of *Fusarium* wilt on pepper through induced systemic resistance, in addition to a direct bio-control effect.

*Pseudomonas* spp. are also known to produce a broad range of bio-active metabolites including lipopeptides, siderophores, polyketides, and volatile compounds with interesting activities against phytopathogens. It has been shown that these metabolites enable pseudomonads to directly compete with plant pathogens, promote plant growth or induce systemic plant resistance. The best example among these *Pseudomonas* spp. metabolites is the 2,4-diacetylphloroglucinol (DAPG). The antifungal activity of this polyketide has been extensively reviewed on damping-off, take-all or root rot diseases in various crops (Keel et al., 1992; Cronin et al., 1997; Huang et al., 2004; Ahmadzadeh and Sharifi Tehrani, 2009). Moreover, a clear link has been established between the disease-suppressiveness of some soils and the amount of DAPG producers (Weller et al., 2002) that include a large set of pseudomonads. But besides its direct antagonistic activity, DAPG is also involved in inducing systemic resistance through jasmonic acid and ethylene-dependent pathways (Iavicoli et al., 2003), highlighting its versatile mode of action. Lipopeptides are another example of the multiple antagonistic mechanisms used by pseudomonads. Some are known to lyse zoospores of *P. infestans* (de Souza et al., 2003; de Bruijn et al., 2007) and to induce resistance in tomato plants infected by this pathogen (Bakker et al., 2007; Tran et al., 2007). Pseudomonads siderophores (e.g. pseudobactin) are also able to induce systemic resistance in plants (Leeman et al., 1996; Meziane et al., 2005) and participate in direct competition with soil-borne plant pathogens for bio-available iron (Loper and Buyer, 1991). Siderophores produced by *Pseudomonas putida* or *Pseudomonas fluorescens*, for instance, are also able to promote plant growth and improve nutritional quality of major crops, such as rice, by enhancing the grain iron content (Sharma et al., 2013). Besides these soluble compounds, some VOCs produced by *Pseudomonas* spp. are also able to directly inhibit the development of *P. infestans* (de Vrieze et al., 2015) or *R. solani* (Kai et al., 2007).

In this study, we show the wealth of naturally occurring indigenous *Bacillus* spp. and *Pseudomonas* spp. strains associated with potato agro-systems that have the potential to be used at implementing biological control of potato diseases. For this purpose, a large collection of strains was isolated. Those displaying the highest *in vitro* antagonistic activity toward *P. infestans* were retained. An extensive characterization of these strains, including the detection of the genetic determinants of known antifungal metabolites, the *in vitro* assessment of lytic enzymes, the production of lipopeptides and siderophores and the *in vitro* activities against other potato pathogens, was assessed. The most interesting strains were tested on potato plants under greenhouse conditions. The implemented methodology allowed the selection of *Bacillus* spp. and *Pseudomonas* spp. bacteria with versatile mode of actions and very effective against *P. infestans*. Because *in vitro* antagonistic activities have been shown to be rarely reproducible in field conditions (Ravensberg, 2011; Glare et al., 2012), a pilot field trial was also conducted with the most promising strains selected in this study. The results pointed out the attractive potential of *B. subtilis* 30B-B6 to be used as a bio-control agent against late blight in field conditions.

## Materials and methods

### Sampling procedure and bacteria isolation

The sampling campaign was performed during the potato crop season from March to September 2012, mainly in loamy, shale-sandstone and sandy soils in Belgium. A total of 48 field soils from different locations and five soils from gardens were sampled from two soil horizons (A: 0–12 cm; B: 13–24 cm). Twenty-five other samples were also collected as follows: four from compost, eight from manure and 13 from potato plants divided in three parts (leaf, root and tuber) (Table S1). Plant samples were directly homogenized in 30 mL sterile Ringer's solution. Soil, compost and manure samples (25 g) were placed in blender bags with filters (pore size 330 μm; VWR) and mixed with 30 mL of sterile Ringer's solution in a Stomacher® machine (Seward) for 10 s. Samples were then collected through the blender bag filter and decanted overnight at 4°C. Each sample was then divided in aliquots supplemented with glycerol (3:1 vol/vol) and cryopreserved at −80°C for further bacterial selective isolation. *Bacillus*-like strains were isolated as follows: 1 mL of each sample was subjected to heat treatment (75°C, 20 min), 10-fold diluted with sterile Ringer's solution, plated on Lysogeny Broth (LB; also known as Luria-Bertani) (NaCl, 5 g L^−1^; yeast extract, 5 g L^−1^; tryptone, 10 g L^−1^) solidified with 1.4% (wt/vol) agar and incubated at 30°C for 24 h. *Pseudomonas*-like selective isolation relied on growth (30°C, 24 h) on Cephalothin-Sodium Fusidate-Cetrimide (CFC) agar medium (Biokar Diagnostics®), combined with oxidase test for presumptive *Pseudomonas* strains. After selective isolation, nine strains of each genus were retained for each sample. The final collection of more than 2,800 isolates was then conserved in LB-glycerol (70:30 vol/vol) at −80°C.

### Microorganisms, culture conditions and potato plant varieties used in this study

Five potato pathogens were used in this work (Table 1): *A. solani* and *P. infestans* were cultivated on V8-agar (CaCO_3_, 1 g L^−1^; agar, 15 g L^−1^; vegetable juice V8, 200 mL L^−1^) in the dark, at room temperature and 18°C, respectively. *F. solani* and *R. solani* were cultivated on potato dextrose-agar (PDA, Oxoid) in the dark at 22°and 20°C, respectively. *Pectobacterium carotovorum* was grown overnight at 28°C on LB-agar. Bacteria belonging to the *Bacillus and Pseudomonas* genera were routinely grown on LB-agar (*Bacillus* spp. and *Pseudomonas* spp.) or PDA (*Pseudomonas* spp.). Certified tubers of *S. tuberosum* “Bintje” and “Challenger” varieties from CONDIPLANT® (Gembloux, Belgium) were used in greenhouse experiments and field trials. For greenhouse experiments, tubers were grown in pots containing twice-sterilized compost, daily watered and, maintained under controlled conditions with a photoperiod of 16:8 h at 25/15°C (day/night). For field trials, 10 acres of soil were supplemented with a mix of Nitrogen/Phosphorus/Potassium (NPK: 12-9-22, 1000 Kg ha^−1^) and NH_4_ (27%, 300 kg ha^−1^). Prior to tuber plantation, an herbicide treatment was performed (2 Kg ha^−1^ Artist®, 1 L ha^−^^1^ Linuron®, 2 L ha^−1^ Challenge®; water 250 L ha^−1^). No chemical weed control was performed before harvest and weather conditions were daily registered throughout the growing season.

**Table 1:** Potato pathogens used in this study.

**Pathogens**	**Remarks**	**Strain source**
**BACTERIA**		
*Pectobacterium carotovorum*	Reference strain	ATCC 15713^a^
**CHROMISTA**		
*Phytophthora infestans*	Belgian field isolate, A2 mating type, race 1.3.4.5.7.10.11	CRA-W10022^b^
**FUNGI**		
*Alternaria solani*	Belgian field isolate	Not defined
*Fusarium solani*	Belgian field isolate	BCCM-MUCL 5492^c^
*Rhizoctonia solani*	Belgian field isolate	BCCM-MUCL 51929^c^

a *ATCC, American Type Culture Collection, Manassas, VA, United States*.

b *CRA-W, Walloon Agronomical Research Center, Libramont, Belgium*.

c *BBCM-MUCL, Microbial Collection of the Université catholique de Louvain, Louvain-la-Neuve, Belgium*.

### Direct antagonism assays

**Figure 1:**
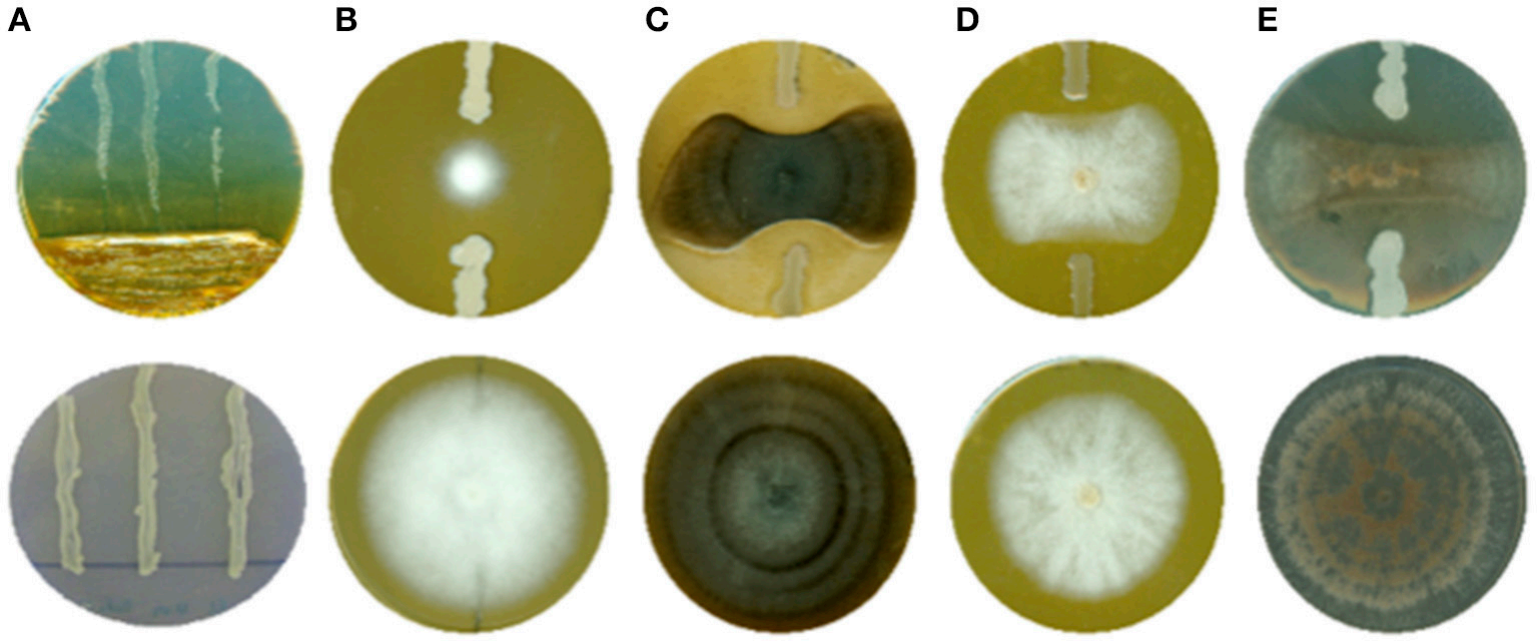
*In vitro* confrontation (up) and negative control (down) of **(A)** *Pectobacterium carotovorum*, **(B)** *Phytophthora infestans*, **(C)** *Alternaria solani*, **(D)** *Fusarium solani*, and **(E)** *Rhizoctonia solani*. The percentage of growth inhibition was calculated through image analysis by comparing the area covered by tested pathogen with negative control. Each test was done three times, in triplicate.

Direct antagonism activities were assessed through confrontation on solid media in Petri dishes. For this purpose, bacterial cultures were prepared in 10 mL LB medium inoculated by a single colony and incubated 24 h at 30°C and 120 rpm. Antagonistic activities against *P. carotovorum* were evaluated by streaking the bacterial culture of interest in one third of the Petri dish containing LB-agar. Then, an overnight (O/N) broth culture of *P. carotovorum* (28°C, 120 rpm) was streaked three times perpendicularly to the bacterial inoculum (Figure 1A). LB medium was used as negative control. Petri dishes were scanned (HP Scanjet G4010) after 48 h of incubation. To evaluate the potential antagonistic activity against the other pathogens, two streaks of two cm of the bacterial culture of interest were made at each side of a piece of agar (Ø 5 mm) covered with the pathogen mycelium aged of 7–10 days (Figures 1B–E). As negative control, LB medium was streaked at each side of the piece of agar covered with the pathogen mycelium. Petri dishes were scanned (HP Scanjet G4010) after 7–10 days of incubation. Image analysis (Image J® software) was used to quantitatively determine the Growth Inhibition Percentage (GIP) by comparing the surface covered by the pathogen in the presence of bacterial isolate and the negative controls. Antagonistic activity was categorized in four different classes: (i) GIP < 30%, (ii) GIP ≥ 30%, (iii) GIP ≥ 50% and GIP ≥ 70% (Figure S1). Each test was repeated twice with three technical replicas and statistically treated. At the end of those tests, the 60 most active strains (GIP ≥ 50%) were selected for further characterization.

### Bacterial isolates identification

Bacterial identification was performed through 16S rRNA gene sequencing. For this, bacterial isolates were grown overnight on LB-agar and one single colony was picked-up for “colony-PCR” using primers pair 27F (AGAGTTTGATCCTGGCTCAG) and 1492F (GGTTACCTTGTTACGACTT). PCR reactions were performed using the GoTaq® G2 Flexi DNA Polymerase (Promega) with colorless buffer following the manufacturer's recommendations. Thermal cycling parameters were as follows: a denaturation step at 95°C for 5 min followed by 30 cycles at 95°C for 1 min, 55°C for 30 s and 72°C for 90 s. Finally, an elongation step at 72°C for 10 min. PCR amplicons were verified by gel electrophoresis, purified using the GenElute PCR cleanup kit (Sigma) and sequenced in both orientations at Macrogen Europe (Amsterdam, The Netherlands). For each bacterial isolate, nucleotide sequences were trimmed, aligned and compared with the BLASTn search available in GenBank database. Sequences were deposited in the NCBI database under GenBank accession numbers MF062580 to MF062639. Phylogenetic relationships based on partial 16S rRNA gene sequences were determined with MEGA 6.0 software (Tamura et al., 2013) using maximum likelihood (ML) method with the General Time-Reversible plus gamma model of nucleotide substitution and bootstrap values of 1,000 iterations.

### PCR screening for genes related to known antagonistic metabolites and virulence factors

Total DNA from bacterial isolates were extracted by using the Wizard® Genomic DNA Purification Kit (Promega) following manufacturer's instructions. DNA extractions were PCR-screened for the presence of the genetic determinants of previously reported antagonistic metabolites and virulence factors (Table S2). All PCR reactions were performed using the One *Taq*® Quick-Load 2X Master Mix with standard buffer (New England BioLabs) following the manufacturer's recommendations. Thermal cycling parameters were 5 min at 94°C, then 35 cycles of 1 min at 94°C, 1 min at the annealing temperature, followed by 1 min/kb of the PCR amplicon (Table S2) at 68°C, and finally, 10 min at 68°C. Reference strains used as positive controls for each PCR are indicated in Table S2. PCR amplicons were analyzed by agarose gel electrophoresis.

### Production of enzymes, siderophores and bio-surfactants by the bacterial isolates

The production of enzymes (Ariffin et al., 2006; Youcef-Ali et al., 2014), siderophores (Neilands, 1981) and bio-surfactants (Siegmund and Wagner, 1991; Youssef et al., 2004) were assessed for each selected bacterial isolate. Briefly, the production of proteolytic, cellulolytic and chitinolytic enzymes, along with the siderophore production, were respectively assessed throughout halo formation on specific solid media: Skimmed Milk (SM)-agar [Skimmed milk, 28g L^−^^1^; casein hydrolysate, 5 g L^−1^; yeast extract, 2 g L^−1^; dextrose, 1 g L^−1^; agar, 15 g L^−1^ at pH 7.0 ± 0.2]; Carboxy-methyl Cellulose (CMC)-agar [KH_2_PO_4_, 1 g L^−1^; MgSO_4_.7H_2_O, 0.5 g L^−1^; NaCl, 0.5 g L^−1^; FeSO_4_.7H_2_O, 0.01 g L^−1^; MnSO_4_.H_2_O, 0.01 g L^−1^; NH_4_NO_3_, 0.3 g L^−1^; CMC, 10 g L^−^^1^; agar, 12 g L^−1^ at pH 7.0 ± 0.2]; Colloidal Chitin (CC)-agar [NH_4_H_2_PO_4_, 1 g L^−1^; KCl, 0.2 g L^−1^; MgSO_4_.7H_2_O, 0.2 g L^−1^; CC, 10 g L^−1^; agar, 20 g L^−1^ at pH 6.0 ± 0.2]; and modified Chrome Azurol S (CAS)-agar [10 mL of FeCl_3_.6H_2_O, 27 mg/100 mL of HCl 10 mM; 50 mL of CAS, 1.2 g L^−1^; 40 mL of HDTMA, 1.82 g L^−^^1^; 900 mL of LB-agar for *Bacillus* spp. and King Broth agar for *Pseudomonas* spp. at pH 6.8 ± 0.2]. A 10 μL drop of a bacterial culture in LB (24 h, 30°C, 120 rpm) was spotted on the above-mentioned media and activity was observed after an incubation of 24 h at 30°C (proteolytic enzymes), 120 h at 37°C (cellulolytic enzymes), 120 h at 30°C (chitinolytic enzymes and siderophores). Each test was repeated twice with three technical replicas. Bio-surfactant production was assessed via a “drop collapse test” based on amphiphilic properties of compounds like lipopeptides. Twenty-five μL drops of filtered supernatant (0.22 μm Minisart®, Sartorius Stedim) from a 48 h bacterial culture (*Bacillus* spp.: LB, 30°C, 120 rpm; *Pseudomonas* spp.: KB, 25°C, 120 rpm) were spotted on a hydrophobic surface (Petri dish Sarstedt®). Drops of sterile media (LB; KB) were used as negative control. After 5 min of incubation at room temperature, plates were scanned (HP Scanjet G4010) and the area covered by the drop was measured with an image analysis software (Image J®). The percentage of “spreading” was then calculated by comparison with the negative control and normalized with the optical density at 600 nm (OD_600nm_) of the initial culture (Multiskan FC, Thermo Scientific). Each test was performed four times with three technical replicas. *Bacillus amyloliquefaciens* S499, *B. subtilis* GA1 and *Pseudomonas aeruginosa* PAO1 were used as positive controls, since they are known as bio-surfactant producers (de Souza et al., 2003; Touré et al., 2004; Arguelles-Arias et al., 2009; Nihorimbere et al., 2012). *Pseudomonas* spp. bio-surfactant production was also assessed via a culture of 5 days on Siegmund and Wagner (SW) medium [glycerol, 20 g L^−1^; KH_2_PO_4_, 0.7 g L^−1^; Na_2_HPO_4_, 0.9 g L^−1^; NaNO_3_, 2 g L^−1^; MgSO_4_.7H_2_O, 0.4 g L^−1^; CaCl_2_.2H_2_O, 0.1 g L^−1^; 2 mL L^−1^ of trace elements containing FeSO_4_.7H_2_O, 2 g L^−1^; MnSO_4_.H_2_O, 1.5 g L^−1^; (NH_4_)_6_Mo_7_O_24_.4H_2_O, 0.6 g L^−1^; at pH 6.7 ± 0.2] supplemented with cetyltrimethylammoniumbromide (CTAB)-methylene blue agar [CTAB-methylene, 0.2 g L^−1^; methylene blue, 0.005 g L^−1^; agar, 15 g L^−1^] where bio-surfactant producers are able to form a dark blue halo around the colony (Siegmund and Wagner, 1991).

### Bio-control assays against late blight in greenhouse

Greenhouse experiments were performed in order to evaluate the antagonistic effect of selected bacteria against potato late blight. Assays were conducted with seven *Bacillus* spp. and four *Pseudomonas* spp. showing high *in vitro* antagonistic activities (GIP ≥ 70%) against *P. infestans*. Potato plants were obtained by planting potato tubers from sensitive “Bintje” variety and growing them for a month in a G2 greenhouse facility with cycles at 25/15°C (day/night) with 70% relative humidity (RH) and 16:8 photoperiod. Potato plants were arranged every 30 centimeters and were daily irrigated. Bacteria were grown in LB for 48 h at 30°C and 120 rpm. Bacterial cultures adjusted to an OD_600nm_ of 1 with LB were sprayed on potato leaves using 6 plants by treatment (2 mL/leaf and 3 leaves/potato plant) 4 h before pathogen inoculation. Sterile LB was used as negative control. Prior to the pathogen inoculation, *P. infestans* strain CRA-W10022 pathogenicity was restored *in vitro* on potato leaves as described by Clinckemaillie et al. (2016). Suspensions of *P. infestans* (ca. 15,000 sporangia mL^−1^) were applied through pulverization (2 mL/leaf). Plants were placed in a humid chamber for 24 h at 90% RH to enable infection and then moved back to the G2 greenhouse. Quantitative scoring of the symptoms was done daily as described by Clinckemaillie et al. (2016) and the disease progression was followed for 7–10 days after inoculation. At the end of the experiment, the area under the disease progression curve (AUDPC) was calculated for each replica (Campbell and Madden, 1990) in order to determine a normalized protection index (PI) calculated as follows:

$$\begin{array}{l} \text{PI}=\left[\left(1-\frac{\text{AUDPC treatment}}{\text{AUDPC control}}\right)*100\right] \end{array}$$

The greenhouse assays were repeated four times using 18 technical replicas (6 plants, 3 leaves/potato plant) each and were statistically treated.

### Pilot field trial against late blight

A pilot field assay aiming at assessing selected bacteria effectiveness as bio-control agents of *P. infestans* was conducted at the Alphonse de Marbaix Research Center in Louvain-la-Neuve (Belgium) from April to September 2016. A randomized complete block assay was designed with 7 treatments (4 replicas/treatment, 4 lines/replica, 48 plants/replica) and interstices of sensitive “Bintje” potato variety distributed on a 10,000 m^2^ field (15 m^2^/replica). ‘Bintje’ potato plants were used as reservoir to improve natural *P. infestans* infection in field. Bacterial strains of *Bacillus* spp. and *Pseudomonas* spp. were selected on the basis of *in vitro* and *in vivo* assays performed against *P. infestans*. Treatments applied were divided as follows: (i) plants treated with antagonistic strain *B. amyloliquefaciens* 17A-B3, (ii) plants treated with antagonistic strain *B. subtilis* 30B-B6, (iii) plants treated with antagonistic strain *Pseudomonas brenneri* 43R-P1, (iv) plants treated with antagonistic strain *Pseudomonas protegens* 44R-P8, (v) plants treated with chemical fungicide Revus® (250 g L^−1^ mandipropamid; 300 L ha^−1^), (vi) plants treated with Cuprex® (300 L ha^−1^), an authorized fungicide for organic production and (vii) untreated potato plants from late blight semi-resistant “Challenger” variety. Bacterial cultures suspensions were produced as described for greenhouse experiments and 2.5 L/replica were foliar-sprayed once a week from 8th June to 17th August 2016 (12 applications). Fungicides, supplemented with Trend 90® (1 mL L^−1^), were applied six times in the potato crop growing season following Belgian warning prediction system (CARAH, PCA). Late blight disease progression was monitored as described for greenhouse assays, three times a week throughout the growing potato crop season, with a quantitative scoring of all leaves of eight plants/replica. AUDPC and normalized PI were calculated as described above.

### Statistical analysis

Data from antagonistic activity assays *in vitro*, greenhouse assays and pilot field trial were statistically treated using the one-way analysis of variance (ANOVA) using open source software R® (Ihaka and Gentleman, 1996). The Tukey HSD multiple-comparison test was used for comparison of the means, with confidence interval specified through *p*-value. The Pearson Correlation Index (PCI) was calculated with corrplot package in R® (Wei and Simko, 2016).

## Results

### Isolation and *in vitro* screening of bacteria displaying antagonistic activities

A collection of 2,826 bacterial strains was isolated from a total of 157 collected samples (Table S1). Based on their *in vitro* antagonistic activities toward at least one of the five pathogens tested (*A. solani, F. solani, R. solani, P. carotovorum, P. infestans*) with a GIP ≥ 50%, 52 *Bacillus* spp. and eight *Pseudomonas* spp. strains were selected for further characterization (Tables 2, 3). For this subset of 60 strains, 12 *Bacillus* spp. strains were shown to be active against *F. solani* (GIP ≥ 50%), whereas no *Pseudomonas* spp. showed a strong activity against this fungus. It is worth to note that due to the rhizoidal growth displayed by nine of the active *Bacillus* strains, their activity against *F. solani* might also be due to competition for surface colonization (Figure S2). Concerning the antagonism against late blight (*P. infestans*), 18 *Bacillus* spp. and seven *Pseudomonas* spp. strains displayed a strong inhibitory activity (GIP ≥ 70%). Also, 25 *Bacillus* spp. strains showed a good inhibition activity against *A. solani* (GIP ≥ 50%) and four against *P. carotovorum*, while none of the *Pseudomonas* strains were active against these two microbes. The assays against *R. solani* also revealed effective strains of *B. amyloliquefaciens* (one), *B. pumilus* (three) and *P. putida* (one). Taken together, a strong *in vitro* antagonistic activity (GIP ≥ 70%) against at least one of the pathogens tested was observed for 26 *Bacillus* spp. and five *Pseudomonas* spp. strains.

**Table 2:**
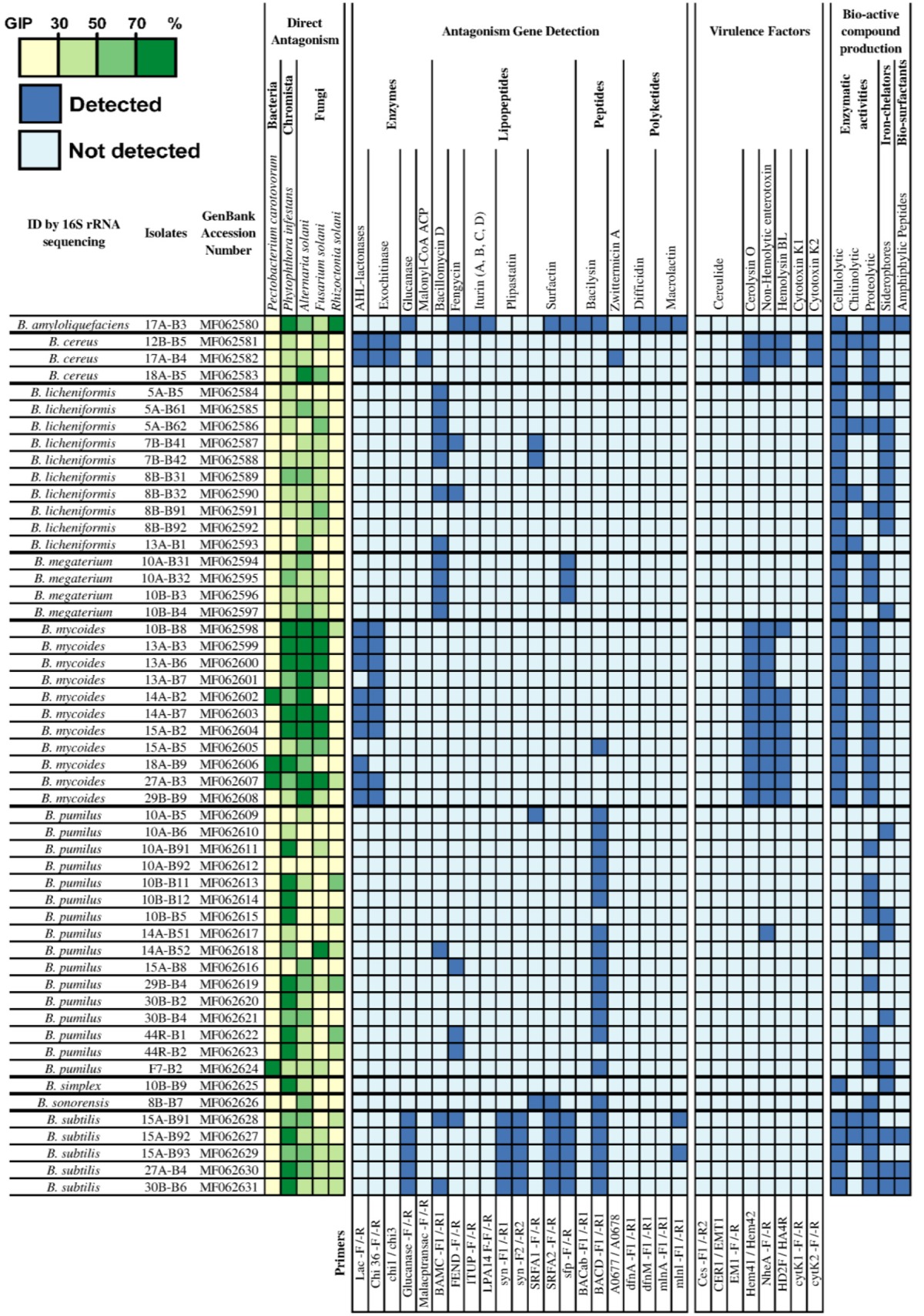
*Bacillus* spp. strains isolated in this study and selected for their statistically relevant *in vitro* antagonistic activities, evaluated through observed growth inhibition percentage (GIP), against *P. carotovorum, P. infestans, A. solani, F. solani*, and *R. solani*.

*Genbank accession number, isolate code, 16S identification, antagonism exhibited toward pathogens tested (scaled as described in color legend at the top of the table), PCR detection of related genes to known bio-active metabolites and virulence factors, and bio-active compound production (enzymes, siderophores, bio-surfactants) are indicated*.

**Table 3:**
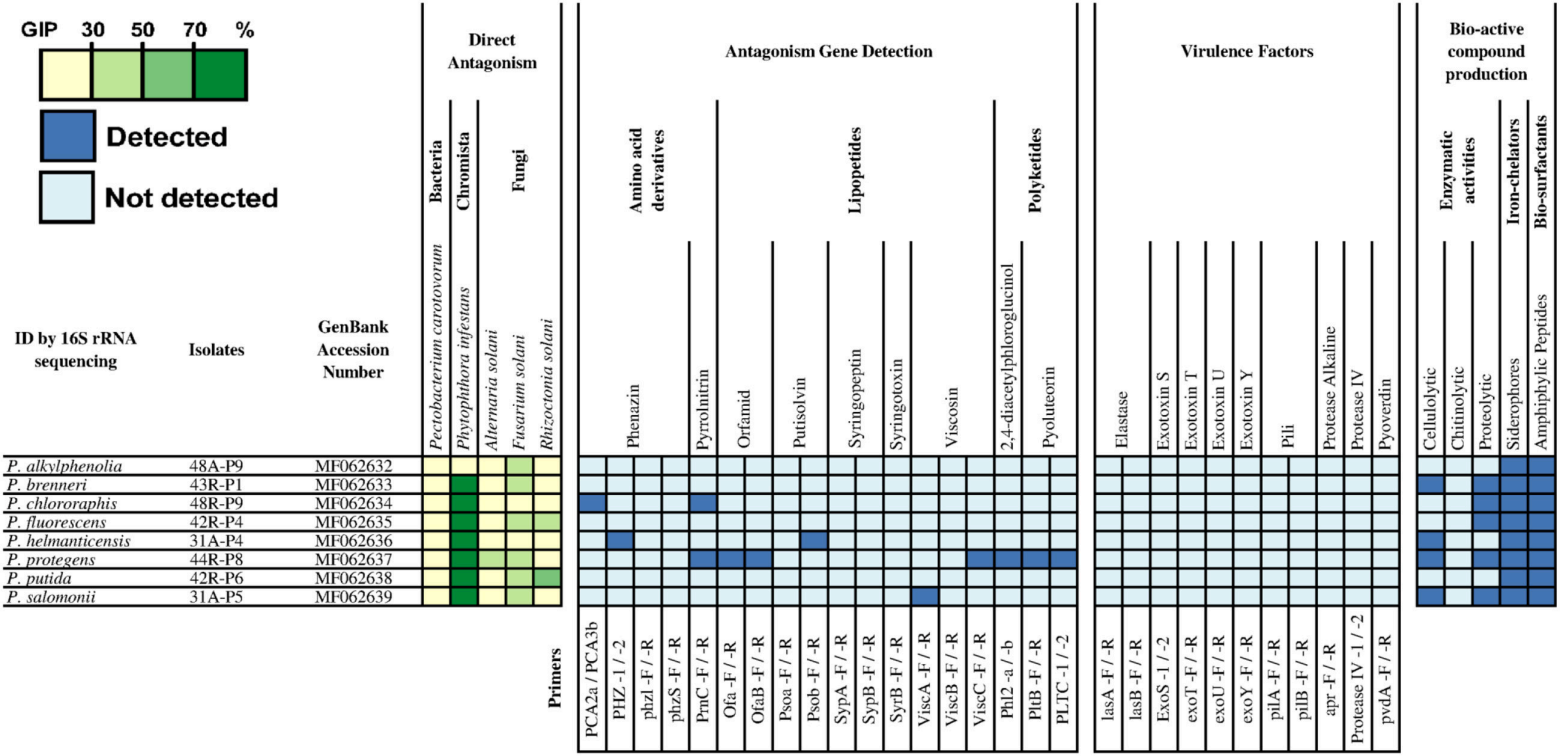
*Pseudomonas* spp. strains isolated in this study and selected for their statistically relevant antagonistic activities, evaluated through observed growth inhibition percentage (GIP), against *P. carotovorum, P. infestans, A. solani, F. solani*, and *R. solani*.

*Genbank accession number, isolate code, 16S identification, antagonism exhibited toward pathogens tested (scaled as described in color legend at the top of the table), PCR detection of related genes to known bio-active metabolites and virulence factors, and bio-active compound production (enzymes, siderophores, bio-surfactants) are indicated*.

### Identification of the antagonistic bacteria

BLAST and phylogenetic analyses based on 16S rRNA gene sequences revealed that the majority of the selected *Bacillus* spp. strains belong to the groups of *B. subtilis* (i.e., *B. amyloliquefaciens, B. licheniformis*, and *B. sonorensis*) and *B. cereus sensu lato (s.l.)* (i.e., *B. cereus* and *B. mycoides*) (Table 2, Figure S3). It is worth noting that the *B. mycoides* strains were further identified through their characteristic rhizoid growth on LB plates. Strains belonging to the *B. pumilus* complex, along with *B. megaterium* and *B. simplex*, were also identified among the selected strains as displaying remarkable antagonistic activities against the pathogens tested. It is interesting to note that the strains that showed strong competitive abilities, which are not necessary antagonistic activities, are all *B. mycoides* except for one *B. cereus* strain. Selected isolates from the genus *Pseudomonas* were identified through 16S rRNA gene sequences as *P. alkylphenolia, P. brenneri, P. chlororaphis, P. fluorescens, P. helmanticensis, P. protegens, P. putida*, and *P. salomonii* (Table 3, Figure S4).

### PCR screening for known antagonistic metabolites

Using a PCR approach, the genetic determinants of potential antagonistic molecules screened for the collection of *Bacillus* strains were (Table S2): β-1,3-glucanase, AHL-lactonases, bacilysin, bacillomycin D, chitinases, difficidin, fengycin, iturin, macrolactin, plipastatin, surfactin and Zwittermicin A. As shown in Table 2, it seems evident that each species of *Bacillus* spp. present a specific pattern of detection associated with potential antagonistic metabolites. Indeed, *B. mycoides* strains were mostly positive to AHL-lactonases (PCI: 0.77), and exochitinases (PCI: 0.77), *B. licheniformis* strains to bacillomycin D (PCI: 0.47), *B. megaterium* strains to bacillomycin D (PCI: 0.48) and surfactin (PCI: 0.33), and *B. pumilus* strains to bacilysin (PCI: 0.61). Spectra of potential antagonistic metabolites were broader for *B. subtilis* strains (15A-B91, 15A-B92, 15A-B93, 27A-B4, 30B-B6) and even reached seven bio-active compounds for *B. amyloliquefaciens* strain (17A-B3). Bacilysin-related genes were the most commonly detected, in particular in all the strains displaying a strong inhibitory activity against *P. infestans* (GIP ≥ 70 %). These strains belong to the *B. amyloliquefaciens, B. pumilus*, and *B. subtilis* species (10 strains). Remarkably, five of these strains displayed positive PCR results only for bacilysin, which might indicate that this molecule was responsible for the strong antagonism observed toward *P. infestans*.

The genetic determinants associated with the production of phenazin, pyrrolnitrin, orfamid, syringopeptin, viscosin, 2,4-diacetylphloroglucinol, and pyoluteorin were detected by PCR in four strains of *Pseudomonas* spp. (Table 3) suggesting their potential implication in the *in vitro* antagonistic activities against the pathogens. In particular, *P. protegens* 44R-P8, which was positive for 5/10 molecules tested by PCR, showed an important *in vitro* antagonistic potential. Interestingly, although the *P. brenneri* 43R-P1 strain was negative to all tested genes, it displayed a strong antagonism toward *P. infestans* (GIP ≥ 70%; Table 3).

### PCR screening for virulence factors

The detection by PCR of the genetic determinants related to potential virulence factors of *B. cereus s.l*. group and *Pseudomonas aeruginosa* species was performed on all strains of the collection since some members of these groups may be potential human pathogens. Among the collection of *Bacillus* spp., the screening indicated that strains belonging to *B. mycoides, B. cereus* and one strain of *B. pumilus* were positive for at least one gene coding for a potential virulence factor, mainly the non-hemolytic enterotoxin (Nhe), Hemolysin BL (HBL) or Cerolysin O (CerO) (Table 2). However, none of the *Bacillus* spp. were positive to the genetic determinants coding for the lethal toxins cereulide and cytotoxin K1 (CytK1), both associated with severe foodborne toxi-infections in humans, including fatalities (Lund et al., 2000; Naranjo et al., 2011). Among the selected *Pseudomonas* spp. strains, none were positive to any tested genes related to the potential virulence factors (Finnan et al., 2004; Lanotte et al., 2004; Khalifa et al., 2011; Holban et al., 2013).

### Evaluation of the production of enzymes, siderophores, and bio-surfactants

The assays performed to evaluate enzymatic activities (i.e., proteolytic, cellulolytic and chitinolytic) of the selected strains indicated that the majority of the *Bacillus* spp. and *Pseudomonas* spp. were able to produce proteolytic and cellulolytic enzymes. On the contrary, very few *Bacillus* spp. and no *Pseudomonas* strains were able to display chitinolytic activity on colloidal chitin medium (Tables 2, 3). No relation was found between any specific bacterial species, their enzymatic activities and their antagonistic behavior (data not shown). Regarding the siderophore production, while all *Pseudomonas* spp. strains produced siderophores under the condition tested, only 19 *Bacillus* spp. strains did, mostly strains of *B. licheniformis, B. pumilus*, and *B. subtilis* (Tables 2, 3). Drop collapse tests also showed that all the *Pseudomonas* spp. strains were able to produce bio-surfactants (Table 3), while only four strains of the *B. subtilis* group did (Table 2).

### Greenhouse assays against late blight

**Figure 2:**
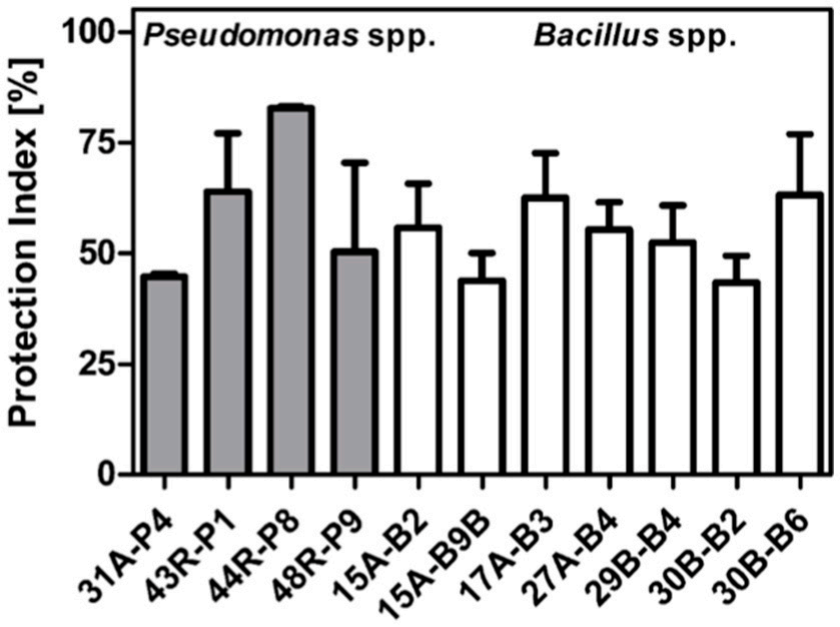
Mean of normalized protection index (PI) against late blight observed after foliar spray on sensitive “Bintje” variety of potato plants with *Pseudomonas* spp. (gray) or *Bacillus* spp. strains (white), and standard deviation based on four greenhouse assays.

**Figure 3:**
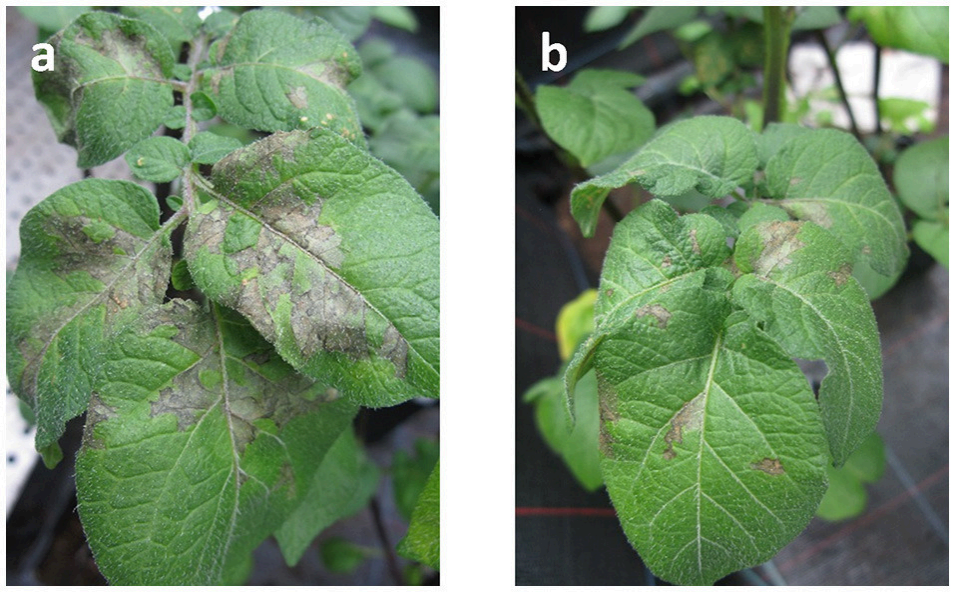
Late blight progression observed in greenhouse assay after foliar spray of *P. infestants* (15,000 sporangia mL-1) on: **(a)** sensitive “Bintje” variety of potato plant and **(b)** potato plant previously treated with *P. protegens* 44R-P8 suspension.

*In vivo* assays conducted under controlled greenhouse conditions were designed and tested beforehand to ensure a well-established and reliable pathosystem allowing the pathogen to grow under good conditions and perform a complete infection of inoculated leaves under 8 days. Results from statistical analysis (ANOVA) performed on 72 replicas for each tested strain, distributed among four assays, confirmed the strong antagonistic activities of seven *Bacillus* spp. and four *Pseudomonas* spp. against *P. infestans* on the sensitive potato variety “Bintje.” As shown in Figures 2, 3, three *Bacillus* spp. (30B-B6, PI: 67%; 15A-B2, PI: 65%; 17A-B3, PI: 62%) and two *Pseudomonas* spp. (44R-P8, PI: 83%; 43R-P1, PI: 64%) gave significant protection (PI > 60 %) against *P. infestans* after disease developmental level normalization.

### Pilot field trial against late blight

**Figure 4:**
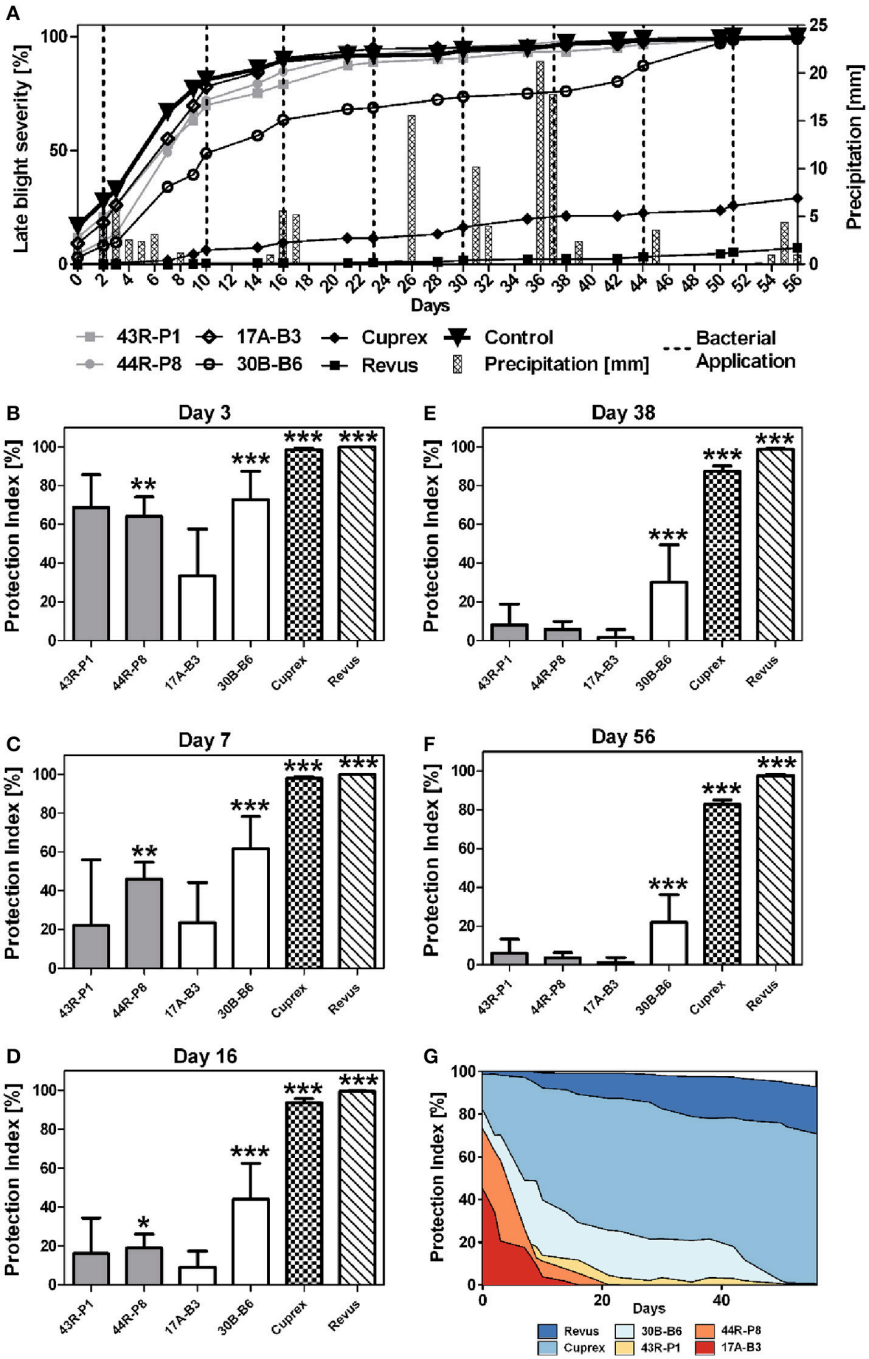
**(A)** Mean of late blight severity (percentage of symptomatic leaf area) observed in field trial on “Challenger” variety potato plants: non-treated (▾) and treated with: *Pseudomonas* spp. strains 43R-P1 (■) and 44R-P8 (•), *Bacillus* spp. strains 17A-B3 (♢) and 30B-B6 (°), fungicides Cuprex® (♦) and Revus® (■). **(B–F)** Mean of normalized protection index (PI) against late blight in field assay on semi-resistant “Challenger” variety of potato plants observed after foliar spray with *Pseudomonas* spp. (gray), *Bacillus* spp. (white), fungicides Cuprex® and Revus® on days 3–56. **(G)** Mean of normalized PI evolution. Variance analysis (ANOVA) showed that at the end of pilot field trial, normalized PI is statistically significant for *Bacillus* 30B-B6 and fungicide treatments (^*^p-value < 0.1; ^**^p-value < 0.01; ^***^p-value < 0.01).

**Figure 5:**
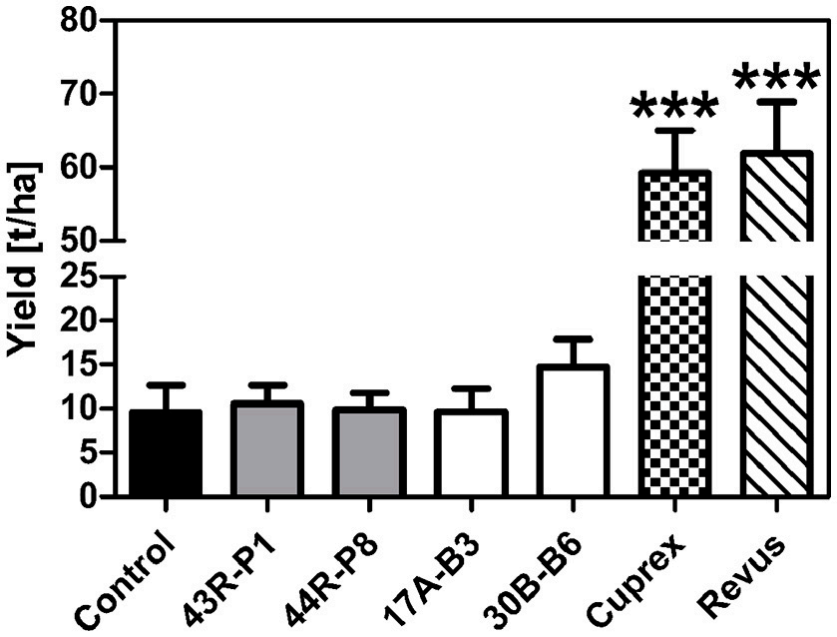
Mean yield obtained at the end of the pilot field trial with the “Challenger” variety of potato plants non-treated (control) and treated with: *Pseudomonas* spp. strains 43R-P1 and 44R-P8, *Bacillus* spp. strains 17A-B3 and 30B-B6, fungicides Cuprex® and Revus®. Variance analysis (ANOVA) showed that yield enhancement is statistically significant for fungicide treatments (^***^p-value < 0.001).

Based on greenhouse assays, *B. amyloliquefaciens* 17A-B3 and *B. subtilis* 30B-B6, along with *P. brenneri* 43R-P1 and *P. protegens* 44R-P8 were evaluated in a pilot field trial to assess their PI against *P. infestans*. Environmental conditions during field trial (April, 20 to September, 22, 2016) were appropriate for late blight infection and development. Potato plants of semi-resistant “Challenger” variety reached 99.8% of late blight infection at the end of evaluations, while the sensitive “Bintje” potato plants presented already 100% of late blight infection from the sixteenth day. During the assay, significant rainy events occurred and matched with temperatures favorable for the growth and transmission of *P. infestans* in the field (Figure S5). Disease progression was followed through quantitative evaluations from June, 27 (day 0) to August, 22 (day 56) (Figure 4A), allowing calculation of AUDPC and determination of a normalized PI (Figures 4B–G). The protection conferred by bacterial treatments was less effective than fungicide treatments at any time in the assay. As expected, the fungicide Cuprex and Revus treatments allowed a disease severity reduction of 83 and 98%, respectively. However, the bacterial treatments with *P. protegens* 44R-P8 strain provided a significant protection for 16 days after the first signs of symptoms (Figures 4B–D) and up to 45 days with *B. subtilis* 30B-B6 (Figures 4B–F). Moreover, treatment with 30B-B6 strain allowed shifts in time of 5 days to reach 25 and 50% of infection compared to controls. Interestingly, protection conferred by the treatment with *B. subtilis* 30B-B6 stabilized around 20% for nearly 30 days from day 14 to day 42 (Figure 4G). At the end of the trial, the treatment with *B. subtilis* 30B-B6 allowed a statistically significant reduction of late blight severity (PI: 22%; *p*-value < 0.001). Remarkably, monitoring of late blight progress showed that each application of *B. subtilis* 30B-B6 allowed a decrease in the disease development rate until an infection rate of 90% (Figure 4A), even in rainy conditions. The other bacterial treatments did not allow a significant symptoms reduction up to the end of the trial (Figure 4F). Tubers were harvested on September, 22 (day 85) when the yield data (t ha^−1^) were monitored. They showed a minor increase (not statistically significant) in yield of potato treated with *B. subtilis* 30B-B6 compared to the other bacterial treatments and control potato plants (Figure 5).

## Discussion

In this study, we highlighted the fact that, in soil and/or on plants, there is a wealth of *Bacillus* and *Pseudomonas* bacteria present and displaying versatile antagonistic activities against potato pathogens. Starting from more than 2,800 environmental isolates, a core collection of 52 *Bacillus* spp. and eight *Pseudomonas* spp. strains was selected for further analyses based on their significant *in vitro* antagonistic activities against important potato pathogens. Sequencing of 16S rRNA gene of selected bacteria revealed that the *Pseudomonas* strains belong to the *P. fluorescens* group (3), to the *P. putida* group (1), to the *P. chlororaphis* group (1) and to undefined groups, while antagonistic *Bacillus* strains belong mainly to the *B. subtilis* and *B. cereus* groups (Jensen et al., 2003; Wang et al., 2007). Antagonistic activities of *P. fluorescens* have already been described (Weller et al., 2002; de Souza et al., 2003; Haas and Defago, 2005) and were associated with specific metabolites such as DAPG. Nevertheless, our PCR screening for known antagonistic metabolites revealed only one strain (44R-P8) positive for the detection of genetic determinants involved in DAPG biosynthesis. This is an encouraging perspective for novel bio-active compound discovery because the other three *Pseudomonas* spp. strains (43R-P1, 42R-P4, 42R-P6) were highly effective against *P. infestans* and negative to all the other target genes.

*Bacillus* spp. also possess numerous interesting properties for industries and agriculture. As a matter of fact, *Bacillus* spp. based products are present on the market as bioinsecticides since early fifties with strains of *B. thuringiensis* that represent around 2% of the total insecticidal market (Bravo et al., 2011). Various products based on *Bacillus* spp. with other interesting properties have also been commercialized. These bacteria based products are claimed to be active through various mode of action due to production of a large set of bio-active metabolites. Nevertheless, the harmlessness of the use of biological control agents (BCA) is not always taken into account. Therefore, a screening for potential virulence factors was performed on our core collection. This screening revealed that no *Pseudomonas* spp. were positive to any of the potential virulence factor tested. With the exception of one *B. pumilus*, only strains belonging to *B. cereus s.l*. group (*B. cereus* and *B. mycoides*) were tested positive to some potential virulence factors (i.e., CerO, Nhe and HBL) but none for the lethal toxin cereulide or the cytotoxin K1 enterotoxin. Moreover, the well-known biopesticides *B. thuringiensis* serovar *kurstaki* HD-1 (Day et al., 2014) and *B. cereus* UW85 (Handelsman et al., 1990; Lozano et al., 2016) also harbor the genetic determinants coding for these potential virulence factors (data not shown) and they are used worldwide.

In this work, an extensive PCR screening performed on our core collection indicated numerous bio-active metabolites potentially produced by strains displaying remarkable antagonistic activities. It is worth noting that the mechanisms of action of many of these antagonistic molecules remain poorly understood and are associated with generic concepts such as plant growth and defense promotion, or antibiosis. The diversity in the metabolic arsenal of strains such as *B. amyloliquefaciens* (17A-B3), *B. subtilis* (15A-B91, 15A-B92, 15A-B93, 27A-B4 and 30BB6) and *P. protegens* (44R-P8) is particularly broad. Interestingly, despite the systematic search for known bio-active compounds via biomolecular and *in vitro* approaches, a large set of strongly effective strains remained negative to these screenings. Although those approaches have intrinsic limitations, these results are particularly encouraging in the prospection of discovering novel bio-active metabolites.

Among the 22 *Bacillus* spp. strains positive for the bacilysin-related genes, 14 were very effective against *P. infestans* (GIP ≥ 50%). These observations suggest that (part of) the strong *in vitro* inhibition against *P. infestans* might be related to the production of bacilysin by some *B. pumilus* and *B. subtilis* strains. Bacilysin is a 270 Daltons dipeptide composed of L-alanine and L-anticapsin known for its antifungal activity through inhibition of fungal mannoprotein and chitin biosynthesis (Milewski et al., 1986). Moreover, bacilysin also has antibacterial activity since it is an inhibitor of glucosamine synthetase (Chmara, 1985) and hence interferes with the bacterial peptidoglycan biosynthesis. Therefore, it might interfere with cellulose biosynthesis which is required by *P. infestans* to successfully infect potato (Grenville-Briggs et al., 2008). The potential involvement of bacilysin in antagonistic interactions with the oomycete *Phytophthora capsici* suggested by Chung et al. (2008) has not been clearly demonstrated since the observed activity could be related to a blend of known antifungal metabolites as iturin or mersacidin. Therefore, the potential mode of action of bacilysin against oomycete pathogens should be further investigated.

Besides bacilysin, other bio-active compounds might be involved in antagonistic activity. Our results revealed that the *B. amyloliquefaciens, B. subtilis* and *Pseudomonas* spp. strains effective *in vitro* and *in vivo* against *P. infestans* (GIP ≥ 70%) were also able to produce both bio-surfactants and siderophores. Bio-surfactant as lipopeptides from iturin and fengycin families produced by *B. amyloliquefaciens* and *B. subtilis* species are known to exhibit direct antifungal and anti-oomycetal activity (Yu et al., 2002; Ongena et al., 2005; Raaijmakers et al., 2010). Their fungi-toxicity is mediated by mechanisms involving pore formation in the plasmic membrane (Maget-Dana et al., 1985; Ongena and Jacques, 2008). Similarly, cyclic lipopeptides (cLPS) produced by *Pseudomonas* spp. have direct antifungal and anti-oomycetal activities (Yang et al., 2014). Cyclic lipopeptides produced by *P. fluorescens* SBW25, for instance, have a specific activity on *P. infestans* zoospores (de Bruijn et al., 2007). Their exposure to cLPS results in their complete immobilization and subsequent lysis. The mechanism of action suggested, as for *Bacillus* lipopeptides, is a solubilization of the zoospore membranes. Among the five *Pseudomonas* strains selected in this work and highly active against *P. infestans*, three were shown to possess the genetic determinants involved in lipopeptide biosynthesis and all were positive to bio-surfactant production tests. Similar observations were done for *B. amyloliquefaciens* and *B. subtilis* strains regarding the presence of the genetic determinants coding for lipopeptides of fengycin, iturin, and surfactin families and they were positive to bio-surfactant production, too. Moreover, three of the active *Pseudomonas* spp. strains against *P. infestans* (i.e., 31A-P4, 42R-P6, and 48R-P9) were able to induce systemic resistance in *A. thaliana* (unpubl. results). Indeed, some lipopeptides produced by *Pseudomonas* spp., as well as lipopeptides from the surfactin and fengycin family produced by *Bacillus*, are known to have an indirect antagonistic activity triggering ISR in plant (Ongena et al., 2007; Tran et al., 2007; Jourdan et al., 2009; Nihorimbere et al., 2012). This indirect activity might be one explanation to the poor correlation observed between growth inhibition in *in vitro* assays and the protection index obtained in *in vivo* assays.

Lipopeptides are not the only metabolites able to trigger plant defenses. Iron chelating siderophores, produced either by *Bacillus* spp. or *Pseudomonas* spp., are also associated with indirect antagonistic mechanisms such as plant defenses and/or growth promotion (Leeman et al., 1996; de Boer et al., 2003; Meziane et al., 2005; Yu et al., 2011; Verbon et al., 2017). For example, pseudobactin siderophore produced by *P. putida* WCS358 is able to act as an elicitor and prime plant tissues in tomato for enhanced defenses against the pathogen *Botrytis cinerea* (Meziane et al., 2005). The ISR-triggering activity of cathecolic siderophore bacillibactin, produced by *B. subtilis* CAS15, has also been pointed out by Yu et al. (2011) on another *Solanaceae, Capscicum annuum* L., confronted to *Fusarium* wilt. However, siderophores with high and specific affinities are best known for their antagonistic activities through efficient competition for iron uptake, making it unavailable for pathogens (Kloepper et al., 1980; Thomashow and Bakker, 2015). Even though direct antifungal activity of the harzianic acid siderophore produced by *Trichoderma harzianum* has been highlighted (Vinale et al., 2013), to our knowledge, no direct activity of siderophores produced by *Pseudomonas* spp. or *Bacillus* spp. has been demonstrated. The difficulty to distinguish siderophores from common antibiotic definition has already been pointed out by Haas and Defago (2005). They suggested that siderophores contribute to disease suppression in some situations, but are not acting alone. Based on our observations, we suggest the complementary roles of lipopeptides and siderophores explaining the high *in vivo* antagonistic activity against the oomycete *P. infestans*. Indeed, among our core collection, all *Pseudomonas* spp., *B. amyloliquefaciens* (17A-B3), and *B. subtilis* (15A-B92, 27A-B4, 30B-B6) that were able to produce both siderophores and bio-surfactants, strongly inhibit *P. infestans*, whereas *B. licheniformis* strains, only producing siderophores, and *B. subtilis* that does not produce any of these metabolites, were not very effective against *P. infestans*. Therefore, considering the production of these two metabolites might be an interesting way of selecting effective bio-controlling agents (BCA) from *B. subtilis* and *P. fluorescens* groups. Moreover, a potential co-production, synergistic or any other complementary activities of those bio-active metabolites could also be a valuable explanation to effectiveness against late blight and should be further investigated.

This work also revealed the interest in other bio-control mechanisms dealing with competition for space, nutrient or oxygen between microorganisms and their ability to quickly colonize an ecological niche. This capacity is illustrated by the *in vitro* confrontation tests with strains of *B. mycoides* and their distinct rhizoid phenotype (Figure S2 and Table 2). Our results showed that *B. mycoides* strains have a strong *in vitro* antagonistic activity against *P. infestans*, which is thought to be mediated by competition for surface colonization. Additional assays with the lyophilized *B. mycoides* 18A-B9 strain, applied as tuber treatment, showed its ability to colonize “Bintje” potato plant from soil to upper foliar levels (unpubl. results). The same strain was also able to induce systemic resistance in *A. thaliana* (unpubl. results). This *Bacillus* species is already known in bio-control strategies as a plant defense and plant growth promoter (Bargabus et al., 2002; Bach et al., 2016). Similarly, *B. mycoides* 15A-B2 was very effective *in vivo* against *P. infestans* conferring a significant protection to “Bintje” potato plant. Under greenhouse conditions (foliar spraying), this strain also significantly decrease late blight severity (PI: 65%) which suggests that it is not only able to survive but could also colonize the foliar surface and allow a direct competition with *P. infestans* by invading its ecological niche and preventing infection of plant tissue. The production of anti-oomycete compounds at foliar level should not be excluded. Indeed, PCR screenings showed that the *B. mycoides* strains were almost exclusively positive to AHL-lactonase and exochitinase-related genes.

*Pseudomonas* spp. and *Bacillus* spp. bacteria are known to be producers of numerous bio-active compounds with versatile capacities but their *in vitro* activities are not easily transferable to *in vivo* assays, especially under field conditions (Ravensberg, 2011; Glare et al., 2012). This highlights the necessity to develop efficient way of selection. In the present study, *Bacillus* and *Pseudomonas* strains selected after extensive characterization for greenhouse trials showed promising, repeated and statistically relevant decrease in late blight severity going from 46% to almost 90%. Moreover, pilot field trial revealed the effectiveness of two out of four strains tested. *P. protegens* strain 44R-P8 conferred a significant protection (PI: 19%) against naturally occurring late blight until 16 days after first apparition of symptoms while *B. subtilis* 30B-B6 strain was still able to decrease from 44% the late blight severity and showed significant protection until the end of the pilot field trial (PI: 22%). These levels of protection are very promising considering the fact that no formulation was used and only crude bacterial suspensions were applied once a week, regardless of environmental conditions that were extremely severe, promoting *P. infestans* development and propagation.

Although treatments based on BCA are not sufficiently effective to be used alone, the application of *B. subtilis* 30B-B6 allowed a shift in time of 5 days to reach same level of late blight severity in first 2 weeks of infection. This shift in time could delay the first treatment with fungicides. Therefore, it would imply a decreasing in the number of required fungicide pulverizations that would be economically and environmentally attractive. However, developing a sustainable pest management approach must integrate various parameters such as appropriate crop rotation, appropriate fertilization, use of resistant cultivars, use of bio-control agents and pesticides, among others. In this regard, compatibility of active strains with specific cultivars and with a pest management scheme encompassing chemical pesticides are required and currently investigated. Taken together, this work highlights the benefit to combine molecular screening for bio-active metabolites with *in vitro* and greenhouse assays to successfully select field effective BCAs and have an insight in their potential mode of actions.

## Author contributions

CB, JM, and AL conceived the study. PM and ND performed sampling and preliminary *in vitro* screenings. SC, AG, FL, and ML completed antagonistic *in vitro* screenings. AG performed strains identification and analyzed sequencing data. AG and SC performed genetic screening for bio-active metabolites and virulence factors. SC performed *in vitro* screening for bio-active metabolites production. ML conducted plant assays in greenhouses. GC carried out pilot field trial. SC analyzed the data of *in vitro* screenings, genetic screenings, plant assays and pilot field trial. SC and AG wrote the manuscript with help of AL, JM, and CB. All authors have read and approved the final manuscript.

### Conflict of interest statement

The authors declare that the research was conducted in the absence of any commercial or financial relationships that could be construed as a potential conflict of interest.
