# Supplementary material for: Versatile Antagonistic Activities of Soil-Borne Bacillus spp. and Pseudomonas spp. against Phytophthora infestans and Other Potato Pathogens
Source: Front Microbiol. 2018 Feb 13;9:143. doi: 10.3389/fmicb.2018.00143 (PMC5816801; doi:10.3389/fmicb.2018.00143)
Supplement: Supplementary file 1 [file SupplementaryTables.DOCX]

**Table S1.** Bacterial isolates used in this study with their corresponding sampling site in Belgium, GPS coordinates and sample site type.

| **Isolates^*^** | **Sampling Site** | **GPS coordinates** | **Sample Type** |
| --- | --- | --- | --- |
| 1A-B1 | Tubize | 50.699556,4.194803 | Crop field |
| 1A-B2 | Tubize | 50.699556,4.194803 | Crop field |
| 1A-B3 | Tubize | 50.699556,4.194803 | Crop field |
| 1A-B4 | Tubize | 50.699556,4.194803 | Crop field |
| 1A-B5 | Tubize | 50.699556,4.194803 | Crop field |
| 1A-B6 | Tubize | 50.699556,4.194803 | Crop field |
| 1A-B7 | Tubize | 50.699556,4.194803 | Crop field |
| 1A-B8 | Tubize | 50.699556,4.194803 | Crop field |
| 1A-B9 | Tubize | 50.699556,4.194803 | Crop field |
| 2A-B1 | Tubize | 50.700073,4.195576 | Crop field |
| 2A-B2 | Tubize | 50.700073,4.195576 | Crop field |
| 2A-B3 | Tubize | 50.700073,4.195576 | Crop field |
| 2A-B4 | Tubize | 50.700073,4.195576 | Crop field |
| 2A-B5 | Tubize | 50.700073,4.195576 | Crop field |
| 2A-B6 | Tubize | 50.700073,4.195576 | Crop field |
| 2A-B7 | Tubize | 50.700073,4.195576 | Crop field |
| 2A-B8 | Tubize | 50.700073,4.195576 | Crop field |
| 2A-B9 | Tubize | 50.700073,4.195576 | Crop field |
| 3A-B1 | Rebecq | 50.642167,4.145193 | Crop field |
| 3A-B2 | Rebecq | 50.642167,4.145193 | Crop field |
| 3A-B3 | Rebecq | 50.642167,4.145193 | Crop field |
| 3A-B4 | Rebecq | 50.642167,4.145193 | Crop field |
| 3A-B5 | Rebecq | 50.642167,4.145193 | Crop field |
| 3A-B6 | Rebecq | 50.642167,4.145193 | Crop field |
| 3A-B7 | Rebecq | 50.642167,4.145193 | Crop field |
| 3A-B8 | Rebecq | 50.642167,4.145193 | Crop field |
| 3A-B9 | Rebecq | 50.642167,4.145193 | Crop field |
| 4A-B1 | Perwez | 50.624392,4.826646 | Crop field |
| 4A-B2 | Perwez | 50.624392,4.826646 | Crop field |
| 4A-B3 | Perwez | 50.624392,4.826646 | Crop field |
| 4A-B4 | Perwez | 50.624392,4.826646 | Crop field |
| 4A-B5 | Perwez | 50.624392,4.826646 | Crop field |
| 4A-B6 | Perwez | 50.624392,4.826646 | Crop field |
| 4A-B7 | Perwez | 50.624392,4.826646 | Crop field |
| 4A-B8 | Perwez | 50.624392,4.826646 | Crop field |
| 4A-B9 | Perwez | 50.624392,4.826646 | Crop field |
| 5A-B1 | Incourt | 50.683843,4.79167 | Crop field |
| 5A-B2 | Incourt | 50.683843,4.79167 | Crop field |
| 5A-B3 | Incourt | 50.683843,4.79167 | Crop field |
| 5A-B4 | Incourt | 50.683843,4.79167 | Crop field |
| 5A-B5 | Incourt | 50.683843,4.79167 | Crop field |
| 5A-B6 | Incourt | 50.683843,4.79167 | Crop field |
| 5A-B7 | Incourt | 50.683843,4.79167 | Crop field |
| 5A-B8 | Incourt | 50.683843,4.79167 | Crop field |
| 5A-B9 | Incourt | 50.683843,4.79167 | Crop field |
| 6A-B1 | Incourt | 50.67135,4.81096 | Crop field |
| 6A-B2 | Incourt | 50.67135,4.81096 | Crop field |
| 6A-B3 | Incourt | 50.67135,4.81096 | Crop field |
| 6A-B4 | Incourt | 50.67135,4.81096 | Crop field |
| 6A-B5 | Incourt | 50.67135,4.81096 | Crop field |
| 6A-B6 | Incourt | 50.67135,4.81096 | Crop field |
| 6A-B7 | Incourt | 50.67135,4.81096 | Crop field |
| 6A-B8 | Incourt | 50.67135,4.81096 | Crop field |
| 6A-B9 | Incourt | 50.67135,4.81096 | Crop field |
| 7A-B1 | Gembloux | 50.572922,4.705045 | Crop field |
| 7A-B2 | Gembloux | 50.572922,4.705045 | Crop field |
| 7A-B3 | Gembloux | 50.572922,4.705045 | Crop field |
| 7A-B4 | Gembloux | 50.572922,4.705045 | Crop field |
| 7A-B5 | Gembloux | 50.572922,4.705045 | Crop field |
| 7A-B6 | Gembloux | 50.572922,4.705045 | Crop field |
| 7A-B7 | Gembloux | 50.572922,4.705045 | Crop field |
| 7A-B8 | Gembloux | 50.572922,4.705045 | Crop field |
| 7A-B9 | Gembloux | 50.572922,4.705045 | Crop field |
| 8A-B1 | Gembloux | 50.594653,4.732018 | Crop field |
| 8A-B2 | Gembloux | 50.594653,4.732018 | Crop field |
| 8A-B3 | Gembloux | 50.594653,4.732018 | Crop field |
| 8A-B4 | Gembloux | 50.594653,4.732018 | Crop field |
| 8A-B5 | Gembloux | 50.594653,4.732018 | Crop field |
| 8A-B6 | Gembloux | 50.594653,4.732018 | Crop field |
| 8A-B7 | Gembloux | 50.594653,4.732018 | Crop field |
| 8A-B8 | Gembloux | 50.594653,4.732018 | Crop field |
| 8A-B9 | Gembloux | 50.594653,4.732018 | Crop field |
| 9A-B1 | Gembloux | 50.600537,4.732275 | Crop field |
| 9A-B2 | Gembloux | 50.600537,4.732275 | Crop field |
| 9A-B3 | Gembloux | 50.600537,4.732275 | Crop field |
| 9A-B4 | Gembloux | 50.600537,4.732275 | Crop field |
| 9A-B5 | Gembloux | 50.600537,4.732275 | Crop field |
| 9A-B6 | Gembloux | 50.600537,4.732275 | Crop field |
| 9A-B7 | Gembloux | 50.600537,4.732275 | Crop field |
| 9A-B8 | Gembloux | 50.600537,4.732275 | Crop field |
| 9A-B9 | Gembloux | 50.600537,4.732275 | Crop field |
| 10A-B1 | Gembloux | 50.599829,4.733176 | Crop field |
| 10A-B2 | Gembloux | 50.599829,4.733176 | Crop field |
| 10A-B3 | Gembloux | 50.599829,4.733176 | Crop field |
| 10A-B4 | Gembloux | 50.599829,4.733176 | Crop field |
| 10A-B5 | Gembloux | 50.599829,4.733176 | Crop field |
| 10A-B6 | Gembloux | 50.599829,4.733176 | Crop field |
| 10A-B7 | Gembloux | 50.599829,4.733176 | Crop field |
| 10A-B8 | Gembloux | 50.599829,4.733176 | Crop field |
| 10A-B9 | Gembloux | 50.599829,4.733176 | Crop field |
| 11A-B1 | Gembloux | 50.569269,4.670348 | Crop field |
| 11A-B2 | Gembloux | 50.569269,4.670348 | Crop field |
| 11A-B3 | Gembloux | 50.569269,4.670348 | Crop field |
| 11A-B4 | Gembloux | 50.569269,4.670348 | Crop field |
| 11A-B5 | Gembloux | 50.569269,4.670348 | Crop field |
| 11A-B6 | Gembloux | 50.569269,4.670348 | Crop field |
| 11A-B7 | Gembloux | 50.569269,4.670348 | Crop field |
| 11A-B8 | Gembloux | 50.569269,4.670348 | Crop field |
| 11A-B9 | Gembloux | 50.569269,4.670348 | Crop field |
| 12A-B1 | Gembloux | 50.568969,4.671357 | Crop field |
| 12A-B2 | Gembloux | 50.568969,4.671357 | Crop field |
| 12A-B3 | Gembloux | 50.568969,4.671357 | Crop field |
| 12A-B4 | Gembloux | 50.568969,4.671357 | Crop field |
| 12A-B5 | Gembloux | 50.568969,4.671357 | Crop field |
| 12A-B6 | Gembloux | 50.568969,4.671357 | Crop field |
| 12A-B7 | Gembloux | 50.568969,4.671357 | Crop field |
| 12A-B8 | Gembloux | 50.568969,4.671357 | Crop field |
| 12A-B9 | Gembloux | 50.568969,4.671357 | Crop field |
| 13A-B1 | Gembloux | 50.601054,4.86866 | Crop field |
| 13A-B2 | Gembloux | 50.601054,4.86866 | Crop field |
| 13A-B3 | Gembloux | 50.601054,4.86866 | Crop field |
| 13A-B4 | Gembloux | 50.601054,4.86866 | Crop field |
| 13A-B5 | Gembloux | 50.601054,4.86866 | Crop field |
| 13A-B6 | Gembloux | 50.601054,4.86866 | Crop field |
| 13A-B7 | Gembloux | 50.601054,4.86866 | Crop field |
| 13A-B8 | Gembloux | 50.601054,4.86866 | Crop field |
| 13A-B9 | Gembloux | 50.601054,4.86866 | Crop field |
| 14A-B1 | Cognelée | 50.528304,4.899151 | Crop field |
| 14A-B2 | Cognelée | 50.528304,4.899151 | Crop field |
| 14A-B3 | Cognelée | 50.528304,4.899151 | Crop field |
| 14A-B4 | Cognelée | 50.528304,4.899151 | Crop field |
| 14A-B5 | Cognelée | 50.528304,4.899151 | Crop field |
| 14A-B6 | Cognelée | 50.528304,4.899151 | Crop field |
| 14A-B7 | Cognelée | 50.528304,4.899151 | Crop field |
| 14A-B8 | Cognelée | 50.528304,4.899151 | Crop field |
| 14A-B9 | Cognelée | 50.528304,4.899151 | Crop field |
| 15A-B1 | Bousval | 50.614672,4.494953 | Crop field |
| 15A-B2 | Bousval | 50.614672,4.494953 | Crop field |
| 15A-B3 | Bousval | 50.614672,4.494953 | Crop field |
| 15A-B4 | Bousval | 50.614672,4.494953 | Crop field |
| 15A-B5 | Bousval | 50.614672,4.494953 | Crop field |
| 15A-B6 | Bousval | 50.614672,4.494953 | Crop field |
| 15A-B7 | Bousval | 50.614672,4.494953 | Crop field |
| 15A-B8 | Bousval | 50.614672,4.494953 | Crop field |
| 15A-B9 | Bousval | 50.614672,4.494953 | Crop field |
| 16A-B1 | Court st Etienne | 50.632505,4.550829 | Crop field |
| 16A-B2 | Court st Etienne | 50.632505,4.550829 | Crop field |
| 16A-B3 | Court st Etienne | 50.632505,4.550829 | Crop field |
| 16A-B4 | Court st Etienne | 50.632505,4.550829 | Crop field |
| 16A-B5 | Court st Etienne | 50.632505,4.550829 | Crop field |
| 16A-B6 | Court st Etienne | 50.632505,4.550829 | Crop field |
| 16A-B7 | Court st Etienne | 50.632505,4.550829 | Crop field |
| 16A-B8 | Court st Etienne | 50.632505,4.550829 | Crop field |
| 16A-B9 | Court st Etienne | 50.632505,4.550829 | Crop field |
| 17A-B1 | Court st Etienne | 50.630001,4.551762 | Crop field |
| 17A-B2 | Court st Etienne | 50.630001,4.551762 | Crop field |
| 17A-B3 | Court st Etienne | 50.630001,4.551762 | Crop field |
| 17A-B4 | Court st Etienne | 50.630001,4.551762 | Crop field |
| 17A-B5 | Court st Etienne | 50.630001,4.551762 | Crop field |
| 17A-B6 | Court st Etienne | 50.630001,4.551762 | Crop field |
| 17A-B7 | Court st Etienne | 50.630001,4.551762 | Crop field |
| 17A-B8 | Court st Etienne | 50.630001,4.551762 | Crop field |
| 17A-B9 | Court st Etienne | 50.630001,4.551762 | Crop field |
| 18A-B1 | Louvain-la-Neuve | 50.675793,4.624686 | Crop field |
| 18A-B2 | Louvain-la-Neuve | 50.675793,4.624686 | Crop field |
| 18A-B3 | Louvain-la-Neuve | 50.675793,4.624686 | Crop field |
| 18A-B4 | Louvain-la-Neuve | 50.675793,4.624686 | Crop field |
| 18A-B5 | Louvain-la-Neuve | 50.675793,4.624686 | Crop field |
| 18A-B6 | Louvain-la-Neuve | 50.675793,4.624686 | Crop field |
| 18A-B7 | Louvain-la-Neuve | 50.675793,4.624686 | Crop field |
| 18A-B8 | Louvain-la-Neuve | 50.675793,4.624686 | Crop field |
| 18A-B9 | Louvain-la-Neuve | 50.675793,4.624686 | Crop field |
| 19A-B1 | Coxyde | 51.104452,2.660258 | Crop field |
| 19A-B2 | Coxyde | 51.104452,2.660258 | Crop field |
| 19A-B3 | Coxyde | 51.104452,2.660258 | Crop field |
| 19A-B4 | Coxyde | 51.104452,2.660258 | Crop field |
| 19A-B5 | Coxyde | 51.104452,2.660258 | Crop field |
| 19A-B6 | Coxyde | 51.104452,2.660258 | Crop field |
| 19A-B7 | Coxyde | 51.104452,2.660258 | Crop field |
| 19A-B8 | Coxyde | 51.104452,2.660258 | Crop field |
| 19A-B9 | Coxyde | 51.104452,2.660258 | Crop field |
| 20A-B1 | Coxyde | 51.089204,2.636547 | Crop field |
| 20A-B2 | Coxyde | 51.089204,2.636547 | Crop field |
| 20A-B3 | Coxyde | 51.089204,2.636547 | Crop field |
| 20A-B4 | Coxyde | 51.089204,2.636547 | Crop field |
| 20A-B5 | Coxyde | 51.089204,2.636547 | Crop field |
| 20A-B6 | Coxyde | 51.089204,2.636547 | Crop field |
| 20A-B7 | Coxyde | 51.089204,2.636547 | Crop field |
| 20A-B8 | Coxyde | 51.089204,2.636547 | Crop field |
| 20A-B9 | Coxyde | 51.089204,2.636547 | Crop field |
| 21A-B1 | Coxyde | 51.090073,2.636118 | Crop field |
| 21A-B2 | Coxyde | 51.090073,2.636118 | Crop field |
| 21A-B3 | Coxyde | 51.090073,2.636118 | Crop field |
| 21A-B4 | Coxyde | 51.090073,2.636118 | Crop field |
| 21A-B5 | Coxyde | 51.090073,2.636118 | Crop field |
| 21A-B6 | Coxyde | 51.090073,2.636118 | Crop field |
| 21A-B7 | Coxyde | 51.090073,2.636118 | Crop field |
| 21A-B8 | Coxyde | 51.090073,2.636118 | Crop field |
| 21A-B9 | Coxyde | 51.090073,2.636118 | Crop field |
| 22A-B1 | Furne | 51.059489,2.663519 | Crop field |
| 22A-B2 | Furne | 51.059489,2.663519 | Crop field |
| 22A-B3 | Furne | 51.059489,2.663519 | Crop field |
| 22A-B4 | Furne | 51.059489,2.663519 | Crop field |
| 22A-B5 | Furne | 51.059489,2.663519 | Crop field |
| 22A-B6 | Furne | 51.059489,2.663519 | Crop field |
| 22A-B7 | Furne | 51.059489,2.663519 | Crop field |
| 22A-B8 | Furne | 51.059489,2.663519 | Crop field |
| 22A-B9 | Furne | 51.059489,2.663519 | Crop field |
| 23A-B1 | Furne | 51.070284,2.7088 | Crop field |
| 23A-B2 | Furne | 51.070284,2.7088 | Crop field |
| 23A-B3 | Furne | 51.070284,2.7088 | Crop field |
| 23A-B4 | Furne | 51.070284,2.7088 | Crop field |
| 23A-B5 | Furne | 51.070284,2.7088 | Crop field |
| 23A-B6 | Furne | 51.070284,2.7088 | Crop field |
| 23A-B7 | Furne | 51.070284,2.7088 | Crop field |
| 23A-B8 | Furne | 51.070284,2.7088 | Crop field |
| 23A-B9 | Furne | 51.070284,2.7088 | Crop field |
| 24A-B1 | Furne | 51.070122,2.709353 | Crop field |
| 24A-B2 | Furne | 51.070122,2.709353 | Crop field |
| 24A-B3 | Furne | 51.070122,2.709353 | Crop field |
| 24A-B4 | Furne | 51.070122,2.709353 | Crop field |
| 24A-B5 | Furne | 51.070122,2.709353 | Crop field |
| 24A-B6 | Furne | 51.070122,2.709353 | Crop field |
| 24A-B7 | Furne | 51.070122,2.709353 | Crop field |
| 24A-B8 | Furne | 51.070122,2.709353 | Crop field |
| 24A-B9 | Furne | 51.070122,2.709353 | Crop field |
| 25A-B1 | Furne | 51.063488,2.767482 | Crop field |
| 25A-B2 | Furne | 51.063488,2.767482 | Crop field |
| 25A-B3 | Furne | 51.063488,2.767482 | Crop field |
| 25A-B4 | Furne | 51.063488,2.767482 | Crop field |
| 25A-B5 | Furne | 51.063488,2.767482 | Crop field |
| 25A-B6 | Furne | 51.063488,2.767482 | Crop field |
| 25A-B7 | Furne | 51.063488,2.767482 | Crop field |
| 25A-B8 | Furne | 51.063488,2.767482 | Crop field |
| 25A-B9 | Furne | 51.063488,2.767482 | Crop field |
| 26A-B1 | Furne | 51.06338,2.768469 | Crop field |
| 26A-B2 | Furne | 51.06338,2.768469 | Crop field |
| 26A-B3 | Furne | 51.06338,2.768469 | Crop field |
| 26A-B4 | Furne | 51.06338,2.768469 | Crop field |
| 26A-B5 | Furne | 51.06338,2.768469 | Crop field |
| 26A-B6 | Furne | 51.06338,2.768469 | Crop field |
| 26A-B7 | Furne | 51.06338,2.768469 | Crop field |
| 26A-B8 | Furne | 51.06338,2.768469 | Crop field |
| 26A-B9 | Furne | 51.06338,2.768469 | Crop field |
| 27A-B1 | Waremme | n/a | Crop field |
| 27A-B2 | Waremme | n/a | Crop field |
| 27A-B3 | Waremme | n/a | Crop field |
| 27A-B4 | Waremme | n/a | Crop field |
| 27A-B5 | Waremme | n/a | Crop field |
| 27A-B6 | Waremme | n/a | Crop field |
| 27A-B7 | Waremme | n/a | Crop field |
| 27A-B8 | Waremme | n/a | Crop field |
| 27A-B9 | Waremme | n/a | Crop field |
| 28A-B1 | Waremme | n/a | Crop field |
| 28A-B2 | Waremme | n/a | Crop field |
| 28A-B3 | Waremme | n/a | Crop field |
| 28A-B4 | Waremme | n/a | Crop field |
| 28A-B5 | Waremme | n/a | Crop field |
| 28A-B6 | Waremme | n/a | Crop field |
| 28A-B7 | Waremme | n/a | Crop field |
| 28A-B8 | Waremme | n/a | Crop field |
| 28A-B9 | Waremme | n/a | Crop field |
| 29A-B1 | Waremme | n/a | Crop field |
| 29A-B2 | Waremme | n/a | Crop field |
| 29A-B3 | Waremme | n/a | Crop field |
| 29A-B4 | Waremme | n/a | Crop field |
| 29A-B5 | Waremme | n/a | Crop field |
| 29A-B6 | Waremme | n/a | Crop field |
| 29A-B7 | Waremme | n/a | Crop field |
| 29A-B8 | Waremme | n/a | Crop field |
| 29A-B9 | Waremme | n/a | Crop field |
| 30A-B1 | Waremme | n/a | Crop field |
| 30A-B2 | Waremme | n/a | Crop field |
| 30A-B3 | Waremme | n/a | Crop field |
| 30A-B4 | Waremme | n/a | Crop field |
| 30A-B5 | Waremme | n/a | Crop field |
| 30A-B6 | Waremme | n/a | Crop field |
| 30A-B7 | Waremme | n/a | Crop field |
| 30A-B8 | Waremme | n/a | Crop field |
| 30A-B9 | Waremme | n/a | Crop field |
| 31A-B1 | Soumagne | 50.652113,5.712376 | Crop field |
| 31A-B2 | Soumagne | 50.652113,5.712376 | Crop field |
| 31A-B3 | Soumagne | 50.652113,5.712376 | Crop field |
| 31A-B4 | Soumagne | 50.652113,5.712376 | Crop field |
| 31A-B5 | Soumagne | 50.652113,5.712376 | Crop field |
| 31A-B6 | Soumagne | 50.652113,5.712376 | Crop field |
| 31A-B7 | Soumagne | 50.652113,5.712376 | Crop field |
| 31A-B8 | Soumagne | 50.652113,5.712376 | Crop field |
| 31A-B9 | Soumagne | 50.652113,5.712376 | Crop field |
| 32A-B1 | Soumagne | 50.632832,5.703814 | Crop field |
| 32A-B2 | Soumagne | 50.632832,5.703814 | Crop field |
| 32A-B3 | Soumagne | 50.632832,5.703814 | Crop field |
| 32A-B4 | Soumagne | 50.632832,5.703814 | Crop field |
| 32A-B5 | Soumagne | 50.632832,5.703814 | Crop field |
| 32A-B6 | Soumagne | 50.632832,5.703814 | Crop field |
| 32A-B7 | Soumagne | 50.632832,5.703814 | Crop field |
| 32A-B8 | Soumagne | 50.632832,5.703814 | Crop field |
| 32A-B9 | Soumagne | 50.632832,5.703814 | Crop field |
| 33A-B1 | Soumagne | 50.663935,5.697779 | Crop field |
| 33A-B2 | Soumagne | 50.663935,5.697779 | Crop field |
| 33A-B3 | Soumagne | 50.663935,5.697779 | Crop field |
| 33A-B4 | Soumagne | 50.663935,5.697779 | Crop field |
| 33A-B5 | Soumagne | 50.663935,5.697779 | Crop field |
| 33A-B6 | Soumagne | 50.663935,5.697779 | Crop field |
| 33A-B7 | Soumagne | 50.663935,5.697779 | Crop field |
| 33A-B8 | Soumagne | 50.663935,5.697779 | Crop field |
| 33A-B9 | Soumagne | 50.663935,5.697779 | Crop field |
| 34A-B1 | Soumagne | 50.661326,5.673505 | Crop field |
| 34A-B2 | Soumagne | 50.661326,5.673505 | Crop field |
| 34A-B3 | Soumagne | 50.661326,5.673505 | Crop field |
| 34A-B4 | Soumagne | 50.661326,5.673505 | Crop field |
| 34A-B5 | Soumagne | 50.661326,5.673505 | Crop field |
| 34A-B6 | Soumagne | 50.661326,5.673505 | Crop field |
| 34A-B7 | Soumagne | 50.661326,5.673505 | Crop field |
| 34A-B8 | Soumagne | 50.661326,5.673505 | Crop field |
| 34A-B9 | Soumagne | 50.661326,5.673505 | Crop field |
| 35A-B1 | Soumagne | 50.660867,5.674632 | Crop field |
| 35A-B2 | Soumagne | 50.660867,5.674632 | Crop field |
| 35A-B3 | Soumagne | 50.660867,5.674632 | Crop field |
| 35A-B4 | Soumagne | 50.660867,5.674632 | Crop field |
| 35A-B5 | Soumagne | 50.660867,5.674632 | Crop field |
| 35A-B6 | Soumagne | 50.660867,5.674632 | Crop field |
| 35A-B7 | Soumagne | 50.660867,5.674632 | Crop field |
| 35A-B8 | Soumagne | 50.660867,5.674632 | Crop field |
| 35A-B9 | Soumagne | 50.660867,5.674632 | Crop field |
| 41A-B1 | Bertrix | 49.848617,5.239266 | Crop field |
| 41A-B2 | Bertrix | 49.848617,5.239266 | Crop field |
| 41A-B3 | Bertrix | 49.848617,5.239266 | Crop field |
| 41A-B4 | Bertrix | 49.848617,5.239266 | Crop field |
| 41A-B5 | Bertrix | 49.848617,5.239266 | Crop field |
| 41A-B6 | Bertrix | 49.848617,5.239266 | Crop field |
| 41A-B7 | Bertrix | 49.848617,5.239266 | Crop field |
| 41A-B8 | Bertrix | 49.848617,5.239266 | Crop field |
| 41A-B9 | Bertrix | 49.848617,5.239266 | Crop field |
| 42A-B1 | Bertrix | 49.85128,5.25257 | Crop field |
| 42A-B2 | Bertrix | 49.85128,5.25257 | Crop field |
| 42A-B3 | Bertrix | 49.85128,5.25257 | Crop field |
| 42A-B4 | Bertrix | 49.85128,5.25257 | Crop field |
| 42A-B5 | Bertrix | 49.85128,5.25257 | Crop field |
| 42A-B6 | Bertrix | 49.85128,5.25257 | Crop field |
| 42A-B7 | Bertrix | 49.85128,5.25257 | Crop field |
| 42A-B8 | Bertrix | 49.85128,5.25257 | Crop field |
| 42A-B9 | Bertrix | 49.85128,5.25257 | Crop field |
| 43A-B1 | Bertrix | 49.85128,5.25257 | Crop field |
| 43A-B2 | Bertrix | 49.85128,5.25257 | Crop field |
| 43A-B3 | Bertrix | 49.85128,5.25257 | Crop field |
| 43A-B4 | Bertrix | 49.85128,5.25257 | Crop field |
| 43A-B5 | Bertrix | 49.85128,5.25257 | Crop field |
| 43A-B6 | Bertrix | 49.85128,5.25257 | Crop field |
| 43A-B7 | Bertrix | 49.85128,5.25257 | Crop field |
| 43A-B8 | Bertrix | 49.85128,5.25257 | Crop field |
| 43A-B9 | Bertrix | 49.85128,5.25257 | Crop field |
| 44A-B1 | Bertrix | 49.84403,5.265884 | Crop field |
| 44A-B2 | Bertrix | 49.84403,5.265884 | Crop field |
| 44A-B3 | Bertrix | 49.84403,5.265884 | Crop field |
| 44A-B4 | Bertrix | 49.84403,5.265884 | Crop field |
| 44A-B5 | Bertrix | 49.84403,5.265884 | Crop field |
| 44A-B6 | Bertrix | 49.84403,5.265884 | Crop field |
| 44A-B7 | Bertrix | 49.84403,5.265884 | Crop field |
| 44A-B8 | Bertrix | 49.84403,5.265884 | Crop field |
| 44A-B9 | Bertrix | 49.84403,5.265884 | Crop field |
| 45A-B1 | Bertrix | 49.782615,5.213131 | Crop field |
| 45A-B2 | Bertrix | 49.782615,5.213131 | Crop field |
| 45A-B3 | Bertrix | 49.782615,5.213131 | Crop field |
| 45A-B4 | Bertrix | 49.782615,5.213131 | Crop field |
| 45A-B5 | Bertrix | 49.782615,5.213131 | Crop field |
| 45A-B6 | Bertrix | 49.782615,5.213131 | Crop field |
| 45A-B7 | Bertrix | 49.782615,5.213131 | Crop field |
| 45A-B8 | Bertrix | 49.782615,5.213131 | Crop field |
| 45A-B9 | Bertrix | 49.782615,5.213131 | Crop field |
| 46A-B1 | Ath | 50.645821,3.856544 | Crop field |
| 46A-B2 | Ath | 50.645821,3.856544 | Crop field |
| 46A-B3 | Ath | 50.645821,3.856544 | Crop field |
| 46A-B4 | Ath | 50.645821,3.856544 | Crop field |
| 46A-B5 | Ath | 50.645821,3.856544 | Crop field |
| 46A-B6 | Ath | 50.645821,3.856544 | Crop field |
| 46A-B7 | Ath | 50.645821,3.856544 | Crop field |
| 46A-B8 | Ath | 50.645821,3.856544 | Crop field |
| 46A-B9 | Ath | 50.645821,3.856544 | Crop field |
| 47A-B1 | Lens | 50.586615,3.906841 | Crop field |
| 47A-B2 | Lens | 50.586615,3.906841 | Crop field |
| 47A-B3 | Lens | 50.586615,3.906841 | Crop field |
| 47A-B4 | Lens | 50.586615,3.906841 | Crop field |
| 47A-B5 | Lens | 50.586615,3.906841 | Crop field |
| 47A-B6 | Lens | 50.586615,3.906841 | Crop field |
| 47A-B7 | Lens | 50.586615,3.906841 | Crop field |
| 47A-B8 | Lens | 50.586615,3.906841 | Crop field |
| 47A-B9 | Lens | 50.586615,3.906841 | Crop field |
| 48A-B1 | Soignies | 50.563978,4.017375 | Crop field |
| 48A-B2 | Soignies | 50.563978,4.017375 | Crop field |
| 48A-B3 | Soignies | 50.563978,4.017375 | Crop field |
| 48A-B4 | Soignies | 50.563978,4.017375 | Crop field |
| 48A-B5 | Soignies | 50.563978,4.017375 | Crop field |
| 48A-B6 | Soignies | 50.563978,4.017375 | Crop field |
| 48A-B7 | Soignies | 50.563978,4.017375 | Crop field |
| 48A-B8 | Soignies | 50.563978,4.017375 | Crop field |
| 48A-B9 | Soignies | 50.563978,4.017375 | Crop field |
| 49A-B1 | Chièvres | 50.550877,3.825468 | Crop field |
| 49A-B2 | Chièvres | 50.550877,3.825468 | Crop field |
| 49A-B3 | Chièvres | 50.550877,3.825468 | Crop field |
| 49A-B4 | Chièvres | 50.550877,3.825468 | Crop field |
| 49A-B5 | Chièvres | 50.550877,3.825468 | Crop field |
| 49A-B6 | Chièvres | 50.550877,3.825468 | Crop field |
| 49A-B7 | Chièvres | 50.550877,3.825468 | Crop field |
| 49A-B8 | Chièvres | 50.550877,3.825468 | Crop field |
| 49A-B9 | Chièvres | 50.550877,3.825468 | Crop field |
| 50A-B1 | Ath | 50.595933,3.710418 | Crop field |
| 50A-B2 | Ath | 50.595933,3.710418 | Crop field |
| 50A-B3 | Ath | 50.595933,3.710418 | Crop field |
| 50A-B4 | Ath | 50.595933,3.710418 | Crop field |
| 50A-B5 | Ath | 50.595933,3.710418 | Crop field |
| 50A-B6 | Ath | 50.595933,3.710418 | Crop field |
| 50A-B7 | Ath | 50.595933,3.710418 | Crop field |
| 50A-B8 | Ath | 50.595933,3.710418 | Crop field |
| 50A-B9 | Ath | 50.595933,3.710418 | Crop field |
| 51A-B1 | Habay | 49.737759,5.616503 | Crop field |
| 51A-B2 | Habay | 49.737759,5.616503 | Crop field |
| 51A-B3 | Habay | 49.737759,5.616503 | Crop field |
| 51A-B4 | Habay | 49.737759,5.616503 | Crop field |
| 51A-B5 | Habay | 49.737759,5.616503 | Crop field |
| 51A-B6 | Habay | 49.737759,5.616503 | Crop field |
| 51A-B7 | Habay | 49.737759,5.616503 | Crop field |
| 51A-B8 | Habay | 49.737759,5.616503 | Crop field |
| 51A-B9 | Habay | 49.737759,5.616503 | Crop field |
| 52A-B1 | Habay | 49.737829,5.615934 | Crop field |
| 52A-B2 | Habay | 49.737829,5.615934 | Crop field |
| 52A-B3 | Habay | 49.737829,5.615934 | Crop field |
| 52A-B4 | Habay | 49.737829,5.615934 | Crop field |
| 52A-B5 | Habay | 49.737829,5.615934 | Crop field |
| 52A-B6 | Habay | 49.737829,5.615934 | Crop field |
| 52A-B7 | Habay | 49.737829,5.615934 | Crop field |
| 52A-B8 | Habay | 49.737829,5.615934 | Crop field |
| 52A-B9 | Habay | 49.737829,5.615934 | Crop field |
| 53A-B1 | Habay | 49.724403,5.601751 | Crop field |
| 53A-B2 | Habay | 49.724403,5.601751 | Crop field |
| 53A-B3 | Habay | 49.724403,5.601751 | Crop field |
| 53A-B4 | Habay | 49.724403,5.601751 | Crop field |
| 53A-B5 | Habay | 49.724403,5.601751 | Crop field |
| 53A-B6 | Habay | 49.724403,5.601751 | Crop field |
| 53A-B7 | Habay | 49.724403,5.601751 | Crop field |
| 53A-B8 | Habay | 49.724403,5.601751 | Crop field |
| 53A-B9 | Habay | 49.724403,5.601751 | Crop field |
| 1B-B1 | Tubize | 50.699556,4.194803 | Crop field |
| 1B-B2 | Tubize | 50.699556,4.194803 | Crop field |
| 1B-B3 | Tubize | 50.699556,4.194803 | Crop field |
| 1B-B4 | Tubize | 50.699556,4.194803 | Crop field |
| 1B-B5 | Tubize | 50.699556,4.194803 | Crop field |
| 1B-B6 | Tubize | 50.699556,4.194803 | Crop field |
| 1B-B7 | Tubize | 50.699556,4.194803 | Crop field |
| 1B-B8 | Tubize | 50.699556,4.194803 | Crop field |
| 1B-B9 | Tubize | 50.699556,4.194803 | Crop field |
| 2B-B1 | Tubize | 50.700073,4.195576 | Crop field |
| 2B-B2 | Tubize | 50.700073,4.195576 | Crop field |
| 2B-B3 | Tubize | 50.700073,4.195576 | Crop field |
| 2B-B4 | Tubize | 50.700073,4.195576 | Crop field |
| 2B-B5 | Tubize | 50.700073,4.195576 | Crop field |
| 2B-B6 | Tubize | 50.700073,4.195576 | Crop field |
| 2B-B7 | Tubize | 50.700073,4.195576 | Crop field |
| 2B-B8 | Tubize | 50.700073,4.195576 | Crop field |
| 2B-B9 | Tubize | 50.700073,4.195576 | Crop field |
| 3B-B1 | Rebecq | 50.642167,4.145193 | Crop field |
| 3B-B2 | Rebecq | 50.642167,4.145193 | Crop field |
| 3B-B3 | Rebecq | 50.642167,4.145193 | Crop field |
| 3B-B4 | Rebecq | 50.642167,4.145193 | Crop field |
| 3B-B5 | Rebecq | 50.642167,4.145193 | Crop field |
| 3B-B6 | Rebecq | 50.642167,4.145193 | Crop field |
| 3B-B7 | Rebecq | 50.642167,4.145193 | Crop field |
| 3B-B8 | Rebecq | 50.642167,4.145193 | Crop field |
| 3B-B9 | Rebecq | 50.642167,4.145193 | Crop field |
| 4B-B1 | Perwez | 50.624392,4.826646 | Crop field |
| 4B-B2 | Perwez | 50.624392,4.826646 | Crop field |
| 4B-B3 | Perwez | 50.624392,4.826646 | Crop field |
| 4B-B4 | Perwez | 50.624392,4.826646 | Crop field |
| 4B-B5 | Perwez | 50.624392,4.826646 | Crop field |
| 4B-B6 | Perwez | 50.624392,4.826646 | Crop field |
| 4B-B7 | Perwez | 50.624392,4.826646 | Crop field |
| 4B-B8 | Perwez | 50.624392,4.826646 | Crop field |
| 4B-B9 | Perwez | 50.624392,4.826646 | Crop field |
| 5B-B1 | Incourt | 50.683843,4.79167 | Crop field |
| 5B-B2 | Incourt | 50.683843,4.79167 | Crop field |
| 5B-B3 | Incourt | 50.683843,4.79167 | Crop field |
| 5B-B4 | Incourt | 50.683843,4.79167 | Crop field |
| 5B-B5 | Incourt | 50.683843,4.79167 | Crop field |
| 5B-B6 | Incourt | 50.683843,4.79167 | Crop field |
| 5B-B7 | Incourt | 50.683843,4.79167 | Crop field |
| 5B-B8 | Incourt | 50.683843,4.79167 | Crop field |
| 5B-B9 | Incourt | 50.683843,4.79167 | Crop field |
| 6B-B1 | Incourt | 50.67135,4.81096 | Crop field |
| 6B-B2 | Incourt | 50.67135,4.81096 | Crop field |
| 6B-B3 | Incourt | 50.67135,4.81096 | Crop field |
| 6B-B4 | Incourt | 50.67135,4.81096 | Crop field |
| 6B-B5 | Incourt | 50.67135,4.81096 | Crop field |
| 6B-B6 | Incourt | 50.67135,4.81096 | Crop field |
| 6B-B7 | Incourt | 50.67135,4.81096 | Crop field |
| 6B-B8 | Incourt | 50.67135,4.81096 | Crop field |
| 6B-B9 | Incourt | 50.67135,4.81096 | Crop field |
| 7B-B1 | Gembloux | 50.572922,4.705045 | Crop field |
| 7B-B2 | Gembloux | 50.572922,4.705045 | Crop field |
| 7B-B3 | Gembloux | 50.572922,4.705045 | Crop field |
| 7B-B4 | Gembloux | 50.572922,4.705045 | Crop field |
| 7B-B5 | Gembloux | 50.572922,4.705045 | Crop field |
| 7B-B6 | Gembloux | 50.572922,4.705045 | Crop field |
| 7B-B7 | Gembloux | 50.572922,4.705045 | Crop field |
| 7B-B8 | Gembloux | 50.572922,4.705045 | Crop field |
| 7B-B9 | Gembloux | 50.572922,4.705045 | Crop field |
| 8B-B1 | Gembloux | 50.594653,4.732018 | Crop field |
| 8B-B2 | Gembloux | 50.594653,4.732018 | Crop field |
| 8B-B3 | Gembloux | 50.594653,4.732018 | Crop field |
| 8B-B4 | Gembloux | 50.594653,4.732018 | Crop field |
| 8B-B5 | Gembloux | 50.594653,4.732018 | Crop field |
| 8B-B6 | Gembloux | 50.594653,4.732018 | Crop field |
| 8B-B7 | Gembloux | 50.594653,4.732018 | Crop field |
| 8B-B8 | Gembloux | 50.594653,4.732018 | Crop field |
| 8B-B9 | Gembloux | 50.594653,4.732018 | Crop field |
| 9B-B1 | Gembloux | 50.600537,4.732275 | Crop field |
| 9B-B2 | Gembloux | 50.600537,4.732275 | Crop field |
| 9B-B3 | Gembloux | 50.600537,4.732275 | Crop field |
| 9B-B4 | Gembloux | 50.600537,4.732275 | Crop field |
| 9B-B5 | Gembloux | 50.600537,4.732275 | Crop field |
| 9B-B6 | Gembloux | 50.600537,4.732275 | Crop field |
| 9B-B7 | Gembloux | 50.600537,4.732275 | Crop field |
| 9B-B8 | Gembloux | 50.600537,4.732275 | Crop field |
| 9B-B9 | Gembloux | 50.600537,4.732275 | Crop field |
| 10B-B1 | Gembloux | 50.599829,4.733176 | Crop field |
| 10B-B2 | Gembloux | 50.599829,4.733176 | Crop field |
| 10B-B3 | Gembloux | 50.599829,4.733176 | Crop field |
| 10B-B4 | Gembloux | 50.599829,4.733176 | Crop field |
| 10B-B5 | Gembloux | 50.599829,4.733176 | Crop field |
| 10B-B6 | Gembloux | 50.599829,4.733176 | Crop field |
| 10B-B7 | Gembloux | 50.599829,4.733176 | Crop field |
| 10B-B8 | Gembloux | 50.599829,4.733176 | Crop field |
| 10B-B9 | Gembloux | 50.599829,4.733176 | Crop field |
| 11B-B1 | Gembloux | 50.569269,4.670348 | Crop field |
| 11B-B2 | Gembloux | 50.569269,4.670348 | Crop field |
| 11B-B3 | Gembloux | 50.569269,4.670348 | Crop field |
| 11B-B4 | Gembloux | 50.569269,4.670348 | Crop field |
| 11B-B5 | Gembloux | 50.569269,4.670348 | Crop field |
| 11B-B6 | Gembloux | 50.569269,4.670348 | Crop field |
| 11B-B7 | Gembloux | 50.569269,4.670348 | Crop field |
| 11B-B8 | Gembloux | 50.569269,4.670348 | Crop field |
| 11B-B9 | Gembloux | 50.569269,4.670348 | Crop field |
| 12B-B1 | Gembloux | 50.568969,4.671357 | Crop field |
| 12B-B2 | Gembloux | 50.568969,4.671357 | Crop field |
| 12B-B3 | Gembloux | 50.568969,4.671357 | Crop field |
| 12B-B4 | Gembloux | 50.568969,4.671357 | Crop field |
| 12B-B5 | Gembloux | 50.568969,4.671357 | Crop field |
| 12B-B6 | Gembloux | 50.568969,4.671357 | Crop field |
| 12B-B7 | Gembloux | 50.568969,4.671357 | Crop field |
| 12B-B8 | Gembloux | 50.568969,4.671357 | Crop field |
| 12B-B9 | Gembloux | 50.568969,4.671357 | Crop field |
| 13B-B1 | Gembloux | 50.601054,4.86866 | Crop field |
| 13B-B2 | Gembloux | 50.601054,4.86866 | Crop field |
| 13B-B3 | Gembloux | 50.601054,4.86866 | Crop field |
| 13B-B4 | Gembloux | 50.601054,4.86866 | Crop field |
| 13B-B5 | Gembloux | 50.601054,4.86866 | Crop field |
| 13B-B6 | Gembloux | 50.601054,4.86866 | Crop field |
| 13B-B7 | Gembloux | 50.601054,4.86866 | Crop field |
| 13B-B8 | Gembloux | 50.601054,4.86866 | Crop field |
| 13B-B9 | Gembloux | 50.601054,4.86866 | Crop field |
| 14B-B1 | Cognelée | 50.528304,4.899151 | Crop field |
| 14B-B2 | Cognelée | 50.528304,4.899151 | Crop field |
| 14B-B3 | Cognelée | 50.528304,4.899151 | Crop field |
| 14B-B4 | Cognelée | 50.528304,4.899151 | Crop field |
| 14B-B5 | Cognelée | 50.528304,4.899151 | Crop field |
| 14B-B6 | Cognelée | 50.528304,4.899151 | Crop field |
| 14B-B7 | Cognelée | 50.528304,4.899151 | Crop field |
| 14B-B8 | Cognelée | 50.528304,4.899151 | Crop field |
| 14B-B9 | Cognelée | 50.528304,4.899151 | Crop field |
| 15B-B1 | Bousval | 50.614672,4.494953 | Crop field |
| 15B-B2 | Bousval | 50.614672,4.494953 | Crop field |
| 15B-B3 | Bousval | 50.614672,4.494953 | Crop field |
| 15B-B4 | Bousval | 50.614672,4.494953 | Crop field |
| 15B-B5 | Bousval | 50.614672,4.494953 | Crop field |
| 15B-B6 | Bousval | 50.614672,4.494953 | Crop field |
| 15B-B7 | Bousval | 50.614672,4.494953 | Crop field |
| 15B-B8 | Bousval | 50.614672,4.494953 | Crop field |
| 15B-B9 | Bousval | 50.614672,4.494953 | Crop field |
| 16B-B1 | Court st Etienne | 50.632505,4.550829 | Crop field |
| 16B-B2 | Court st Etienne | 50.632505,4.550829 | Crop field |
| 16B-B3 | Court st Etienne | 50.632505,4.550829 | Crop field |
| 16B-B4 | Court st Etienne | 50.632505,4.550829 | Crop field |
| 16B-B5 | Court st Etienne | 50.632505,4.550829 | Crop field |
| 16B-B6 | Court st Etienne | 50.632505,4.550829 | Crop field |
| 16B-B7 | Court st Etienne | 50.632505,4.550829 | Crop field |
| 16B-B8 | Court st Etienne | 50.632505,4.550829 | Crop field |
| 16B-B9 | Court st Etienne | 50.632505,4.550829 | Crop field |
| 17B-B1 | Court st Etienne | 50.630001,4.551762 | Crop field |
| 17B-B2 | Court st Etienne | 50.630001,4.551762 | Crop field |
| 17B-B3 | Court st Etienne | 50.630001,4.551762 | Crop field |
| 17B-B4 | Court st Etienne | 50.630001,4.551762 | Crop field |
| 17B-B5 | Court st Etienne | 50.630001,4.551762 | Crop field |
| 17B-B6 | Court st Etienne | 50.630001,4.551762 | Crop field |
| 17B-B7 | Court st Etienne | 50.630001,4.551762 | Crop field |
| 17B-B8 | Court st Etienne | 50.630001,4.551762 | Crop field |
| 17B-B9 | Court st Etienne | 50.630001,4.551762 | Crop field |
| 18B-B1 | Louvain-la-Neuve | 50.675793,4.624686 | Crop field |
| 18B-B2 | Louvain-la-Neuve | 50.675793,4.624686 | Crop field |
| 18B-B3 | Louvain-la-Neuve | 50.675793,4.624686 | Crop field |
| 18B-B4 | Louvain-la-Neuve | 50.675793,4.624686 | Crop field |
| 18B-B5 | Louvain-la-Neuve | 50.675793,4.624686 | Crop field |
| 18B-B6 | Louvain-la-Neuve | 50.675793,4.624686 | Crop field |
| 18B-B7 | Louvain-la-Neuve | 50.675793,4.624686 | Crop field |
| 18B-B8 | Louvain-la-Neuve | 50.675793,4.624686 | Crop field |
| 18B-B9 | Louvain-la-Neuve | 50.675793,4.624686 | Crop field |
| 19B-B1 | Coxyde | 51.104452,2.660258 | Crop field |
| 19B-B2 | Coxyde | 51.104452,2.660258 | Crop field |
| 19B-B3 | Coxyde | 51.104452,2.660258 | Crop field |
| 19B-B4 | Coxyde | 51.104452,2.660258 | Crop field |
| 19B-B5 | Coxyde | 51.104452,2.660258 | Crop field |
| 19B-B6 | Coxyde | 51.104452,2.660258 | Crop field |
| 19B-B7 | Coxyde | 51.104452,2.660258 | Crop field |
| 19B-B8 | Coxyde | 51.104452,2.660258 | Crop field |
| 19B-B9 | Coxyde | 51.104452,2.660258 | Crop field |
| 20B-B1 | Coxyde | 51.089204,2.636547 | Crop field |
| 20B-B2 | Coxyde | 51.089204,2.636547 | Crop field |
| 20B-B3 | Coxyde | 51.089204,2.636547 | Crop field |
| 20B-B4 | Coxyde | 51.089204,2.636547 | Crop field |
| 20B-B5 | Coxyde | 51.089204,2.636547 | Crop field |
| 20B-B6 | Coxyde | 51.089204,2.636547 | Crop field |
| 20B-B7 | Coxyde | 51.089204,2.636547 | Crop field |
| 20B-B8 | Coxyde | 51.089204,2.636547 | Crop field |
| 20B-B9 | Coxyde | 51.089204,2.636547 | Crop field |
| 21B-B1 | Coxyde | 51.090073,2.636118 | Crop field |
| 21B-B2 | Coxyde | 51.090073,2.636118 | Crop field |
| 21B-B3 | Coxyde | 51.090073,2.636118 | Crop field |
| 21B-B4 | Coxyde | 51.090073,2.636118 | Crop field |
| 21B-B5 | Coxyde | 51.090073,2.636118 | Crop field |
| 21B-B6 | Coxyde | 51.090073,2.636118 | Crop field |
| 21B-B7 | Coxyde | 51.090073,2.636118 | Crop field |
| 21B-B8 | Coxyde | 51.090073,2.636118 | Crop field |
| 21B-B9 | Coxyde | 51.090073,2.636118 | Crop field |
| 22B-B1 | Furne | 51.059489,2.663519 | Crop field |
| 22B-B2 | Furne | 51.059489,2.663519 | Crop field |
| 22B-B3 | Furne | 51.059489,2.663519 | Crop field |
| 22B-B4 | Furne | 51.059489,2.663519 | Crop field |
| 22B-B5 | Furne | 51.059489,2.663519 | Crop field |
| 22B-B6 | Furne | 51.059489,2.663519 | Crop field |
| 22B-B7 | Furne | 51.059489,2.663519 | Crop field |
| 22B-B8 | Furne | 51.059489,2.663519 | Crop field |
| 22B-B9 | Furne | 51.059489,2.663519 | Crop field |
| 23B-B1 | Furne | 51.070284,2.7088 | Crop field |
| 23B-B2 | Furne | 51.070284,2.7088 | Crop field |
| 23B-B3 | Furne | 51.070284,2.7088 | Crop field |
| 23B-B4 | Furne | 51.070284,2.7088 | Crop field |
| 23B-B5 | Furne | 51.070284,2.7088 | Crop field |
| 23B-B6 | Furne | 51.070284,2.7088 | Crop field |
| 23B-B7 | Furne | 51.070284,2.7088 | Crop field |
| 23B-B8 | Furne | 51.070284,2.7088 | Crop field |
| 23B-B9 | Furne | 51.070284,2.7088 | Crop field |
| 24B-B1 | Furne | 51.070122,2.709353 | Crop field |
| 24B-B2 | Furne | 51.070122,2.709353 | Crop field |
| 24B-B3 | Furne | 51.070122,2.709353 | Crop field |
| 24B-B4 | Furne | 51.070122,2.709353 | Crop field |
| 24B-B5 | Furne | 51.070122,2.709353 | Crop field |
| 24B-B6 | Furne | 51.070122,2.709353 | Crop field |
| 24B-B7 | Furne | 51.070122,2.709353 | Crop field |
| 24B-B8 | Furne | 51.070122,2.709353 | Crop field |
| 24B-B9 | Furne | 51.070122,2.709353 | Crop field |
| 25B-B1 | Furne | 51.063488,2.767482 | Crop field |
| 25B-B2 | Furne | 51.063488,2.767482 | Crop field |
| 25B-B3 | Furne | 51.063488,2.767482 | Crop field |
| 25B-B4 | Furne | 51.063488,2.767482 | Crop field |
| 25B-B5 | Furne | 51.063488,2.767482 | Crop field |
| 25B-B6 | Furne | 51.063488,2.767482 | Crop field |
| 25B-B7 | Furne | 51.063488,2.767482 | Crop field |
| 25B-B8 | Furne | 51.063488,2.767482 | Crop field |
| 25B-B9 | Furne | 51.063488,2.767482 | Crop field |
| 26B-B1 | Furne | 51.06338,2.768469 | Crop field |
| 26B-B2 | Furne | 51.06338,2.768469 | Crop field |
| 26B-B3 | Furne | 51.06338,2.768469 | Crop field |
| 26B-B4 | Furne | 51.06338,2.768469 | Crop field |
| 26B-B5 | Furne | 51.06338,2.768469 | Crop field |
| 26B-B6 | Furne | 51.06338,2.768469 | Crop field |
| 26B-B7 | Furne | 51.06338,2.768469 | Crop field |
| 26B-B8 | Furne | 51.06338,2.768469 | Crop field |
| 26B-B9 | Furne | 51.06338,2.768469 | Crop field |
| 27B-B1 | Waremme | n/a | Crop field |
| 27B-B2 | Waremme | n/a | Crop field |
| 27B-B3 | Waremme | n/a | Crop field |
| 27B-B4 | Waremme | n/a | Crop field |
| 27B-B5 | Waremme | n/a | Crop field |
| 27B-B6 | Waremme | n/a | Crop field |
| 27B-B7 | Waremme | n/a | Crop field |
| 27B-B8 | Waremme | n/a | Crop field |
| 27B-B9 | Waremme | n/a | Crop field |
| 28B-B1 | Waremme | n/a | Crop field |
| 28B-B2 | Waremme | n/a | Crop field |
| 28B-B3 | Waremme | n/a | Crop field |
| 28B-B4 | Waremme | n/a | Crop field |
| 28B-B5 | Waremme | n/a | Crop field |
| 28B-B6 | Waremme | n/a | Crop field |
| 28B-B7 | Waremme | n/a | Crop field |
| 28B-B8 | Waremme | n/a | Crop field |
| 28B-B9 | Waremme | n/a | Crop field |
| 29B-B1 | Waremme | n/a | Crop field |
| 29B-B2 | Waremme | n/a | Crop field |
| 29B-B3 | Waremme | n/a | Crop field |
| 29B-B4 | Waremme | n/a | Crop field |
| 29B-B5 | Waremme | n/a | Crop field |
| 29B-B6 | Waremme | n/a | Crop field |
| 29B-B7 | Waremme | n/a | Crop field |
| 29B-B8 | Waremme | n/a | Crop field |
| 29B-B9 | Waremme | n/a | Crop field |
| 30B-B1 | Waremme | n/a | Crop field |
| 30B-B2 | Waremme | n/a | Crop field |
| 30B-B3 | Waremme | n/a | Crop field |
| 30B-B4 | Waremme | n/a | Crop field |
| 30B-B5 | Waremme | n/a | Crop field |
| 30B-B6 | Waremme | n/a | Crop field |
| 30B-B7 | Waremme | n/a | Crop field |
| 30B-B8 | Waremme | n/a | Crop field |
| 30B-B9 | Waremme | n/a | Crop field |
| 31B-B1 | Soumagne | 50.652113,5.712376 | Crop field |
| 31B-B2 | Soumagne | 50.652113,5.712376 | Crop field |
| 31B-B3 | Soumagne | 50.652113,5.712376 | Crop field |
| 31B-B4 | Soumagne | 50.652113,5.712376 | Crop field |
| 31B-B5 | Soumagne | 50.652113,5.712376 | Crop field |
| 31B-B6 | Soumagne | 50.652113,5.712376 | Crop field |
| 31B-B7 | Soumagne | 50.652113,5.712376 | Crop field |
| 31B-B8 | Soumagne | 50.652113,5.712376 | Crop field |
| 31B-B9 | Soumagne | 50.652113,5.712376 | Crop field |
| 32B-B1 | Soumagne | 50.632832,5.703814 | Crop field |
| 32B-B2 | Soumagne | 50.632832,5.703814 | Crop field |
| 32B-B3 | Soumagne | 50.632832,5.703814 | Crop field |
| 32B-B4 | Soumagne | 50.632832,5.703814 | Crop field |
| 32B-B5 | Soumagne | 50.632832,5.703814 | Crop field |
| 32B-B6 | Soumagne | 50.632832,5.703814 | Crop field |
| 32B-B7 | Soumagne | 50.632832,5.703814 | Crop field |
| 32B-B8 | Soumagne | 50.632832,5.703814 | Crop field |
| 32B-B9 | Soumagne | 50.632832,5.703814 | Crop field |
| 33B-B1 | Soumagne | 50.663935,5.697779 | Crop field |
| 33B-B2 | Soumagne | 50.663935,5.697779 | Crop field |
| 33B-B3 | Soumagne | 50.663935,5.697779 | Crop field |
| 33B-B4 | Soumagne | 50.663935,5.697779 | Crop field |
| 33B-B5 | Soumagne | 50.663935,5.697779 | Crop field |
| 33B-B6 | Soumagne | 50.663935,5.697779 | Crop field |
| 33B-B7 | Soumagne | 50.663935,5.697779 | Crop field |
| 33B-B8 | Soumagne | 50.663935,5.697779 | Crop field |
| 33B-B9 | Soumagne | 50.663935,5.697779 | Crop field |
| 34B-B1 | Soumagne | 50.661326,5.673505 | Crop field |
| 34B-B2 | Soumagne | 50.661326,5.673505 | Crop field |
| 34B-B3 | Soumagne | 50.661326,5.673505 | Crop field |
| 34B-B4 | Soumagne | 50.661326,5.673505 | Crop field |
| 34B-B5 | Soumagne | 50.661326,5.673505 | Crop field |
| 34B-B6 | Soumagne | 50.661326,5.673505 | Crop field |
| 34B-B7 | Soumagne | 50.661326,5.673505 | Crop field |
| 34B-B8 | Soumagne | 50.661326,5.673505 | Crop field |
| 34B-B9 | Soumagne | 50.661326,5.673505 | Crop field |
| 35B-B1 | Soumagne | 50.660867,5.674632 | Crop field |
| 35B-B2 | Soumagne | 50.660867,5.674632 | Crop field |
| 35B-B3 | Soumagne | 50.660867,5.674632 | Crop field |
| 35B-B4 | Soumagne | 50.660867,5.674632 | Crop field |
| 35B-B5 | Soumagne | 50.660867,5.674632 | Crop field |
| 35B-B6 | Soumagne | 50.660867,5.674632 | Crop field |
| 35B-B7 | Soumagne | 50.660867,5.674632 | Crop field |
| 35B-B8 | Soumagne | 50.660867,5.674632 | Crop field |
| 35B-B9 | Soumagne | 50.660867,5.674632 | Crop field |
| 41B-B1 | Bertrix | 49.848617,5.239266 | Crop field |
| 41B-B2 | Bertrix | 49.848617,5.239266 | Crop field |
| 41B-B3 | Bertrix | 49.848617,5.239266 | Crop field |
| 41B-B4 | Bertrix | 49.848617,5.239266 | Crop field |
| 41B-B5 | Bertrix | 49.848617,5.239266 | Crop field |
| 41B-B6 | Bertrix | 49.848617,5.239266 | Crop field |
| 41B-B7 | Bertrix | 49.848617,5.239266 | Crop field |
| 41B-B8 | Bertrix | 49.848617,5.239266 | Crop field |
| 41B-B9 | Bertrix | 49.848617,5.239266 | Crop field |
| 42B-B1 | Bertrix | 49.85128,5.25257 | Crop field |
| 42B-B2 | Bertrix | 49.85128,5.25257 | Crop field |
| 42B-B3 | Bertrix | 49.85128,5.25257 | Crop field |
| 42B-B4 | Bertrix | 49.85128,5.25257 | Crop field |
| 42B-B5 | Bertrix | 49.85128,5.25257 | Crop field |
| 42B-B6 | Bertrix | 49.85128,5.25257 | Crop field |
| 42B-B7 | Bertrix | 49.85128,5.25257 | Crop field |
| 42B-B8 | Bertrix | 49.85128,5.25257 | Crop field |
| 42B-B9 | Bertrix | 49.85128,5.25257 | Crop field |
| 43B-B1 | Bertrix | 49.85128,5.25257 | Crop field |
| 43B-B2 | Bertrix | 49.85128,5.25257 | Crop field |
| 43B-B3 | Bertrix | 49.85128,5.25257 | Crop field |
| 43B-B4 | Bertrix | 49.85128,5.25257 | Crop field |
| 43B-B5 | Bertrix | 49.85128,5.25257 | Crop field |
| 43B-B6 | Bertrix | 49.85128,5.25257 | Crop field |
| 43B-B7 | Bertrix | 49.85128,5.25257 | Crop field |
| 43B-B8 | Bertrix | 49.85128,5.25257 | Crop field |
| 43B-B9 | Bertrix | 49.85128,5.25257 | Crop field |
| 44B-B1 | Bertrix | 49.84403,5.265884 | Crop field |
| 44B-B2 | Bertrix | 49.84403,5.265884 | Crop field |
| 44B-B3 | Bertrix | 49.84403,5.265884 | Crop field |
| 44B-B4 | Bertrix | 49.84403,5.265884 | Crop field |
| 44B-B5 | Bertrix | 49.84403,5.265884 | Crop field |
| 44B-B6 | Bertrix | 49.84403,5.265884 | Crop field |
| 44B-B7 | Bertrix | 49.84403,5.265884 | Crop field |
| 44B-B8 | Bertrix | 49.84403,5.265884 | Crop field |
| 44B-B9 | Bertrix | 49.84403,5.265884 | Crop field |
| 45B-B1 | Bertrix | 49.782615,5.213131 | Crop field |
| 45B-B2 | Bertrix | 49.782615,5.213131 | Crop field |
| 45B-B3 | Bertrix | 49.782615,5.213131 | Crop field |
| 45B-B4 | Bertrix | 49.782615,5.213131 | Crop field |
| 45B-B5 | Bertrix | 49.782615,5.213131 | Crop field |
| 45B-B6 | Bertrix | 49.782615,5.213131 | Crop field |
| 45B-B7 | Bertrix | 49.782615,5.213131 | Crop field |
| 45B-B8 | Bertrix | 49.782615,5.213131 | Crop field |
| 45B-B9 | Bertrix | 49.782615,5.213131 | Crop field |
| 46B-B1 | Ath | 50.645821,3.856544 | Crop field |
| 46B-B2 | Ath | 50.645821,3.856544 | Crop field |
| 46B-B3 | Ath | 50.645821,3.856544 | Crop field |
| 46B-B4 | Ath | 50.645821,3.856544 | Crop field |
| 46B-B5 | Ath | 50.645821,3.856544 | Crop field |
| 46B-B6 | Ath | 50.645821,3.856544 | Crop field |
| 46B-B7 | Ath | 50.645821,3.856544 | Crop field |
| 46B-B8 | Ath | 50.645821,3.856544 | Crop field |
| 46B-B9 | Ath | 50.645821,3.856544 | Crop field |
| 47B-B1 | Lens | 50.586615,3.906841 | Crop field |
| 47B-B2 | Lens | 50.586615,3.906841 | Crop field |
| 47B-B3 | Lens | 50.586615,3.906841 | Crop field |
| 47B-B4 | Lens | 50.586615,3.906841 | Crop field |
| 47B-B5 | Lens | 50.586615,3.906841 | Crop field |
| 47B-B6 | Lens | 50.586615,3.906841 | Crop field |
| 47B-B7 | Lens | 50.586615,3.906841 | Crop field |
| 47B-B8 | Lens | 50.586615,3.906841 | Crop field |
| 47B-B9 | Lens | 50.586615,3.906841 | Crop field |
| 48B-B1 | Soignies | 50.563978,4.017375 | Crop field |
| 48B-B2 | Soignies | 50.563978,4.017375 | Crop field |
| 48B-B3 | Soignies | 50.563978,4.017375 | Crop field |
| 48B-B4 | Soignies | 50.563978,4.017375 | Crop field |
| 48B-B5 | Soignies | 50.563978,4.017375 | Crop field |
| 48B-B6 | Soignies | 50.563978,4.017375 | Crop field |
| 48B-B7 | Soignies | 50.563978,4.017375 | Crop field |
| 48B-B8 | Soignies | 50.563978,4.017375 | Crop field |
| 48B-B9 | Soignies | 50.563978,4.017375 | Crop field |
| 49B-B1 | Chièvres | 50.550877,3.825468 | Crop field |
| 49B-B2 | Chièvres | 50.550877,3.825468 | Crop field |
| 49B-B3 | Chièvres | 50.550877,3.825468 | Crop field |
| 49B-B4 | Chièvres | 50.550877,3.825468 | Crop field |
| 49B-B5 | Chièvres | 50.550877,3.825468 | Crop field |
| 49B-B6 | Chièvres | 50.550877,3.825468 | Crop field |
| 49B-B7 | Chièvres | 50.550877,3.825468 | Crop field |
| 49B-B8 | Chièvres | 50.550877,3.825468 | Crop field |
| 49B-B9 | Chièvres | 50.550877,3.825468 | Crop field |
| 50B-B1 | Ath | 50.595933,3.710418 | Crop field |
| 50B-B2 | Ath | 50.595933,3.710418 | Crop field |
| 50B-B3 | Ath | 50.595933,3.710418 | Crop field |
| 50B-B4 | Ath | 50.595933,3.710418 | Crop field |
| 50B-B5 | Ath | 50.595933,3.710418 | Crop field |
| 50B-B6 | Ath | 50.595933,3.710418 | Crop field |
| 50B-B7 | Ath | 50.595933,3.710418 | Crop field |
| 50B-B8 | Ath | 50.595933,3.710418 | Crop field |
| 50B-B9 | Ath | 50.595933,3.710418 | Crop field |
| 51B-B1 | Habay | 49.737759,5.616503 | Crop field |
| 51B-B2 | Habay | 49.737759,5.616503 | Crop field |
| 51B-B3 | Habay | 49.737759,5.616503 | Crop field |
| 51B-B4 | Habay | 49.737759,5.616503 | Crop field |
| 51B-B5 | Habay | 49.737759,5.616503 | Crop field |
| 51B-B6 | Habay | 49.737759,5.616503 | Crop field |
| 51B-B7 | Habay | 49.737759,5.616503 | Crop field |
| 51B-B8 | Habay | 49.737759,5.616503 | Crop field |
| 51B-B9 | Habay | 49.737759,5.616503 | Crop field |
| 52B-B1 | Habay | 49.737829,5.615934 | Crop field |
| 52B-B2 | Habay | 49.737829,5.615934 | Crop field |
| 52B-B3 | Habay | 49.737829,5.615934 | Crop field |
| 52B-B4 | Habay | 49.737829,5.615934 | Crop field |
| 52B-B5 | Habay | 49.737829,5.615934 | Crop field |
| 52B-B6 | Habay | 49.737829,5.615934 | Crop field |
| 52B-B7 | Habay | 49.737829,5.615934 | Crop field |
| 52B-B8 | Habay | 49.737829,5.615934 | Crop field |
| 52B-B9 | Habay | 49.737829,5.615934 | Crop field |
| 53B-B1 | Habay | 49.724403,5.601751 | Crop field |
| 53B-B2 | Habay | 49.724403,5.601751 | Crop field |
| 53B-B3 | Habay | 49.724403,5.601751 | Crop field |
| 53B-B4 | Habay | 49.724403,5.601751 | Crop field |
| 53B-B5 | Habay | 49.724403,5.601751 | Crop field |
| 53B-B6 | Habay | 49.724403,5.601751 | Crop field |
| 53B-B7 | Habay | 49.724403,5.601751 | Crop field |
| 53B-B8 | Habay | 49.724403,5.601751 | Crop field |
| 53B-B9 | Habay | 49.724403,5.601751 | Crop field |
| C1-B1 | Rebecq | n/a | Compost |
| C1-B2 | Rebecq | n/a | Compost |
| C1-B3 | Rebecq | n/a | Compost |
| C1-B4 | Rebecq | n/a | Compost |
| C1-B5 | Rebecq | n/a | Compost |
| C1-B6 | Rebecq | n/a | Compost |
| C1-B7 | Rebecq | n/a | Compost |
| C1-B8 | Rebecq | n/a | Compost |
| C1-B9 | Rebecq | n/a | Compost |
| C2-B1 | Bousval | n/a | Compost |
| C2-B2 | Bousval | n/a | Compost |
| C2-B3 | Bousval | n/a | Compost |
| C2-B4 | Bousval | n/a | Compost |
| C2-B5 | Bousval | n/a | Compost |
| C2-B6 | Bousval | n/a | Compost |
| C2-B7 | Bousval | n/a | Compost |
| C2-B8 | Bousval | n/a | Compost |
| C2-B9 | Bousval | n/a | Compost |
| C3-B1 | Ath | 51.076667, 4.427567 | Compost |
| C3-B2 | Ath | 51.076667, 4.427567 | Compost |
| C3-B3 | Ath | 51.076667, 4.427567 | Compost |
| C3-B4 | Ath | 51.076667, 4.427567 | Compost |
| C3-B5 | Ath | 51.076667, 4.427567 | Compost |
| C3-B6 | Ath | 51.076667, 4.427567 | Compost |
| C3-B7 | Ath | 51.076667, 4.427567 | Compost |
| C3-B8 | Ath | 51.076667, 4.427567 | Compost |
| C3-B9 | Ath | 51.076667, 4.427567 | Compost |
| C4-B1 | Habay | 49,73745, 5.61766 | Compost |
| C4-B2 | Habay | 49,73745, 5.61766 | Compost |
| C4-B3 | Habay | 49,73745, 5.61766 | Compost |
| C4-B4 | Habay | 49,73745, 5.61766 | Compost |
| C4-B5 | Habay | 49,73745, 5.61766 | Compost |
| C4-B6 | Habay | 49,73745, 5.61766 | Compost |
| C4-B7 | Habay | 49,73745, 5.61766 | Compost |
| C4-B8 | Habay | 49,73745, 5.61766 | Compost |
| C4-B9 | Habay | 49,73745, 5.61766 | Compost |
| F1-B1 | Rebecq | 50.647229,4.15516 | Manure |
| F1-B2 | Rebecq | 50.647229,4.15516 | Manure |
| F1-B3 | Rebecq | 50.647229,4.15516 | Manure |
| F1-B4 | Rebecq | 50.647229,4.15516 | Manure |
| F1-B5 | Rebecq | 50.647229,4.15516 | Manure |
| F1-B6 | Rebecq | 50.647229,4.15516 | Manure |
| F1-B7 | Rebecq | 50.647229,4.15516 | Manure |
| F1-B8 | Rebecq | 50.647229,4.15516 | Manure |
| F1-B9 | Rebecq | 50.647229,4.15516 | Manure |
| F2-B1 | Bertrix | 49.846932,5.238757 | Manure |
| F2-B2 | Bertrix | 49.846932,5.238757 | Manure |
| F2-B3 | Bertrix | 49.846932,5.238757 | Manure |
| F2-B4 | Bertrix | 49.846932,5.238757 | Manure |
| F2-B5 | Bertrix | 49.846932,5.238757 | Manure |
| F2-B6 | Bertrix | 49.846932,5.238757 | Manure |
| F2-B7 | Bertrix | 49.846932,5.238757 | Manure |
| F2-B8 | Bertrix | 49.846932,5.238757 | Manure |
| F2-B9 | Bertrix | 49.846932,5.238757 | Manure |
| F3-B1 | Bertrix | 49.846937,5.238751 | Manure |
| F3-B2 | Bertrix | 49.846937,5.238751 | Manure |
| F3-B3 | Bertrix | 49.846937,5.238751 | Manure |
| F3-B4 | Bertrix | 49.846937,5.238751 | Manure |
| F3-B5 | Bertrix | 49.846937,5.238751 | Manure |
| F3-B6 | Bertrix | 49.846937,5.238751 | Manure |
| F3-B7 | Bertrix | 49.846937,5.238751 | Manure |
| F3-B8 | Bertrix | 49.846937,5.238751 | Manure |
| F3-B9 | Bertrix | 49.846937,5.238751 | Manure |
| F4-B1 | Bertrix | 49.844075,5.265056 | Manure |
| F4-B2 | Bertrix | 49.844075,5.265056 | Manure |
| F4-B3 | Bertrix | 49.844075,5.265056 | Manure |
| F4-B4 | Bertrix | 49.844075,5.265056 | Manure |
| F4-B5 | Bertrix | 49.844075,5.265056 | Manure |
| F4-B6 | Bertrix | 49.844075,5.265056 | Manure |
| F4-B7 | Bertrix | 49.844075,5.265056 | Manure |
| F4-B8 | Bertrix | 49.844075,5.265056 | Manure |
| F4-B9 | Bertrix | 49.844075,5.265056 | Manure |
| F5-B1 | Bertrix | 49.782636,5.21363 | Manure |
| F5-B2 | Bertrix | 49.782636,5.21363 | Manure |
| F5-B3 | Bertrix | 49.782636,5.21363 | Manure |
| F5-B4 | Bertrix | 49.782636,5.21363 | Manure |
| F5-B5 | Bertrix | 49.782636,5.21363 | Manure |
| F5-B6 | Bertrix | 49.782636,5.21363 | Manure |
| F5-B7 | Bertrix | 49.782636,5.21363 | Manure |
| F5-B8 | Bertrix | 49.782636,5.21363 | Manure |
| F5-B9 | Bertrix | 49.782636,5.21363 | Manure |
| F6-B1 | Soignies | 50.56837, 4.01665 | Manure |
| F6-B2 | Soignies | 50.56837, 4.01665 | Manure |
| F6-B3 | Soignies | 50.56837, 4.01665 | Manure |
| F6-B4 | Soignies | 50.56837, 4.01665 | Manure |
| F6-B5 | Soignies | 50.56837, 4.01665 | Manure |
| F6-B6 | Soignies | 50.56837, 4.01665 | Manure |
| F6-B7 | Soignies | 50.56837, 4.01665 | Manure |
| F6-B8 | Soignies | 50.56837, 4.01665 | Manure |
| F6-B9 | Soignies | 50.56837, 4.01665 | Manure |
| F7-B1 | Brugelette | 50.59957, 3.839467 | Manure |
| F7-B2 | Brugelette | 50.59957, 3.839467 | Manure |
| F7-B3 | Brugelette | 50.59957, 3.839467 | Manure |
| F7-B4 | Brugelette | 50.59957, 3.839467 | Manure |
| F7-B5 | Brugelette | 50.59957, 3.839467 | Manure |
| F7-B6 | Brugelette | 50.59957, 3.839467 | Manure |
| F7-B7 | Brugelette | 50.59957, 3.839467 | Manure |
| F7-B8 | Brugelette | 50.59957, 3.839467 | Manure |
| F7-B9 | Brugelette | 50.59957, 3.839467 | Manure |
| F8-B1 | Habay | 49.739067, 5.615467 | Manure |
| F8-B2 | Habay | 49.739067, 5.615467 | Manure |
| F8-B3 | Habay | 49.739067, 5.615467 | Manure |
| F8-B4 | Habay | 49.739067, 5.615467 | Manure |
| F8-B5 | Habay | 49.739067, 5.615467 | Manure |
| F8-B6 | Habay | 49.739067, 5.615467 | Manure |
| F8-B7 | Habay | 49.739067, 5.615467 | Manure |
| F8-B8 | Habay | 49.739067, 5.615467 | Manure |
| F8-B9 | Habay | 49.739067, 5.615467 | Manure |
| 41R-B1 | Bertrix | 4.95054348, 5.142126 | Potato root |
| 41R-B2 | Bertrix | 4.95054348, 5.142126 | Potato root |
| 41R-B3 | Bertrix | 4.95054348, 5.142126 | Potato root |
| 41R-B4 | Bertrix | 4.95054348, 5.142126 | Potato root |
| 41R-B5 | Bertrix | 4.95054348, 5.142126 | Potato root |
| 41R-B6 | Bertrix | 4.95054348, 5.142126 | Potato root |
| 41R-B7 | Bertrix | 4.95054348, 5.142126 | Potato root |
| 41R-B8 | Bertrix | 4.95054348, 5.142126 | Potato root |
| 41R-B9 | Bertrix | 4.95054348, 5.142126 | Potato root |
| 41L-B1 | Bertrix | 4.95054348, 5.142126 | Potato leaf |
| 41L-B2 | Bertrix | 4.95054348, 5.142126 | Potato leaf |
| 41L-B3 | Bertrix | 4.95054348, 5.142126 | Potato leaf |
| 41L-B4 | Bertrix | 4.95054348, 5.142126 | Potato leaf |
| 41L-B5 | Bertrix | 4.95054348, 5.142126 | Potato leaf |
| 41L-B6 | Bertrix | 4.95054348, 5.142126 | Potato leaf |
| 41L-B7 | Bertrix | 4.95054348, 5.142126 | Potato leaf |
| 41L-B8 | Bertrix | 4.95054348, 5.142126 | Potato leaf |
| 41L-B9 | Bertrix | 4.95054348, 5.142126 | Potato leaf |
| 41T-B1 | Bertrix | 4.95054348, 5.142126 | Potato tuber |
| 41T-B2 | Bertrix | 4.95054348, 5.142126 | Potato tuber |
| 41T-B3 | Bertrix | 4.95054348, 5.142126 | Potato tuber |
| 41T-B4 | Bertrix | 4.95054348, 5.142126 | Potato tuber |
| 41T-B5 | Bertrix | 4.95054348, 5.142126 | Potato tuber |
| 41T-B6 | Bertrix | 4.95054348, 5.142126 | Potato tuber |
| 41T-B7 | Bertrix | 4.95054348, 5.142126 | Potato tuber |
| 41T-B8 | Bertrix | 4.95054348, 5.142126 | Potato tuber |
| 41T-B9 | Bertrix | 4.95054348, 5.142126 | Potato tuber |
| 42R-B1 | Bertrix | 49.514313,5.159788 | Potato root |
| 42R-B2 | Bertrix | 49.514313,5.159788 | Potato root |
| 42R-B3 | Bertrix | 49.514313,5.159788 | Potato root |
| 42R-B4 | Bertrix | 49.514313,5.159788 | Potato root |
| 42R-B5 | Bertrix | 49.514313,5.159788 | Potato root |
| 42R-B6 | Bertrix | 49.514313,5.159788 | Potato root |
| 42R-B7 | Bertrix | 49.514313,5.159788 | Potato root |
| 42R-B8 | Bertrix | 49.514313,5.159788 | Potato root |
| 42R-B9 | Bertrix | 49.514313,5.159788 | Potato root |
| 42L-B1 | Bertrix | 49.514313,5.159788 | Potato leaf |
| 42L-B2 | Bertrix | 49.514313,5.159788 | Potato leaf |
| 42L-B3 | Bertrix | 49.514313,5.159788 | Potato leaf |
| 42L-B4 | Bertrix | 49.514313,5.159788 | Potato leaf |
| 42L-B5 | Bertrix | 49.514313,5.159788 | Potato leaf |
| 42L-B6 | Bertrix | 49.514313,5.159788 | Potato leaf |
| 42L-B7 | Bertrix | 49.514313,5.159788 | Potato leaf |
| 42L-B8 | Bertrix | 49.514313,5.159788 | Potato leaf |
| 42L-B9 | Bertrix | 49.514313,5.159788 | Potato leaf |
| 42T-B1 | Bertrix | 49.514313,5.159788 | Potato tuber |
| 42T-B2 | Bertrix | 49.514313,5.159788 | Potato tuber |
| 42T-B3 | Bertrix | 49.514313,5.159788 | Potato tuber |
| 42T-B4 | Bertrix | 49.514313,5.159788 | Potato tuber |
| 42T-B5 | Bertrix | 49.514313,5.159788 | Potato tuber |
| 42T-B6 | Bertrix | 49.514313,5.159788 | Potato tuber |
| 42T-B7 | Bertrix | 49.514313,5.159788 | Potato tuber |
| 42T-B8 | Bertrix | 49.514313,5.159788 | Potato tuber |
| 42T-B9 | Bertrix | 49.514313,5.159788 | Potato tuber |
| 43R-B3 | Bertrix | 49.514313,5.159788 | Potato root |
| 43R-B4 | Bertrix | 49.514313,5.159788 | Potato root |
| 43R-B3 | Bertrix | 49.514313,5.159788 | Potato root |
| 43R-B4 | Bertrix | 49.514313,5.159788 | Potato root |
| 43R-B5 | Bertrix | 49.514313,5.159788 | Potato root |
| 43R-B6 | Bertrix | 49.514313,5.159788 | Potato root |
| 43R-B7 | Bertrix | 49.514313,5.159788 | Potato root |
| 43R-B8 | Bertrix | 49.514313,5.159788 | Potato root |
| 43R-B9 | Bertrix | 49.514313,5.159788 | Potato root |
| 43L-B3 | Bertrix | 49.514313,5.159788 | Potato leaf |
| 43L-B4 | Bertrix | 49.514313,5.159788 | Potato leaf |
| 43L-B3 | Bertrix | 49.514313,5.159788 | Potato leaf |
| 43L-B4 | Bertrix | 49.514313,5.159788 | Potato leaf |
| 43L-B5 | Bertrix | 49.514313,5.159788 | Potato leaf |
| 43L-B6 | Bertrix | 49.514313,5.159788 | Potato leaf |
| 43L-B7 | Bertrix | 49.514313,5.159788 | Potato leaf |
| 43L-B8 | Bertrix | 49.514313,5.159788 | Potato leaf |
| 43L-B9 | Bertrix | 49.514313,5.159788 | Potato leaf |
| 43T-B3 | Bertrix | 49.514313,5.159788 | Potato tuber |
| 43T-B4 | Bertrix | 49.514313,5.159788 | Potato tuber |
| 43T-B3 | Bertrix | 49.514313,5.159788 | Potato tuber |
| 43T-B4 | Bertrix | 49.514313,5.159788 | Potato tuber |
| 43T-B5 | Bertrix | 49.514313,5.159788 | Potato tuber |
| 43T-B6 | Bertrix | 49.514313,5.159788 | Potato tuber |
| 43T-B7 | Bertrix | 49.514313,5.159788 | Potato tuber |
| 43T-B8 | Bertrix | 49.514313,5.159788 | Potato tuber |
| 43T-B9 | Bertrix | 49.514313,5.159788 | Potato tuber |
| 44R-B3 | Bertrix | 49.5037597,5.1557164 | Potato root |
| 44R-B4 | Bertrix | 49.5037597,5.1557164 | Potato root |
| 44R-B3 | Bertrix | 49.5037597,5.1557164 | Potato root |
| 44R-B4 | Bertrix | 49.5037597,5.1557164 | Potato root |
| 44R-B5 | Bertrix | 49.5037597,5.1557164 | Potato root |
| 44R-B6 | Bertrix | 49.5037597,5.1557164 | Potato root |
| 44R-B7 | Bertrix | 49.5037597,5.1557164 | Potato root |
| 44R-B8 | Bertrix | 49.5037597,5.1557164 | Potato root |
| 44R-B9 | Bertrix | 49.5037597,5.1557164 | Potato root |
| 44L-B3 | Bertrix | 49.5037597,5.1557164 | Potato leaf |
| 44L-B4 | Bertrix | 49.5037597,5.1557164 | Potato leaf |
| 44L-B3 | Bertrix | 49.5037597,5.1557164 | Potato leaf |
| 44L-B4 | Bertrix | 49.5037597,5.1557164 | Potato leaf |
| 44L-B5 | Bertrix | 49.5037597,5.1557164 | Potato leaf |
| 44L-B6 | Bertrix | 49.5037597,5.1557164 | Potato leaf |
| 44L-B7 | Bertrix | 49.5037597,5.1557164 | Potato leaf |
| 44L-B8 | Bertrix | 49.5037597,5.1557164 | Potato leaf |
| 44L-B9 | Bertrix | 49.5037597,5.1557164 | Potato leaf |
| 44T-B3 | Bertrix | 49.5037597,5.1557164 | Potato tuber |
| 44T-B4 | Bertrix | 49.5037597,5.1557164 | Potato tuber |
| 44T-B3 | Bertrix | 49.5037597,5.1557164 | Potato tuber |
| 44T-B4 | Bertrix | 49.5037597,5.1557164 | Potato tuber |
| 44T-B5 | Bertrix | 49.5037597,5.1557164 | Potato tuber |
| 44T-B6 | Bertrix | 49.5037597,5.1557164 | Potato tuber |
| 44T-B7 | Bertrix | 49.5037597,5.1557164 | Potato tuber |
| 44T-B8 | Bertrix | 49.5037597,5.1557164 | Potato tuber |
| 44T-B9 | Bertrix | 49.5037597,5.1557164 | Potato tuber |
| 45R-B1 | Bertrix | 49.4655096,5.1249068 | Potato root |
| 45R-B2 | Bertrix | 49.4655096,5.1249068 | Potato root |
| 45R-B3 | Bertrix | 49.4655096,5.1249068 | Potato root |
| 45R-B4 | Bertrix | 49.4655096,5.1249068 | Potato root |
| 45R-B5 | Bertrix | 49.4655096,5.1249068 | Potato root |
| 45R-B6 | Bertrix | 49.4655096,5.1249068 | Potato root |
| 45R-B7 | Bertrix | 49.4655096,5.1249068 | Potato root |
| 45R-B8 | Bertrix | 49.4655096,5.1249068 | Potato root |
| 45R-B9 | Bertrix | 49.4655096,5.1249068 | Potato root |
| 45L-B1 | Bertrix | 49.4655096,5.1249068 | Potato leaf |
| 45L-B2 | Bertrix | 49.4655096,5.1249068 | Potato leaf |
| 45L-B3 | Bertrix | 49.4655096,5.1249068 | Potato leaf |
| 45L-B4 | Bertrix | 49.4655096,5.1249068 | Potato leaf |
| 45L-B5 | Bertrix | 49.4655096,5.1249068 | Potato leaf |
| 45L-B6 | Bertrix | 49.4655096,5.1249068 | Potato leaf |
| 45L-B7 | Bertrix | 49.4655096,5.1249068 | Potato leaf |
| 45L-B8 | Bertrix | 49.4655096,5.1249068 | Potato leaf |
| 45L-B9 | Bertrix | 49.4655096,5.1249068 | Potato leaf |
| 45T-B1 | Bertrix | 49.4655096,5.1249068 | Potato tuber |
| 45T-B2 | Bertrix | 49.4655096,5.1249068 | Potato tuber |
| 45T-B3 | Bertrix | 49.4655096,5.1249068 | Potato tuber |
| 45T-B4 | Bertrix | 49.4655096,5.1249068 | Potato tuber |
| 45T-B5 | Bertrix | 49.4655096,5.1249068 | Potato tuber |
| 45T-B6 | Bertrix | 49.4655096,5.1249068 | Potato tuber |
| 45T-B7 | Bertrix | 49.4655096,5.1249068 | Potato tuber |
| 45T-B8 | Bertrix | 49.4655096,5.1249068 | Potato tuber |
| 45T-B9 | Bertrix | 49.4655096,5.1249068 | Potato tuber |
| 46R-B1 | Ath | 50.645821,3.856544 | Potato root |
| 46R-B2 | Ath | 50.645821,3.856544 | Potato root |
| 46R-B3 | Ath | 50.645821,3.856544 | Potato root |
| 46R-B4 | Ath | 50.645821,3.856544 | Potato root |
| 46R-B5 | Ath | 50.645821,3.856544 | Potato root |
| 46R-B6 | Ath | 50.645821,3.856544 | Potato root |
| 46R-B7 | Ath | 50.645821,3.856544 | Potato root |
| 46R-B8 | Ath | 50.645821,3.856544 | Potato root |
| 46R-B9 | Ath | 50.645821,3.856544 | Potato root |
| 46L-B1 | Ath | 50.645821,3.856544 | Potato leaf |
| 46L-B2 | Ath | 50.645821,3.856544 | Potato leaf |
| 46L-B3 | Ath | 50.645821,3.856544 | Potato leaf |
| 46L-B4 | Ath | 50.645821,3.856544 | Potato leaf |
| 46L-B5 | Ath | 50.645821,3.856544 | Potato leaf |
| 46L-B6 | Ath | 50.645821,3.856544 | Potato leaf |
| 46L-B7 | Ath | 50.645821,3.856544 | Potato leaf |
| 46L-B8 | Ath | 50.645821,3.856544 | Potato leaf |
| 46L-B9 | Ath | 50.645821,3.856544 | Potato leaf |
| 46T-B1 | Ath | 50.645821,3.856544 | Potato tuber |
| 46T-B2 | Ath | 50.645821,3.856544 | Potato tuber |
| 46T-B3 | Ath | 50.645821,3.856544 | Potato tuber |
| 46T-B4 | Ath | 50.645821,3.856544 | Potato tuber |
| 46T-B5 | Ath | 50.645821,3.856544 | Potato tuber |
| 46T-B6 | Ath | 50.645821,3.856544 | Potato tuber |
| 46T-B7 | Ath | 50.645821,3.856544 | Potato tuber |
| 46T-B8 | Ath | 50.645821,3.856544 | Potato tuber |
| 46T-B9 | Ath | 50.645821,3.856544 | Potato tuber |
| 47R-B1 | Lens | 50.586615,3.906841 | Potato root |
| 47R-B2 | Lens | 50.586615,3.906841 | Potato root |
| 47R-B3 | Lens | 50.586615,3.906841 | Potato root |
| 47R-B4 | Lens | 50.586615,3.906841 | Potato root |
| 47R-B5 | Lens | 50.586615,3.906841 | Potato root |
| 47R-B6 | Lens | 50.586615,3.906841 | Potato root |
| 47R-B7 | Lens | 50.586615,3.906841 | Potato root |
| 47R-B8 | Lens | 50.586615,3.906841 | Potato root |
| 47R-B9 | Lens | 50.586615,3.906841 | Potato root |
| 47L-B1 | Lens | 50.586615,3.906841 | Potato leaf |
| 47L-B2 | Lens | 50.586615,3.906841 | Potato leaf |
| 47L-B3 | Lens | 50.586615,3.906841 | Potato leaf |
| 47L-B4 | Lens | 50.586615,3.906841 | Potato leaf |
| 47L-B5 | Lens | 50.586615,3.906841 | Potato leaf |
| 47L-B6 | Lens | 50.586615,3.906841 | Potato leaf |
| 47L-B7 | Lens | 50.586615,3.906841 | Potato leaf |
| 47L-B8 | Lens | 50.586615,3.906841 | Potato leaf |
| 47L-B9 | Lens | 50.586615,3.906841 | Potato leaf |
| 47T-B1 | Lens | 50.586615,3.906841 | Potato tuber |
| 47T-B2 | Lens | 50.586615,3.906841 | Potato tuber |
| 47T-B3 | Lens | 50.586615,3.906841 | Potato tuber |
| 47T-B4 | Lens | 50.586615,3.906841 | Potato tuber |
| 47T-B5 | Lens | 50.586615,3.906841 | Potato tuber |
| 47T-B6 | Lens | 50.586615,3.906841 | Potato tuber |
| 47T-B7 | Lens | 50.586615,3.906841 | Potato tuber |
| 47T-B8 | Lens | 50.586615,3.906841 | Potato tuber |
| 47T-B9 | Lens | 50.586615,3.906841 | Potato tuber |
| 48R-B1 | Soignies | 50.563978,4.017375 | Potato root |
| 48R-B2 | Soignies | 50.563978,4.017375 | Potato root |
| 48R-B3 | Soignies | 50.563978,4.017375 | Potato root |
| 48R-B4 | Soignies | 50.563978,4.017375 | Potato root |
| 48R-B5 | Soignies | 50.563978,4.017375 | Potato root |
| 48R-B6 | Soignies | 50.563978,4.017375 | Potato root |
| 48R-B7 | Soignies | 50.563978,4.017375 | Potato root |
| 48R-B8 | Soignies | 50.563978,4.017375 | Potato root |
| 48R-B9 | Soignies | 50.563978,4.017375 | Potato root |
| 48L-B1 | Soignies | 50.563978,4.017375 | Potato leaf |
| 48L-B2 | Soignies | 50.563978,4.017375 | Potato leaf |
| 48L-B3 | Soignies | 50.563978,4.017375 | Potato leaf |
| 48L-B4 | Soignies | 50.563978,4.017375 | Potato leaf |
| 48L-B5 | Soignies | 50.563978,4.017375 | Potato leaf |
| 48L-B6 | Soignies | 50.563978,4.017375 | Potato leaf |
| 48L-B7 | Soignies | 50.563978,4.017375 | Potato leaf |
| 48L-B8 | Soignies | 50.563978,4.017375 | Potato leaf |
| 48L-B9 | Soignies | 50.563978,4.017375 | Potato leaf |
| 48T-B1 | Soignies | 50.563978,4.017375 | Potato tuber |
| 48T-B2 | Soignies | 50.563978,4.017375 | Potato tuber |
| 48T-B3 | Soignies | 50.563978,4.017375 | Potato tuber |
| 48T-B4 | Soignies | 50.563978,4.017375 | Potato tuber |
| 48T-B5 | Soignies | 50.563978,4.017375 | Potato tuber |
| 48T-B6 | Soignies | 50.563978,4.017375 | Potato tuber |
| 48T-B7 | Soignies | 50.563978,4.017375 | Potato tuber |
| 48T-B8 | Soignies | 50.563978,4.017375 | Potato tuber |
| 48T-B9 | Soignies | 50.563978,4.017375 | Potato tuber |
| 49R-B3 | Chièvres | 50.550877,3.825468 | Potato root |
| 49R-B4 | Chièvres | 50.550877,3.825468 | Potato root |
| 49R-B3 | Chièvres | 50.550877,3.825468 | Potato root |
| 49R-B4 | Chièvres | 50.550877,3.825468 | Potato root |
| 49R-B5 | Chièvres | 50.550877,3.825468 | Potato root |
| 49R-B6 | Chièvres | 50.550877,3.825468 | Potato root |
| 49R-B7 | Chièvres | 50.550877,3.825468 | Potato root |
| 49R-B8 | Chièvres | 50.550877,3.825468 | Potato root |
| 49R-B9 | Chièvres | 50.550877,3.825468 | Potato root |
| 49L-B3 | Chièvres | 50.550877,3.825468 | Potato leaf |
| 49L-B4 | Chièvres | 50.550877,3.825468 | Potato leaf |
| 49L-B3 | Chièvres | 50.550877,3.825468 | Potato leaf |
| 49L-B4 | Chièvres | 50.550877,3.825468 | Potato leaf |
| 49L-B5 | Chièvres | 50.550877,3.825468 | Potato leaf |
| 49L-B6 | Chièvres | 50.550877,3.825468 | Potato leaf |
| 49L-B7 | Chièvres | 50.550877,3.825468 | Potato leaf |
| 49L-B8 | Chièvres | 50.550877,3.825468 | Potato leaf |
| 49L-B9 | Chièvres | 50.550877,3.825468 | Potato leaf |
| 49T-B3 | Chièvres | 50.550877,3.825468 | Potato tuber |
| 49T-B4 | Chièvres | 50.550877,3.825468 | Potato tuber |
| 49T-B3 | Chièvres | 50.550877,3.825468 | Potato tuber |
| 49T-B4 | Chièvres | 50.550877,3.825468 | Potato tuber |
| 49T-B5 | Chièvres | 50.550877,3.825468 | Potato tuber |
| 49T-B6 | Chièvres | 50.550877,3.825468 | Potato tuber |
| 49T-B7 | Chièvres | 50.550877,3.825468 | Potato tuber |
| 49T-B8 | Chièvres | 50.550877,3.825468 | Potato tuber |
| 49T-B9 | Chièvres | 50.550877,3.825468 | Potato tuber |
| 50R-B3 | Ath | 50.595933,3.710418 | Potato root |
| 50R-B4 | Ath | 50.595933,3.710418 | Potato root |
| 50R-B3 | Ath | 50.595933,3.710418 | Potato root |
| 50R-B4 | Ath | 50.595933,3.710418 | Potato root |
| 50R-B5 | Ath | 50.595933,3.710418 | Potato root |
| 50R-B6 | Ath | 50.595933,3.710418 | Potato root |
| 50R-B7 | Ath | 50.595933,3.710418 | Potato root |
| 50R-B8 | Ath | 50.595933,3.710418 | Potato root |
| 50R-B9 | Ath | 50.595933,3.710418 | Potato root |
| 50L-B3 | Ath | 50.595933,3.710418 | Potato leaf |
| 50L-B4 | Ath | 50.595933,3.710418 | Potato leaf |
| 50L-B3 | Ath | 50.595933,3.710418 | Potato leaf |
| 50L-B4 | Ath | 50.595933,3.710418 | Potato leaf |
| 50L-B5 | Ath | 50.595933,3.710418 | Potato leaf |
| 50L-B6 | Ath | 50.595933,3.710418 | Potato leaf |
| 50L-B7 | Ath | 50.595933,3.710418 | Potato leaf |
| 50L-B8 | Ath | 50.595933,3.710418 | Potato leaf |
| 50L-B9 | Ath | 50.595933,3.710418 | Potato leaf |
| 50T-B3 | Ath | 50.595933,3.710418 | Potato tuber |
| 50T-B4 | Ath | 50.595933,3.710418 | Potato tuber |
| 50T-B3 | Ath | 50.595933,3.710418 | Potato tuber |
| 50T-B4 | Ath | 50.595933,3.710418 | Potato tuber |
| 50T-B5 | Ath | 50.595933,3.710418 | Potato tuber |
| 50T-B6 | Ath | 50.595933,3.710418 | Potato tuber |
| 50T-B7 | Ath | 50.595933,3.710418 | Potato tuber |
| 50T-B8 | Ath | 50.595933,3.710418 | Potato tuber |
| 50T-B9 | Ath | 50.595933,3.710418 | Potato tuber |
| 51R-B1 | Habay | 49.737759,5.616503 | Potato root |
| 51R-B2 | Habay | 49.737759,5.616503 | Potato root |
| 51R-B3 | Habay | 49.737759,5.616503 | Potato root |
| 51R-B4 | Habay | 49.737759,5.616503 | Potato root |
| 51R-B5 | Habay | 49.737759,5.616503 | Potato root |
| 51R-B6 | Habay | 49.737759,5.616503 | Potato root |
| 51R-B7 | Habay | 49.737759,5.616503 | Potato root |
| 51R-B8 | Habay | 49.737759,5.616503 | Potato root |
| 51R-B9 | Habay | 49.737759,5.616503 | Potato root |
| 51L-B1 | Habay | 49.737759,5.616503 | Potato leaf |
| 51L-B2 | Habay | 49.737759,5.616503 | Potato leaf |
| 51L-B3 | Habay | 49.737759,5.616503 | Potato leaf |
| 51L-B4 | Habay | 49.737759,5.616503 | Potato leaf |
| 51L-B5 | Habay | 49.737759,5.616503 | Potato leaf |
| 51L-B6 | Habay | 49.737759,5.616503 | Potato leaf |
| 51L-B7 | Habay | 49.737759,5.616503 | Potato leaf |
| 51L-B8 | Habay | 49.737759,5.616503 | Potato leaf |
| 51L-B9 | Habay | 49.737759,5.616503 | Potato leaf |
| 51T-B1 | Habay | 49.737759,5.616503 | Potato tuber |
| 51T-B2 | Habay | 49.737759,5.616503 | Potato tuber |
| 51T-B3 | Habay | 49.737759,5.616503 | Potato tuber |
| 51T-B4 | Habay | 49.737759,5.616503 | Potato tuber |
| 51T-B5 | Habay | 49.737759,5.616503 | Potato tuber |
| 51T-B6 | Habay | 49.737759,5.616503 | Potato tuber |
| 51T-B7 | Habay | 49.737759,5.616503 | Potato tuber |
| 51T-B8 | Habay | 49.737759,5.616503 | Potato tuber |
| 51T-B9 | Habay | 49.737759,5.616503 | Potato tuber |
| 52R-B1 | Habay | 49.737829,5.615934 | Potato root |
| 52R-B2 | Habay | 49.737829,5.615934 | Potato root |
| 52R-B3 | Habay | 49.737829,5.615934 | Potato root |
| 52R-B4 | Habay | 49.737829,5.615934 | Potato root |
| 52R-B5 | Habay | 49.737829,5.615934 | Potato root |
| 52R-B6 | Habay | 49.737829,5.615934 | Potato root |
| 52R-B7 | Habay | 49.737829,5.615934 | Potato root |
| 52R-B8 | Habay | 49.737829,5.615934 | Potato root |
| 52R-B9 | Habay | 49.737829,5.615934 | Potato root |
| 52L-B1 | Habay | 49.737829,5.615934 | Potato leaf |
| 52L-B2 | Habay | 49.737829,5.615934 | Potato leaf |
| 52L-B3 | Habay | 49.737829,5.615934 | Potato leaf |
| 52L-B4 | Habay | 49.737829,5.615934 | Potato leaf |
| 52L-B5 | Habay | 49.737829,5.615934 | Potato leaf |
| 52L-B6 | Habay | 49.737829,5.615934 | Potato leaf |
| 52L-B7 | Habay | 49.737829,5.615934 | Potato leaf |
| 52L-B8 | Habay | 49.737829,5.615934 | Potato leaf |
| 52L-B9 | Habay | 49.737829,5.615934 | Potato leaf |
| 52T-B1 | Habay | 49.737829,5.615934 | Potato tuber |
| 52T-B2 | Habay | 49.737829,5.615934 | Potato tuber |
| 52T-B3 | Habay | 49.737829,5.615934 | Potato tuber |
| 52T-B4 | Habay | 49.737829,5.615934 | Potato tuber |
| 52T-B5 | Habay | 49.737829,5.615934 | Potato tuber |
| 52T-B6 | Habay | 49.737829,5.615934 | Potato tuber |
| 52T-B7 | Habay | 49.737829,5.615934 | Potato tuber |
| 52T-B8 | Habay | 49.737829,5.615934 | Potato tuber |
| 52T-B9 | Habay | 49.737829,5.615934 | Potato tuber |
| 53R-B1 | Habay | 49.724403,5.601751 | Potato root |
| 53R-B2 | Habay | 49.724403,5.601751 | Potato root |
| 53R-B3 | Habay | 49.724403,5.601751 | Potato root |
| 53R-B4 | Habay | 49.724403,5.601751 | Potato root |
| 53R-B5 | Habay | 49.724403,5.601751 | Potato root |
| 53R-B6 | Habay | 49.724403,5.601751 | Potato root |
| 53R-B7 | Habay | 49.724403,5.601751 | Potato root |
| 53R-B8 | Habay | 49.724403,5.601751 | Potato root |
| 53R-B9 | Habay | 49.724403,5.601751 | Potato root |
| 53L-B1 | Habay | 49.724403,5.601751 | Potato leaf |
| 53L-B2 | Habay | 49.724403,5.601751 | Potato leaf |
| 53L-B3 | Habay | 49.724403,5.601751 | Potato leaf |
| 53L-B4 | Habay | 49.724403,5.601751 | Potato leaf |
| 53L-B5 | Habay | 49.724403,5.601751 | Potato leaf |
| 53L-B6 | Habay | 49.724403,5.601751 | Potato leaf |
| 53L-B7 | Habay | 49.724403,5.601751 | Potato leaf |
| 53L-B8 | Habay | 49.724403,5.601751 | Potato leaf |
| 53L-B9 | Habay | 49.724403,5.601751 | Potato leaf |
| 53T-B1 | Habay | 49.724403,5.601751 | Potato tuber |
| 53T-B2 | Habay | 49.724403,5.601751 | Potato tuber |
| 53T-B3 | Habay | 49.724403,5.601751 | Potato tuber |
| 53T-B4 | Habay | 49.724403,5.601751 | Potato tuber |
| 53T-B5 | Habay | 49.724403,5.601751 | Potato tuber |
| 53T-B6 | Habay | 49.724403,5.601751 | Potato tuber |
| 53T-B7 | Habay | 49.724403,5.601751 | Potato tuber |
| 53T-B8 | Habay | 49.724403,5.601751 | Potato tuber |
| 53T-B9 | Habay | 49.724403,5.601751 | Potato tuber |
| P1A-B1 | Beauraing | 50.107616,4.974082 | Vegetable garden |
| P1A-B2 | Beauraing | 50.107616,4.974082 | Vegetable garden |
| P1A-B3 | Beauraing | 50.107616,4.974082 | Vegetable garden |
| P1A-B4 | Beauraing | 50.107616,4.974082 | Vegetable garden |
| P1A-B5 | Beauraing | 50.107616,4.974082 | Vegetable garden |
| P1A-B6 | Beauraing | 50.107616,4.974082 | Vegetable garden |
| P1A-B7 | Beauraing | 50.107616,4.974082 | Vegetable garden |
| P1A-B8 | Beauraing | 50.107616,4.974082 | Vegetable garden |
| P1A-B9 | Beauraing | 50.107616,4.974082 | Vegetable garden |
| P2A-B1 | Bertrix | 49.85128,5.25257 | Vegetable garden |
| P2A-B2 | Bertrix | 49.85128,5.25257 | Vegetable garden |
| P2A-B3 | Bertrix | 49.85128,5.25257 | Vegetable garden |
| P2A-B4 | Bertrix | 49.85128,5.25257 | Vegetable garden |
| P2A-B5 | Bertrix | 49.85128,5.25257 | Vegetable garden |
| P2A-B6 | Bertrix | 49.85128,5.25257 | Vegetable garden |
| P2A-B7 | Bertrix | 49.85128,5.25257 | Vegetable garden |
| P2A-B8 | Bertrix | 49.85128,5.25257 | Vegetable garden |
| P2A-B9 | Bertrix | 49.85128,5.25257 | Vegetable garden |
| P3A-B1 | Bertrix | 49.85128,5.25257 | Vegetable garden |
| P3A-B2 | Bertrix | 49.85128,5.25257 | Vegetable garden |
| P3A-B3 | Bertrix | 49.85128,5.25257 | Vegetable garden |
| P3A-B4 | Bertrix | 49.85128,5.25257 | Vegetable garden |
| P3A-B5 | Bertrix | 49.85128,5.25257 | Vegetable garden |
| P3A-B6 | Bertrix | 49.85128,5.25257 | Vegetable garden |
| P3A-B7 | Bertrix | 49.85128,5.25257 | Vegetable garden |
| P3A-B8 | Bertrix | 49.85128,5.25257 | Vegetable garden |
| P3A-B9 | Bertrix | 49.85128,5.25257 | Vegetable garden |
| P4A-B1 | Bertrix | 49.84403,5.265884 | Vegetable garden |
| P4A-B2 | Bertrix | 49.84403,5.265884 | Vegetable garden |
| P4A-B3 | Bertrix | 49.84403,5.265884 | Vegetable garden |
| P4A-B4 | Bertrix | 49.84403,5.265884 | Vegetable garden |
| P4A-B5 | Bertrix | 49.84403,5.265884 | Vegetable garden |
| P4A-B6 | Bertrix | 49.84403,5.265884 | Vegetable garden |
| P4A-B7 | Bertrix | 49.84403,5.265884 | Vegetable garden |
| P4A-B8 | Bertrix | 49.84403,5.265884 | Vegetable garden |
| P4A-B9 | Bertrix | 49.84403,5.265884 | Vegetable garden |
| P5A-B1 | Bertrix | 49.782615,5.213131 | Vegetable garden |
| P5A-B2 | Bertrix | 49.782615,5.213131 | Vegetable garden |
| P5A-B3 | Bertrix | 49.782615,5.213131 | Vegetable garden |
| P5A-B4 | Bertrix | 49.782615,5.213131 | Vegetable garden |
| P5A-B5 | Bertrix | 49.782615,5.213131 | Vegetable garden |
| P5A-B6 | Bertrix | 49.782615,5.213131 | Vegetable garden |
| P5A-B7 | Bertrix | 49.782615,5.213131 | Vegetable garden |
| P5A-B8 | Bertrix | 49.782615,5.213131 | Vegetable garden |
| P5A-B9 | Bertrix | 49.782615,5.213131 | Vegetable garden |
| P1B-B1 | Beauraing | 50.107616,4.974082 | Vegetable garden |
| P1B-B2 | Beauraing | 50.107616,4.974082 | Vegetable garden |
| P1B-B3 | Beauraing | 50.107616,4.974082 | Vegetable garden |
| P1B-B4 | Beauraing | 50.107616,4.974082 | Vegetable garden |
| P1B-B5 | Beauraing | 50.107616,4.974082 | Vegetable garden |
| P1B-B6 | Beauraing | 50.107616,4.974082 | Vegetable garden |
| P1B-B7 | Beauraing | 50.107616,4.974082 | Vegetable garden |
| P1B-B8 | Beauraing | 50.107616,4.974082 | Vegetable garden |
| P1B-B9 | Beauraing | 50.107616,4.974082 | Vegetable garden |
| P2B-B1 | Bertrix | 49.85128,5.25257 | Vegetable garden |
| P2B-B2 | Bertrix | 49.85128,5.25257 | Vegetable garden |
| P2B-B3 | Bertrix | 49.85128,5.25257 | Vegetable garden |
| P2B-B4 | Bertrix | 49.85128,5.25257 | Vegetable garden |
| P2B-B5 | Bertrix | 49.85128,5.25257 | Vegetable garden |
| P2B-B6 | Bertrix | 49.85128,5.25257 | Vegetable garden |
| P2B-B7 | Bertrix | 49.85128,5.25257 | Vegetable garden |
| P2B-B8 | Bertrix | 49.85128,5.25257 | Vegetable garden |
| P2B-B9 | Bertrix | 49.85128,5.25257 | Vegetable garden |
| P3B-B1 | Bertrix | 49.85128,5.25257 | Vegetable garden |
| P3B-B2 | Bertrix | 49.85128,5.25257 | Vegetable garden |
| P3B-B3 | Bertrix | 49.85128,5.25257 | Vegetable garden |
| P3B-B4 | Bertrix | 49.85128,5.25257 | Vegetable garden |
| P3B-B5 | Bertrix | 49.85128,5.25257 | Vegetable garden |
| P3B-B6 | Bertrix | 49.85128,5.25257 | Vegetable garden |
| P3B-B7 | Bertrix | 49.85128,5.25257 | Vegetable garden |
| P3B-B8 | Bertrix | 49.85128,5.25257 | Vegetable garden |
| P3B-B9 | Bertrix | 49.85128,5.25257 | Vegetable garden |
| P4B-B1 | Bertrix | 49.84403,5.265884 | Vegetable garden |
| P4B-B2 | Bertrix | 49.84403,5.265884 | Vegetable garden |
| P4B-B3 | Bertrix | 49.84403,5.265884 | Vegetable garden |
| P4B-B4 | Bertrix | 49.84403,5.265884 | Vegetable garden |
| P4B-B5 | Bertrix | 49.84403,5.265884 | Vegetable garden |
| P4B-B6 | Bertrix | 49.84403,5.265884 | Vegetable garden |
| P4B-B7 | Bertrix | 49.84403,5.265884 | Vegetable garden |
| P4B-B8 | Bertrix | 49.84403,5.265884 | Vegetable garden |
| P4B-B9 | Bertrix | 49.84403,5.265884 | Vegetable garden |
| P5B-B1 | Bertrix | 49.782615,5.213131 | Vegetable garden |
| P5B-B2 | Bertrix | 49.782615,5.213131 | Vegetable garden |
| P5B-B3 | Bertrix | 49.782615,5.213131 | Vegetable garden |
| P5B-B4 | Bertrix | 49.782615,5.213131 | Vegetable garden |
| P5B-B5 | Bertrix | 49.782615,5.213131 | Vegetable garden |
| P5B-B6 | Bertrix | 49.782615,5.213131 | Vegetable garden |
| P5B-B7 | Bertrix | 49.782615,5.213131 | Vegetable garden |
| P5B-B8 | Bertrix | 49.782615,5.213131 | Vegetable garden |
| P5B-B9 | Bertrix | 49.782615,5.213131 | Vegetable garden |
| 1A-P1 | Tubize | 49.4655096,5.1249068 | Crop field |
| 1A-P2 | Tubize | 49.4655096,5.1249068 | Crop field |
| 1A-P3 | Tubize | 49.4655096,5.1249068 | Crop field |
| 1A-P4 | Tubize | 49.4655096,5.1249068 | Crop field |
| 1A-P5 | Tubize | 49.4655096,5.1249068 | Crop field |
| 1A-P6 | Tubize | 49.4655096,5.1249068 | Crop field |
| 1A-P7 | Tubize | 49.4655096,5.1249068 | Crop field |
| 1A-P8 | Tubize | 49.4655096,5.1249068 | Crop field |
| 1A-P9 | Tubize | 49.4655096,5.1249068 | Crop field |
| 2A-P1 | Tubize | 49.4655096,5.1249068 | Crop field |
| 2A-P2 | Tubize | 49.4655096,5.1249068 | Crop field |
| 2A-P3 | Tubize | 49.4655096,5.1249068 | Crop field |
| 2A-P4 | Tubize | 49.4655096,5.1249068 | Crop field |
| 2A-P5 | Tubize | 49.4655096,5.1249068 | Crop field |
| 2A-P6 | Tubize | 49.4655096,5.1249068 | Crop field |
| 2A-P7 | Tubize | 49.4655096,5.1249068 | Crop field |
| 2A-P8 | Tubize | 49.4655096,5.1249068 | Crop field |
| 2A-P9 | Tubize | 49.4655096,5.1249068 | Crop field |
| 3A-P1 | Rebecq | 49.4655096,5.1249068 | Crop field |
| 3A-P2 | Rebecq | 49.4655096,5.1249068 | Crop field |
| 3A-P3 | Rebecq | 49.4655096,5.1249068 | Crop field |
| 3A-P4 | Rebecq | 49.4655096,5.1249068 | Crop field |
| 3A-P5 | Rebecq | 49.4655096,5.1249068 | Crop field |
| 3A-P6 | Rebecq | 49.4655096,5.1249068 | Crop field |
| 3A-P7 | Rebecq | 49.4655096,5.1249068 | Crop field |
| 3A-P8 | Rebecq | 49.4655096,5.1249068 | Crop field |
| 3A-P9 | Rebecq | 49.4655096,5.1249068 | Crop field |
| 4A-P1 | Perwez | 49.4655096,5.1249068 | Crop field |
| 4A-P2 | Perwez | 49.4655096,5.1249068 | Crop field |
| 4A-P3 | Perwez | 49.4655096,5.1249068 | Crop field |
| 4A-P4 | Perwez | 49.4655096,5.1249068 | Crop field |
| 4A-P5 | Perwez | 49.4655096,5.1249068 | Crop field |
| 4A-P6 | Perwez | 49.4655096,5.1249068 | Crop field |
| 4A-P7 | Perwez | 49.4655096,5.1249068 | Crop field |
| 4A-P8 | Perwez | 49.4655096,5.1249068 | Crop field |
| 4A-P9 | Perwez | 49.4655096,5.1249068 | Crop field |
| 5A-P1 | Incourt | 49.4655096,5.1249068 | Crop field |
| 5A-P2 | Incourt | 49.4655096,5.1249068 | Crop field |
| 5A-P3 | Incourt | 49.4655096,5.1249068 | Crop field |
| 5A-P4 | Incourt | 49.4655096,5.1249068 | Crop field |
| 5A-P5 | Incourt | 49.4655096,5.1249068 | Crop field |
| 5A-P6 | Incourt | 49.4655096,5.1249068 | Crop field |
| 5A-P7 | Incourt | 49.4655096,5.1249068 | Crop field |
| 5A-P8 | Incourt | 49.4655096,5.1249068 | Crop field |
| 5A-P9 | Incourt | 49.4655096,5.1249068 | Crop field |
| 6A-P1 | Incourt | 49.4655096,5.1249068 | Crop field |
| 6A-P2 | Incourt | 49.4655096,5.1249068 | Crop field |
| 6A-P3 | Incourt | 49.4655096,5.1249068 | Crop field |
| 6A-P4 | Incourt | 49.4655096,5.1249068 | Crop field |
| 6A-P5 | Incourt | 49.4655096,5.1249068 | Crop field |
| 6A-P6 | Incourt | 49.4655096,5.1249068 | Crop field |
| 6A-P7 | Incourt | 49.4655096,5.1249068 | Crop field |
| 6A-P8 | Incourt | 49.4655096,5.1249068 | Crop field |
| 6A-P9 | Incourt | 49.4655096,5.1249068 | Crop field |
| 7A-P1 | Gembloux | 49.4655096,5.1249068 | Crop field |
| 7A-P2 | Gembloux | 49.4655096,5.1249068 | Crop field |
| 7A-P3 | Gembloux | 49.4655096,5.1249068 | Crop field |
| 7A-P4 | Gembloux | 49.4655096,5.1249068 | Crop field |
| 7A-P5 | Gembloux | 49.4655096,5.1249068 | Crop field |
| 7A-P6 | Gembloux | 49.4655096,5.1249068 | Crop field |
| 7A-P7 | Gembloux | 49.4655096,5.1249068 | Crop field |
| 7A-P8 | Gembloux | 49.4655096,5.1249068 | Crop field |
| 7A-P9 | Gembloux | 49.4655096,5.1249068 | Crop field |
| 8A-P1 | Gembloux | 49.4655096,5.1249068 | Crop field |
| 8A-P2 | Gembloux | 49.4655096,5.1249068 | Crop field |
| 8A-P3 | Gembloux | 49.4655096,5.1249068 | Crop field |
| 8A-P4 | Gembloux | 49.4655096,5.1249068 | Crop field |
| 8A-P5 | Gembloux | 49.4655096,5.1249068 | Crop field |
| 8A-P6 | Gembloux | 49.4655096,5.1249068 | Crop field |
| 8A-P7 | Gembloux | 49.4655096,5.1249068 | Crop field |
| 8A-P8 | Gembloux | 49.4655096,5.1249068 | Crop field |
| 8A-P9 | Gembloux | 49.4655096,5.1249068 | Crop field |
| 9A-P1 | Gembloux | 49.4655096,5.1249068 | Crop field |
| 9A-P2 | Gembloux | 49.4655096,5.1249068 | Crop field |
| 9A-P3 | Gembloux | 49.4655096,5.1249068 | Crop field |
| 9A-P4 | Gembloux | 49.4655096,5.1249068 | Crop field |
| 9A-P5 | Gembloux | 49.4655096,5.1249068 | Crop field |
| 9A-P6 | Gembloux | 49.4655096,5.1249068 | Crop field |
| 9A-P7 | Gembloux | 49.4655096,5.1249068 | Crop field |
| 9A-P8 | Gembloux | 49.4655096,5.1249068 | Crop field |
| 9A-P9 | Gembloux | 49.4655096,5.1249068 | Crop field |
| 10A-P1 | Gembloux | 49.4655096,5.1249068 | Crop field |
| 10A-P2 | Gembloux | 49.4655096,5.1249068 | Crop field |
| 10A-P3 | Gembloux | 49.4655096,5.1249068 | Crop field |
| 10A-P4 | Gembloux | 49.4655096,5.1249068 | Crop field |
| 10A-P5 | Gembloux | 49.4655096,5.1249068 | Crop field |
| 10A-P6 | Gembloux | 49.4655096,5.1249068 | Crop field |
| 10A-P7 | Gembloux | 49.4655096,5.1249068 | Crop field |
| 10A-P8 | Gembloux | 49.4655096,5.1249068 | Crop field |
| 10A-P9 | Gembloux | 49.4655096,5.1249068 | Crop field |
| 11A-P1 | Gembloux | 49.4655096,5.1249068 | Crop field |
| 11A-P2 | Gembloux | 49.4655096,5.1249068 | Crop field |
| 11A-P3 | Gembloux | 49.4655096,5.1249068 | Crop field |
| 11A-P4 | Gembloux | 49.4655096,5.1249068 | Crop field |
| 11A-P5 | Gembloux | 49.4655096,5.1249068 | Crop field |
| 11A-P6 | Gembloux | 49.4655096,5.1249068 | Crop field |
| 11A-P7 | Gembloux | 49.4655096,5.1249068 | Crop field |
| 11A-P8 | Gembloux | 49.4655096,5.1249068 | Crop field |
| 11A-P9 | Gembloux | 49.4655096,5.1249068 | Crop field |
| 12A-P1 | Gembloux | 49.4655096,5.1249068 | Crop field |
| 12A-P2 | Gembloux | 49.4655096,5.1249068 | Crop field |
| 12A-P3 | Gembloux | 49.4655096,5.1249068 | Crop field |
| 12A-P4 | Gembloux | 49.4655096,5.1249068 | Crop field |
| 12A-P5 | Gembloux | 49.4655096,5.1249068 | Crop field |
| 12A-P6 | Gembloux | 49.4655096,5.1249068 | Crop field |
| 12A-P7 | Gembloux | 49.4655096,5.1249068 | Crop field |
| 12A-P8 | Gembloux | 49.4655096,5.1249068 | Crop field |
| 12A-P9 | Gembloux | 49.4655096,5.1249068 | Crop field |
| 13A-P1 | Gembloux | 49.4655096,5.1249068 | Crop field |
| 13A-P2 | Gembloux | 49.4655096,5.1249068 | Crop field |
| 13A-P3 | Gembloux | 49.4655096,5.1249068 | Crop field |
| 13A-P4 | Gembloux | 49.4655096,5.1249068 | Crop field |
| 13A-P5 | Gembloux | 49.4655096,5.1249068 | Crop field |
| 13A-P6 | Gembloux | 49.4655096,5.1249068 | Crop field |
| 13A-P7 | Gembloux | 49.4655096,5.1249068 | Crop field |
| 13A-P8 | Gembloux | 49.4655096,5.1249068 | Crop field |
| 13A-P9 | Gembloux | 49.4655096,5.1249068 | Crop field |
| 14A-P1 | Cognelée | 49.4655096,5.1249068 | Crop field |
| 14A-P2 | Cognelée | 49.4655096,5.1249068 | Crop field |
| 14A-P3 | Cognelée | 49.4655096,5.1249068 | Crop field |
| 14A-P4 | Cognelée | 49.4655096,5.1249068 | Crop field |
| 14A-P5 | Cognelée | 49.4655096,5.1249068 | Crop field |
| 14A-P6 | Cognelée | 49.4655096,5.1249068 | Crop field |
| 14A-P7 | Cognelée | 49.4655096,5.1249068 | Crop field |
| 14A-P8 | Cognelée | 49.4655096,5.1249068 | Crop field |
| 14A-P9 | Cognelée | 49.4655096,5.1249068 | Crop field |
| 15A-P1 | Bousval | 49.4655096,5.1249068 | Crop field |
| 15A-P2 | Bousval | 49.4655096,5.1249068 | Crop field |
| 15A-P3 | Bousval | 49.4655096,5.1249068 | Crop field |
| 15A-P4 | Bousval | 49.4655096,5.1249068 | Crop field |
| 15A-P5 | Bousval | 49.4655096,5.1249068 | Crop field |
| 15A-P6 | Bousval | 49.4655096,5.1249068 | Crop field |
| 15A-P7 | Bousval | 49.4655096,5.1249068 | Crop field |
| 15A-P8 | Bousval | 49.4655096,5.1249068 | Crop field |
| 15A-P9 | Bousval | 49.4655096,5.1249068 | Crop field |
| 16A-P1 | Court st Etienne | 49.4655096,5.1249068 | Crop field |
| 16A-P2 | Court st Etienne | 49.4655096,5.1249068 | Crop field |
| 16A-P3 | Court st Etienne | 49.4655096,5.1249068 | Crop field |
| 16A-P4 | Court st Etienne | 49.4655096,5.1249068 | Crop field |
| 16A-P5 | Court st Etienne | 49.4655096,5.1249068 | Crop field |
| 16A-P6 | Court st Etienne | 49.4655096,5.1249068 | Crop field |
| 16A-P7 | Court st Etienne | 49.4655096,5.1249068 | Crop field |
| 16A-P8 | Court st Etienne | 49.4655096,5.1249068 | Crop field |
| 16A-P9 | Court st Etienne | 49.4655096,5.1249068 | Crop field |
| 17A-P1 | Court st Etienne | 49.4655096,5.1249068 | Crop field |
| 17A-P2 | Court st Etienne | 49.4655096,5.1249068 | Crop field |
| 17A-P3 | Court st Etienne | 49.4655096,5.1249068 | Crop field |
| 17A-P4 | Court st Etienne | 49.4655096,5.1249068 | Crop field |
| 17A-P5 | Court st Etienne | 49.4655096,5.1249068 | Crop field |
| 17A-P6 | Court st Etienne | 49.4655096,5.1249068 | Crop field |
| 17A-P7 | Court st Etienne | 49.4655096,5.1249068 | Crop field |
| 17A-P8 | Court st Etienne | 49.4655096,5.1249068 | Crop field |
| 17A-P9 | Court st Etienne | 49.4655096,5.1249068 | Crop field |
| 18A-P1 | Louvain-la-Neuve | 49.4655096,5.1249068 | Crop field |
| 18A-P2 | Louvain-la-Neuve | 49.4655096,5.1249068 | Crop field |
| 18A-P3 | Louvain-la-Neuve | 49.4655096,5.1249068 | Crop field |
| 18A-P4 | Louvain-la-Neuve | 49.4655096,5.1249068 | Crop field |
| 18A-P5 | Louvain-la-Neuve | 49.4655096,5.1249068 | Crop field |
| 18A-P6 | Louvain-la-Neuve | 49.4655096,5.1249068 | Crop field |
| 18A-P7 | Louvain-la-Neuve | 49.4655096,5.1249068 | Crop field |
| 18A-P8 | Louvain-la-Neuve | 49.4655096,5.1249068 | Crop field |
| 18A-P9 | Louvain-la-Neuve | 49.4655096,5.1249068 | Crop field |
| 19A-P1 | Coxyde | 49.4655096,5.1249068 | Crop field |
| 19A-P2 | Coxyde | 49.4655096,5.1249068 | Crop field |
| 19A-P3 | Coxyde | 49.4655096,5.1249068 | Crop field |
| 19A-P4 | Coxyde | 49.4655096,5.1249068 | Crop field |
| 19A-P5 | Coxyde | 49.4655096,5.1249068 | Crop field |
| 19A-P6 | Coxyde | 49.4655096,5.1249068 | Crop field |
| 19A-P7 | Coxyde | 49.4655096,5.1249068 | Crop field |
| 19A-P8 | Coxyde | 49.4655096,5.1249068 | Crop field |
| 19A-P9 | Coxyde | 49.4655096,5.1249068 | Crop field |
| 20A-P1 | Coxyde | 49.4655096,5.1249068 | Crop field |
| 20A-P2 | Coxyde | 49.4655096,5.1249068 | Crop field |
| 20A-P3 | Coxyde | 49.4655096,5.1249068 | Crop field |
| 20A-P4 | Coxyde | 49.4655096,5.1249068 | Crop field |
| 20A-P5 | Coxyde | 49.4655096,5.1249068 | Crop field |
| 20A-P6 | Coxyde | 49.4655096,5.1249068 | Crop field |
| 20A-P7 | Coxyde | 49.4655096,5.1249068 | Crop field |
| 20A-P8 | Coxyde | 49.4655096,5.1249068 | Crop field |
| 20A-P9 | Coxyde | 49.4655096,5.1249068 | Crop field |
| 21A-P1 | Coxyde | 49.4655096,5.1249068 | Crop field |
| 21A-P2 | Coxyde | 49.4655096,5.1249068 | Crop field |
| 21A-P3 | Coxyde | 49.4655096,5.1249068 | Crop field |
| 21A-P4 | Coxyde | 49.4655096,5.1249068 | Crop field |
| 21A-P5 | Coxyde | 49.4655096,5.1249068 | Crop field |
| 21A-P6 | Coxyde | 49.4655096,5.1249068 | Crop field |
| 21A-P7 | Coxyde | 49.4655096,5.1249068 | Crop field |
| 21A-P8 | Coxyde | 49.4655096,5.1249068 | Crop field |
| 21A-P9 | Coxyde | 49.4655096,5.1249068 | Crop field |
| 22A-P1 | Furne | 49.4655096,5.1249068 | Crop field |
| 22A-P2 | Furne | 49.4655096,5.1249068 | Crop field |
| 22A-P3 | Furne | 49.4655096,5.1249068 | Crop field |
| 22A-P4 | Furne | 49.4655096,5.1249068 | Crop field |
| 22A-P5 | Furne | 49.4655096,5.1249068 | Crop field |
| 22A-P6 | Furne | 49.4655096,5.1249068 | Crop field |
| 22A-P7 | Furne | 49.4655096,5.1249068 | Crop field |
| 22A-P8 | Furne | 49.4655096,5.1249068 | Crop field |
| 22A-P9 | Furne | 49.4655096,5.1249068 | Crop field |
| 23A-P1 | Furne | 49.4655096,5.1249068 | Crop field |
| 23A-P2 | Furne | 49.4655096,5.1249068 | Crop field |
| 23A-P3 | Furne | 49.4655096,5.1249068 | Crop field |
| 23A-P4 | Furne | 49.4655096,5.1249068 | Crop field |
| 23A-P5 | Furne | 49.4655096,5.1249068 | Crop field |
| 23A-P6 | Furne | 49.4655096,5.1249068 | Crop field |
| 23A-P7 | Furne | 49.4655096,5.1249068 | Crop field |
| 23A-P8 | Furne | 49.4655096,5.1249068 | Crop field |
| 23A-P9 | Furne | 49.4655096,5.1249068 | Crop field |
| 24A-P1 | Furne | 49.4655096,5.1249068 | Crop field |
| 24A-P2 | Furne | 49.4655096,5.1249068 | Crop field |
| 24A-P3 | Furne | 49.4655096,5.1249068 | Crop field |
| 24A-P4 | Furne | 49.4655096,5.1249068 | Crop field |
| 24A-P5 | Furne | 49.4655096,5.1249068 | Crop field |
| 24A-P6 | Furne | 49.4655096,5.1249068 | Crop field |
| 24A-P7 | Furne | 49.4655096,5.1249068 | Crop field |
| 24A-P8 | Furne | 49.4655096,5.1249068 | Crop field |
| 24A-P9 | Furne | 49.4655096,5.1249068 | Crop field |
| 25A-P1 | Furne | 49.4655096,5.1249068 | Crop field |
| 25A-P2 | Furne | 49.4655096,5.1249068 | Crop field |
| 25A-P3 | Furne | 49.4655096,5.1249068 | Crop field |
| 25A-P4 | Furne | 49.4655096,5.1249068 | Crop field |
| 25A-P5 | Furne | 49.4655096,5.1249068 | Crop field |
| 25A-P6 | Furne | 49.4655096,5.1249068 | Crop field |
| 25A-P7 | Furne | 49.4655096,5.1249068 | Crop field |
| 25A-P8 | Furne | 49.4655096,5.1249068 | Crop field |
| 25A-P9 | Furne | 49.4655096,5.1249068 | Crop field |
| 26A-P1 | Furne | 49.4655096,5.1249068 | Crop field |
| 26A-P2 | Furne | 49.4655096,5.1249068 | Crop field |
| 26A-P3 | Furne | 49.4655096,5.1249068 | Crop field |
| 26A-P4 | Furne | 49.4655096,5.1249068 | Crop field |
| 26A-P5 | Furne | 49.4655096,5.1249068 | Crop field |
| 26A-P6 | Furne | 49.4655096,5.1249068 | Crop field |
| 26A-P7 | Furne | 49.4655096,5.1249068 | Crop field |
| 26A-P8 | Furne | 49.4655096,5.1249068 | Crop field |
| 26A-P9 | Furne | 49.4655096,5.1249068 | Crop field |
| 27A-P1 | Waremme | n/a | Crop field |
| 27A-P2 | Waremme | n/a | Crop field |
| 27A-P3 | Waremme | n/a | Crop field |
| 27A-P4 | Waremme | n/a | Crop field |
| 27A-P5 | Waremme | n/a | Crop field |
| 27A-P6 | Waremme | n/a | Crop field |
| 27A-P7 | Waremme | n/a | Crop field |
| 27A-P8 | Waremme | n/a | Crop field |
| 27A-P9 | Waremme | n/a | Crop field |
| 28A-P1 | Waremme | n/a | Crop field |
| 28A-P2 | Waremme | n/a | Crop field |
| 28A-P3 | Waremme | n/a | Crop field |
| 28A-P4 | Waremme | n/a | Crop field |
| 28A-P5 | Waremme | n/a | Crop field |
| 28A-P6 | Waremme | n/a | Crop field |
| 28A-P7 | Waremme | n/a | Crop field |
| 28A-P8 | Waremme | n/a | Crop field |
| 28A-P9 | Waremme | n/a | Crop field |
| 29A-P1 | Waremme | n/a | Crop field |
| 29A-P2 | Waremme | n/a | Crop field |
| 29A-P3 | Waremme | n/a | Crop field |
| 29A-P4 | Waremme | n/a | Crop field |
| 29A-P5 | Waremme | n/a | Crop field |
| 29A-P6 | Waremme | n/a | Crop field |
| 29A-P7 | Waremme | n/a | Crop field |
| 29A-P8 | Waremme | n/a | Crop field |
| 29A-P9 | Waremme | n/a | Crop field |
| 30A-P1 | Waremme | n/a | Crop field |
| 30A-P2 | Waremme | n/a | Crop field |
| 30A-P3 | Waremme | n/a | Crop field |
| 30A-P4 | Waremme | n/a | Crop field |
| 30A-P5 | Waremme | n/a | Crop field |
| 30A-P6 | Waremme | n/a | Crop field |
| 30A-P7 | Waremme | n/a | Crop field |
| 30A-P8 | Waremme | n/a | Crop field |
| 30A-P9 | Waremme | n/a | Crop field |
| 31A-P1 | Soumagne | 49.4655096,5.1249068 | Crop field |
| 31A-P2 | Soumagne | 49.4655096,5.1249068 | Crop field |
| 31A-P3 | Soumagne | 49.4655096,5.1249068 | Crop field |
| 31A-P4 | Soumagne | 49.4655096,5.1249068 | Crop field |
| 31A-P5 | Soumagne | 49.4655096,5.1249068 | Crop field |
| 31A-P6 | Soumagne | 49.4655096,5.1249068 | Crop field |
| 31A-P7 | Soumagne | 49.4655096,5.1249068 | Crop field |
| 31A-P8 | Soumagne | 49.4655096,5.1249068 | Crop field |
| 31A-P9 | Soumagne | 49.4655096,5.1249068 | Crop field |
| 32A-P1 | Soumagne | 49.4655096,5.1249068 | Crop field |
| 32A-P2 | Soumagne | 49.4655096,5.1249068 | Crop field |
| 32A-P3 | Soumagne | 49.4655096,5.1249068 | Crop field |
| 32A-P4 | Soumagne | 49.4655096,5.1249068 | Crop field |
| 32A-P5 | Soumagne | 49.4655096,5.1249068 | Crop field |
| 32A-P6 | Soumagne | 49.4655096,5.1249068 | Crop field |
| 32A-P7 | Soumagne | 49.4655096,5.1249068 | Crop field |
| 32A-P8 | Soumagne | 49.4655096,5.1249068 | Crop field |
| 32A-P9 | Soumagne | 49.4655096,5.1249068 | Crop field |
| 33A-P1 | Soumagne | 49.4655096,5.1249068 | Crop field |
| 33A-P2 | Soumagne | 49.4655096,5.1249068 | Crop field |
| 33A-P3 | Soumagne | 49.4655096,5.1249068 | Crop field |
| 33A-P4 | Soumagne | 49.4655096,5.1249068 | Crop field |
| 33A-P5 | Soumagne | 49.4655096,5.1249068 | Crop field |
| 33A-P6 | Soumagne | 49.4655096,5.1249068 | Crop field |
| 33A-P7 | Soumagne | 49.4655096,5.1249068 | Crop field |
| 33A-P8 | Soumagne | 49.4655096,5.1249068 | Crop field |
| 33A-P9 | Soumagne | 49.4655096,5.1249068 | Crop field |
| 34A-P1 | Soumagne | 49.4655096,5.1249068 | Crop field |
| 34A-P2 | Soumagne | 49.4655096,5.1249068 | Crop field |
| 34A-P3 | Soumagne | 49.4655096,5.1249068 | Crop field |
| 34A-P4 | Soumagne | 49.4655096,5.1249068 | Crop field |
| 34A-P5 | Soumagne | 49.4655096,5.1249068 | Crop field |
| 34A-P6 | Soumagne | 49.4655096,5.1249068 | Crop field |
| 34A-P7 | Soumagne | 49.4655096,5.1249068 | Crop field |
| 34A-P8 | Soumagne | 49.4655096,5.1249068 | Crop field |
| 34A-P9 | Soumagne | 49.4655096,5.1249068 | Crop field |
| 35A-P1 | Soumagne | 49.4655096,5.1249068 | Crop field |
| 35A-P2 | Soumagne | 49.4655096,5.1249068 | Crop field |
| 35A-P3 | Soumagne | 49.4655096,5.1249068 | Crop field |
| 35A-P4 | Soumagne | 49.4655096,5.1249068 | Crop field |
| 35A-P5 | Soumagne | 49.4655096,5.1249068 | Crop field |
| 35A-P6 | Soumagne | 49.4655096,5.1249068 | Crop field |
| 35A-P7 | Soumagne | 49.4655096,5.1249068 | Crop field |
| 35A-P8 | Soumagne | 49.4655096,5.1249068 | Crop field |
| 35A-P9 | Soumagne | 49.4655096,5.1249068 | Crop field |
| 36A-P1 | Rebecq | 49.4655096,5.1249068 | Crop field |
| 36A-P2 | Rebecq | 49.4655096,5.1249068 | Crop field |
| 36A-P3 | Rebecq | 49.4655096,5.1249068 | Crop field |
| 36A-P4 | Rebecq | 49.4655096,5.1249068 | Crop field |
| 36A-P5 | Rebecq | 49.4655096,5.1249068 | Crop field |
| 36A-P6 | Rebecq | 49.4655096,5.1249068 | Crop field |
| 36A-P7 | Rebecq | 49.4655096,5.1249068 | Crop field |
| 36A-P8 | Rebecq | 49.4655096,5.1249068 | Crop field |
| 36A-P9 | Rebecq | 49.4655096,5.1249068 | Crop field |
| 37A-P1 | Rebecq | 49.4655096,5.1249068 | Crop field |
| 37A-P2 | Rebecq | 49.4655096,5.1249068 | Crop field |
| 37A-P3 | Rebecq | 49.4655096,5.1249068 | Crop field |
| 37A-P4 | Rebecq | 49.4655096,5.1249068 | Crop field |
| 37A-P5 | Rebecq | 49.4655096,5.1249068 | Crop field |
| 37A-P6 | Rebecq | 49.4655096,5.1249068 | Crop field |
| 37A-P7 | Rebecq | 49.4655096,5.1249068 | Crop field |
| 37A-P8 | Rebecq | 49.4655096,5.1249068 | Crop field |
| 37A-P9 | Rebecq | 49.4655096,5.1249068 | Crop field |
| 38A-P1 | Rebecq | 49.4655096,5.1249068 | Crop field |
| 38A-P2 | Rebecq | 49.4655096,5.1249068 | Crop field |
| 38A-P3 | Rebecq | 49.4655096,5.1249068 | Crop field |
| 38A-P4 | Rebecq | 49.4655096,5.1249068 | Crop field |
| 38A-P5 | Rebecq | 49.4655096,5.1249068 | Crop field |
| 38A-P6 | Rebecq | 49.4655096,5.1249068 | Crop field |
| 38A-P7 | Rebecq | 49.4655096,5.1249068 | Crop field |
| 38A-P8 | Rebecq | 49.4655096,5.1249068 | Crop field |
| 38A-P9 | Rebecq | 49.4655096,5.1249068 | Crop field |
| 39A-P1 | Soignies | 49.4655096,5.1249068 | Crop field |
| 39A-P2 | Soignies | 49.4655096,5.1249068 | Crop field |
| 39A-P3 | Soignies | 49.4655096,5.1249068 | Crop field |
| 39A-P4 | Soignies | 49.4655096,5.1249068 | Crop field |
| 39A-P5 | Soignies | 49.4655096,5.1249068 | Crop field |
| 39A-P6 | Soignies | 49.4655096,5.1249068 | Crop field |
| 39A-P7 | Soignies | 49.4655096,5.1249068 | Crop field |
| 39A-P8 | Soignies | 49.4655096,5.1249068 | Crop field |
| 39A-P9 | Soignies | 49.4655096,5.1249068 | Crop field |
| 40A-P1 | Soignies | 49.4655096,5.1249068 | Crop field |
| 40A-P2 | Soignies | 49.4655096,5.1249068 | Crop field |
| 40A-P3 | Soignies | 49.4655096,5.1249068 | Crop field |
| 40A-P4 | Soignies | 49.4655096,5.1249068 | Crop field |
| 40A-P5 | Soignies | 49.4655096,5.1249068 | Crop field |
| 40A-P6 | Soignies | 49.4655096,5.1249068 | Crop field |
| 40A-P7 | Soignies | 49.4655096,5.1249068 | Crop field |
| 40A-P8 | Soignies | 49.4655096,5.1249068 | Crop field |
| 40A-P9 | Soignies | 49.4655096,5.1249068 | Crop field |
| 41A-P1 | Bertrix | 49.848617,5.239266 | Crop field |
| 41A-P2 | Bertrix | 49.848617,5.239266 | Crop field |
| 41A-P3 | Bertrix | 49.848617,5.239266 | Crop field |
| 41A-P4 | Bertrix | 49.848617,5.239266 | Crop field |
| 41A-P5 | Bertrix | 49.848617,5.239266 | Crop field |
| 41A-P6 | Bertrix | 49.848617,5.239266 | Crop field |
| 41A-P7 | Bertrix | 49.848617,5.239266 | Crop field |
| 41A-P8 | Bertrix | 49.848617,5.239266 | Crop field |
| 41A-P9 | Bertrix | 49.848617,5.239266 | Crop field |
| 42A-P1 | Bertrix | 49.85128,5.25257 | Crop field |
| 42A-P2 | Bertrix | 49.85128,5.25257 | Crop field |
| 42A-P3 | Bertrix | 49.85128,5.25257 | Crop field |
| 42A-P4 | Bertrix | 49.85128,5.25257 | Crop field |
| 42A-P5 | Bertrix | 49.85128,5.25257 | Crop field |
| 42A-P6 | Bertrix | 49.85128,5.25257 | Crop field |
| 42A-P7 | Bertrix | 49.85128,5.25257 | Crop field |
| 42A-P8 | Bertrix | 49.85128,5.25257 | Crop field |
| 42A-P9 | Bertrix | 49.85128,5.25257 | Crop field |
| 43A-P1 | Bertrix | 49.85128,5.25257 | Crop field |
| 43A-P2 | Bertrix | 49.85128,5.25257 | Crop field |
| 43A-P3 | Bertrix | 49.85128,5.25257 | Crop field |
| 43A-P4 | Bertrix | 49.85128,5.25257 | Crop field |
| 43A-P5 | Bertrix | 49.85128,5.25257 | Crop field |
| 43A-P6 | Bertrix | 49.85128,5.25257 | Crop field |
| 43A-P7 | Bertrix | 49.85128,5.25257 | Crop field |
| 43A-P8 | Bertrix | 49.85128,5.25257 | Crop field |
| 43A-P9 | Bertrix | 49.85128,5.25257 | Crop field |
| 44A-P1 | Bertrix | 49.84403,5.265884 | Crop field |
| 44A-P2 | Bertrix | 49.84403,5.265884 | Crop field |
| 44A-P3 | Bertrix | 49.84403,5.265884 | Crop field |
| 44A-P4 | Bertrix | 49.84403,5.265884 | Crop field |
| 44A-P5 | Bertrix | 49.84403,5.265884 | Crop field |
| 44A-P6 | Bertrix | 49.84403,5.265884 | Crop field |
| 44A-P7 | Bertrix | 49.84403,5.265884 | Crop field |
| 44A-P8 | Bertrix | 49.84403,5.265884 | Crop field |
| 44A-P9 | Bertrix | 49.84403,5.265884 | Crop field |
| 45A-P1 | Bertrix | 49.782615,5.213131 | Crop field |
| 45A-P2 | Bertrix | 49.782615,5.213131 | Crop field |
| 45A-P3 | Bertrix | 49.782615,5.213131 | Crop field |
| 45A-P4 | Bertrix | 49.782615,5.213131 | Crop field |
| 45A-P5 | Bertrix | 49.782615,5.213131 | Crop field |
| 45A-P6 | Bertrix | 49.782615,5.213131 | Crop field |
| 45A-P7 | Bertrix | 49.782615,5.213131 | Crop field |
| 45A-P8 | Bertrix | 49.782615,5.213131 | Crop field |
| 45A-P9 | Bertrix | 49.782615,5.213131 | Crop field |
| 46A-P1 | Ath | 50.645821,3.856544 | Crop field |
| 46A-P2 | Ath | 50.645821,3.856544 | Crop field |
| 46A-P3 | Ath | 50.645821,3.856544 | Crop field |
| 46A-P4 | Ath | 50.645821,3.856544 | Crop field |
| 46A-P5 | Ath | 50.645821,3.856544 | Crop field |
| 46A-P6 | Ath | 50.645821,3.856544 | Crop field |
| 46A-P7 | Ath | 50.645821,3.856544 | Crop field |
| 46A-P8 | Ath | 50.645821,3.856544 | Crop field |
| 46A-P9 | Ath | 50.645821,3.856544 | Crop field |
| 47A-P1 | Lens | 50.586615,3.906841 | Crop field |
| 47A-P2 | Lens | 50.586615,3.906841 | Crop field |
| 47A-P3 | Lens | 50.586615,3.906841 | Crop field |
| 47A-P4 | Lens | 50.586615,3.906841 | Crop field |
| 47A-P5 | Lens | 50.586615,3.906841 | Crop field |
| 47A-P6 | Lens | 50.586615,3.906841 | Crop field |
| 47A-P7 | Lens | 50.586615,3.906841 | Crop field |
| 47A-P8 | Lens | 50.586615,3.906841 | Crop field |
| 47A-P9 | Lens | 50.586615,3.906841 | Crop field |
| 48A-P1 | Soignies | 50.563978,4.017375 | Crop field |
| 48A-P2 | Soignies | 50.563978,4.017375 | Crop field |
| 48A-P3 | Soignies | 50.563978,4.017375 | Crop field |
| 48A-P4 | Soignies | 50.563978,4.017375 | Crop field |
| 48A-P5 | Soignies | 50.563978,4.017375 | Crop field |
| 48A-P6 | Soignies | 50.563978,4.017375 | Crop field |
| 48A-P7 | Soignies | 50.563978,4.017375 | Crop field |
| 48A-P8 | Soignies | 50.563978,4.017375 | Crop field |
| 48A-P9 | Soignies | 50.563978,4.017375 | Crop field |
| 1B-P1 | Tubize | 49.4655096,5.1249068 | Crop field |
| 1B-P2 | Tubize | 49.4655096,5.1249068 | Crop field |
| 1B-P3 | Tubize | 49.4655096,5.1249068 | Crop field |
| 1B-P4 | Tubize | 49.4655096,5.1249068 | Crop field |
| 1B-P5 | Tubize | 49.4655096,5.1249068 | Crop field |
| 1B-P6 | Tubize | 49.4655096,5.1249068 | Crop field |
| 1B-P7 | Tubize | 49.4655096,5.1249068 | Crop field |
| 1B-P8 | Tubize | 49.4655096,5.1249068 | Crop field |
| 1B-P9 | Tubize | 49.4655096,5.1249068 | Crop field |
| 2B-P1 | Tubize | 49.4655096,5.1249068 | Crop field |
| 2B-P2 | Tubize | 49.4655096,5.1249068 | Crop field |
| 2B-P3 | Tubize | 49.4655096,5.1249068 | Crop field |
| 2B-P4 | Tubize | 49.4655096,5.1249068 | Crop field |
| 2B-P5 | Tubize | 49.4655096,5.1249068 | Crop field |
| 2B-P6 | Tubize | 49.4655096,5.1249068 | Crop field |
| 2B-P7 | Tubize | 49.4655096,5.1249068 | Crop field |
| 2B-P8 | Tubize | 49.4655096,5.1249068 | Crop field |
| 2B-P9 | Tubize | 49.4655096,5.1249068 | Crop field |
| 3B-P1 | Rebecq | 49.4655096,5.1249068 | Crop field |
| 3B-P2 | Rebecq | 49.4655096,5.1249068 | Crop field |
| 3B-P3 | Rebecq | 49.4655096,5.1249068 | Crop field |
| 3B-P4 | Rebecq | 49.4655096,5.1249068 | Crop field |
| 3B-P5 | Rebecq | 49.4655096,5.1249068 | Crop field |
| 3B-P6 | Rebecq | 49.4655096,5.1249068 | Crop field |
| 3B-P7 | Rebecq | 49.4655096,5.1249068 | Crop field |
| 3B-P8 | Rebecq | 49.4655096,5.1249068 | Crop field |
| 3B-P9 | Rebecq | 49.4655096,5.1249068 | Crop field |
| 4B-P1 | Perwez | 49.4655096,5.1249068 | Crop field |
| 4B-P2 | Perwez | 49.4655096,5.1249068 | Crop field |
| 4B-P3 | Perwez | 49.4655096,5.1249068 | Crop field |
| 4B-P4 | Perwez | 49.4655096,5.1249068 | Crop field |
| 4B-P5 | Perwez | 49.4655096,5.1249068 | Crop field |
| 4B-P6 | Perwez | 49.4655096,5.1249068 | Crop field |
| 4B-P7 | Perwez | 49.4655096,5.1249068 | Crop field |
| 4B-P8 | Perwez | 49.4655096,5.1249068 | Crop field |
| 4B-P9 | Perwez | 49.4655096,5.1249068 | Crop field |
| 5B-P1 | Incourt | 49.4655096,5.1249068 | Crop field |
| 5B-P2 | Incourt | 49.4655096,5.1249068 | Crop field |
| 5B-P3 | Incourt | 49.4655096,5.1249068 | Crop field |
| 5B-P4 | Incourt | 49.4655096,5.1249068 | Crop field |
| 5B-P5 | Incourt | 49.4655096,5.1249068 | Crop field |
| 5B-P6 | Incourt | 49.4655096,5.1249068 | Crop field |
| 5B-P7 | Incourt | 49.4655096,5.1249068 | Crop field |
| 5B-P8 | Incourt | 49.4655096,5.1249068 | Crop field |
| 5B-P9 | Incourt | 49.4655096,5.1249068 | Crop field |
| 6B-P1 | Incourt | 49.4655096,5.1249068 | Crop field |
| 6B-P2 | Incourt | 49.4655096,5.1249068 | Crop field |
| 6B-P3 | Incourt | 49.4655096,5.1249068 | Crop field |
| 6B-P4 | Incourt | 49.4655096,5.1249068 | Crop field |
| 6B-P5 | Incourt | 49.4655096,5.1249068 | Crop field |
| 6B-P6 | Incourt | 49.4655096,5.1249068 | Crop field |
| 6B-P7 | Incourt | 49.4655096,5.1249068 | Crop field |
| 6B-P8 | Incourt | 49.4655096,5.1249068 | Crop field |
| 6B-P9 | Incourt | 49.4655096,5.1249068 | Crop field |
| 7B-P1 | Gembloux | 49.4655096,5.1249068 | Crop field |
| 7B-P2 | Gembloux | 49.4655096,5.1249068 | Crop field |
| 7B-P3 | Gembloux | 49.4655096,5.1249068 | Crop field |
| 7B-P4 | Gembloux | 49.4655096,5.1249068 | Crop field |
| 7B-P5 | Gembloux | 49.4655096,5.1249068 | Crop field |
| 7B-P6 | Gembloux | 49.4655096,5.1249068 | Crop field |
| 7B-P7 | Gembloux | 49.4655096,5.1249068 | Crop field |
| 7B-P8 | Gembloux | 49.4655096,5.1249068 | Crop field |
| 7B-P9 | Gembloux | 49.4655096,5.1249068 | Crop field |
| 8B-P1 | Gembloux | 49.4655096,5.1249068 | Crop field |
| 8B-P2 | Gembloux | 49.4655096,5.1249068 | Crop field |
| 8B-P3 | Gembloux | 49.4655096,5.1249068 | Crop field |
| 8B-P4 | Gembloux | 49.4655096,5.1249068 | Crop field |
| 8B-P5 | Gembloux | 49.4655096,5.1249068 | Crop field |
| 8B-P6 | Gembloux | 49.4655096,5.1249068 | Crop field |
| 8B-P7 | Gembloux | 49.4655096,5.1249068 | Crop field |
| 8B-P8 | Gembloux | 49.4655096,5.1249068 | Crop field |
| 8B-P9 | Gembloux | 49.4655096,5.1249068 | Crop field |
| 9B-P1 | Gembloux | 49.4655096,5.1249068 | Crop field |
| 9B-P2 | Gembloux | 49.4655096,5.1249068 | Crop field |
| 9B-P3 | Gembloux | 49.4655096,5.1249068 | Crop field |
| 9B-P4 | Gembloux | 49.4655096,5.1249068 | Crop field |
| 9B-P5 | Gembloux | 49.4655096,5.1249068 | Crop field |
| 9B-P6 | Gembloux | 49.4655096,5.1249068 | Crop field |
| 9B-P7 | Gembloux | 49.4655096,5.1249068 | Crop field |
| 9B-P8 | Gembloux | 49.4655096,5.1249068 | Crop field |
| 9B-P9 | Gembloux | 49.4655096,5.1249068 | Crop field |
| 10B-P1 | Gembloux | 49.4655096,5.1249068 | Crop field |
| 10B-P2 | Gembloux | 49.4655096,5.1249068 | Crop field |
| 10B-P3 | Gembloux | 49.4655096,5.1249068 | Crop field |
| 10B-P4 | Gembloux | 49.4655096,5.1249068 | Crop field |
| 10B-P5 | Gembloux | 49.4655096,5.1249068 | Crop field |
| 10B-P6 | Gembloux | 49.4655096,5.1249068 | Crop field |
| 10B-P7 | Gembloux | 49.4655096,5.1249068 | Crop field |
| 10B-P8 | Gembloux | 49.4655096,5.1249068 | Crop field |
| 10B-P9 | Gembloux | 49.4655096,5.1249068 | Crop field |
| 11B-P1 | Gembloux | 49.4655096,5.1249068 | Crop field |
| 11B-P2 | Gembloux | 49.4655096,5.1249068 | Crop field |
| 11B-P3 | Gembloux | 49.4655096,5.1249068 | Crop field |
| 11B-P4 | Gembloux | 49.4655096,5.1249068 | Crop field |
| 11B-P5 | Gembloux | 49.4655096,5.1249068 | Crop field |
| 11B-P6 | Gembloux | 49.4655096,5.1249068 | Crop field |
| 11B-P7 | Gembloux | 49.4655096,5.1249068 | Crop field |
| 11B-P8 | Gembloux | 49.4655096,5.1249068 | Crop field |
| 11B-P9 | Gembloux | 49.4655096,5.1249068 | Crop field |
| 12B-P1 | Gembloux | 49.4655096,5.1249068 | Crop field |
| 12B-P2 | Gembloux | 49.4655096,5.1249068 | Crop field |
| 12B-P3 | Gembloux | 49.4655096,5.1249068 | Crop field |
| 12B-P4 | Gembloux | 49.4655096,5.1249068 | Crop field |
| 12B-P5 | Gembloux | 49.4655096,5.1249068 | Crop field |
| 12B-P6 | Gembloux | 49.4655096,5.1249068 | Crop field |
| 12B-P7 | Gembloux | 49.4655096,5.1249068 | Crop field |
| 12B-P8 | Gembloux | 49.4655096,5.1249068 | Crop field |
| 12B-P9 | Gembloux | 49.4655096,5.1249068 | Crop field |
| 13B-P1 | Gembloux | 49.4655096,5.1249068 | Crop field |
| 13B-P2 | Gembloux | 49.4655096,5.1249068 | Crop field |
| 13B-P3 | Gembloux | 49.4655096,5.1249068 | Crop field |
| 13B-P4 | Gembloux | 49.4655096,5.1249068 | Crop field |
| 13B-P5 | Gembloux | 49.4655096,5.1249068 | Crop field |
| 13B-P6 | Gembloux | 49.4655096,5.1249068 | Crop field |
| 13B-P7 | Gembloux | 49.4655096,5.1249068 | Crop field |
| 13B-P8 | Gembloux | 49.4655096,5.1249068 | Crop field |
| 13B-P9 | Gembloux | 49.4655096,5.1249068 | Crop field |
| 14B-P1 | Cognelée | 49.4655096,5.1249068 | Crop field |
| 14B-P2 | Cognelée | 49.4655096,5.1249068 | Crop field |
| 14B-P3 | Cognelée | 49.4655096,5.1249068 | Crop field |
| 14B-P4 | Cognelée | 49.4655096,5.1249068 | Crop field |
| 14B-P5 | Cognelée | 49.4655096,5.1249068 | Crop field |
| 14B-P6 | Cognelée | 49.4655096,5.1249068 | Crop field |
| 14B-P7 | Cognelée | 49.4655096,5.1249068 | Crop field |
| 14B-P8 | Cognelée | 49.4655096,5.1249068 | Crop field |
| 14B-P9 | Cognelée | 49.4655096,5.1249068 | Crop field |
| 15B-P1 | Bousval | 49.4655096,5.1249068 | Crop field |
| 15B-P2 | Bousval | 49.4655096,5.1249068 | Crop field |
| 15B-P3 | Bousval | 49.4655096,5.1249068 | Crop field |
| 15B-P4 | Bousval | 49.4655096,5.1249068 | Crop field |
| 15B-P5 | Bousval | 49.4655096,5.1249068 | Crop field |
| 15B-P6 | Bousval | 49.4655096,5.1249068 | Crop field |
| 15B-P7 | Bousval | 49.4655096,5.1249068 | Crop field |
| 15B-P8 | Bousval | 49.4655096,5.1249068 | Crop field |
| 15B-P9 | Bousval | 49.4655096,5.1249068 | Crop field |
| 16B-P1 | Court st Etienne | 49.4655096,5.1249068 | Crop field |
| 16B-P2 | Court st Etienne | 49.4655096,5.1249068 | Crop field |
| 16B-P3 | Court st Etienne | 49.4655096,5.1249068 | Crop field |
| 16B-P4 | Court st Etienne | 49.4655096,5.1249068 | Crop field |
| 16B-P5 | Court st Etienne | 49.4655096,5.1249068 | Crop field |
| 16B-P6 | Court st Etienne | 49.4655096,5.1249068 | Crop field |
| 16B-P7 | Court st Etienne | 49.4655096,5.1249068 | Crop field |
| 16B-P8 | Court st Etienne | 49.4655096,5.1249068 | Crop field |
| 16B-P9 | Court st Etienne | 49.4655096,5.1249068 | Crop field |
| 17B-P1 | Court st Etienne | 49.4655096,5.1249068 | Crop field |
| 17B-P2 | Court st Etienne | 49.4655096,5.1249068 | Crop field |
| 17B-P3 | Court st Etienne | 49.4655096,5.1249068 | Crop field |
| 17B-P4 | Court st Etienne | 49.4655096,5.1249068 | Crop field |
| 17B-P5 | Court st Etienne | 49.4655096,5.1249068 | Crop field |
| 17B-P6 | Court st Etienne | 49.4655096,5.1249068 | Crop field |
| 17B-P7 | Court st Etienne | 49.4655096,5.1249068 | Crop field |
| 17B-P8 | Court st Etienne | 49.4655096,5.1249068 | Crop field |
| 17B-P9 | Court st Etienne | 49.4655096,5.1249068 | Crop field |
| 18B-P1 | Louvain-la-Neuve | 49.4655096,5.1249068 | Crop field |
| 18B-P2 | Louvain-la-Neuve | 49.4655096,5.1249068 | Crop field |
| 18B-P3 | Louvain-la-Neuve | 49.4655096,5.1249068 | Crop field |
| 18B-P4 | Louvain-la-Neuve | 49.4655096,5.1249068 | Crop field |
| 18B-P5 | Louvain-la-Neuve | 49.4655096,5.1249068 | Crop field |
| 18B-P6 | Louvain-la-Neuve | 49.4655096,5.1249068 | Crop field |
| 18B-P7 | Louvain-la-Neuve | 49.4655096,5.1249068 | Crop field |
| 18B-P8 | Louvain-la-Neuve | 49.4655096,5.1249068 | Crop field |
| 18B-P9 | Louvain-la-Neuve | 49.4655096,5.1249068 | Crop field |
| 19B-P1 | Coxyde | 49.4655096,5.1249068 | Crop field |
| 19B-P2 | Coxyde | 49.4655096,5.1249068 | Crop field |
| 19B-P3 | Coxyde | 49.4655096,5.1249068 | Crop field |
| 19B-P4 | Coxyde | 49.4655096,5.1249068 | Crop field |
| 19B-P5 | Coxyde | 49.4655096,5.1249068 | Crop field |
| 19B-P6 | Coxyde | 49.4655096,5.1249068 | Crop field |
| 19B-P7 | Coxyde | 49.4655096,5.1249068 | Crop field |
| 19B-P8 | Coxyde | 49.4655096,5.1249068 | Crop field |
| 19B-P9 | Coxyde | 49.4655096,5.1249068 | Crop field |
| 20B-P1 | Coxyde | 49.4655096,5.1249068 | Crop field |
| 20B-P2 | Coxyde | 49.4655096,5.1249068 | Crop field |
| 20B-P3 | Coxyde | 49.4655096,5.1249068 | Crop field |
| 20B-P4 | Coxyde | 49.4655096,5.1249068 | Crop field |
| 20B-P5 | Coxyde | 49.4655096,5.1249068 | Crop field |
| 20B-P6 | Coxyde | 49.4655096,5.1249068 | Crop field |
| 20B-P7 | Coxyde | 49.4655096,5.1249068 | Crop field |
| 20B-P8 | Coxyde | 49.4655096,5.1249068 | Crop field |
| 20B-P9 | Coxyde | 49.4655096,5.1249068 | Crop field |
| 21B-P1 | Coxyde | 49.4655096,5.1249068 | Crop field |
| 21B-P2 | Coxyde | 49.4655096,5.1249068 | Crop field |
| 21B-P3 | Coxyde | 49.4655096,5.1249068 | Crop field |
| 21B-P4 | Coxyde | 49.4655096,5.1249068 | Crop field |
| 21B-P5 | Coxyde | 49.4655096,5.1249068 | Crop field |
| 21B-P6 | Coxyde | 49.4655096,5.1249068 | Crop field |
| 21B-P7 | Coxyde | 49.4655096,5.1249068 | Crop field |
| 21B-P8 | Coxyde | 49.4655096,5.1249068 | Crop field |
| 21B-P9 | Coxyde | 49.4655096,5.1249068 | Crop field |
| 22B-P1 | Furne | 49.4655096,5.1249068 | Crop field |
| 22B-P2 | Furne | 49.4655096,5.1249068 | Crop field |
| 22B-P3 | Furne | 49.4655096,5.1249068 | Crop field |
| 22B-P4 | Furne | 49.4655096,5.1249068 | Crop field |
| 22B-P5 | Furne | 49.4655096,5.1249068 | Crop field |
| 22B-P6 | Furne | 49.4655096,5.1249068 | Crop field |
| 22B-P7 | Furne | 49.4655096,5.1249068 | Crop field |
| 22B-P8 | Furne | 49.4655096,5.1249068 | Crop field |
| 22B-P9 | Furne | 49.4655096,5.1249068 | Crop field |
| 23B-P1 | Furne | 49.4655096,5.1249068 | Crop field |
| 23B-P2 | Furne | 49.4655096,5.1249068 | Crop field |
| 23B-P3 | Furne | 49.4655096,5.1249068 | Crop field |
| 23B-P4 | Furne | 49.4655096,5.1249068 | Crop field |
| 23B-P5 | Furne | 49.4655096,5.1249068 | Crop field |
| 23B-P6 | Furne | 49.4655096,5.1249068 | Crop field |
| 23B-P7 | Furne | 49.4655096,5.1249068 | Crop field |
| 23B-P8 | Furne | 49.4655096,5.1249068 | Crop field |
| 23B-P9 | Furne | 49.4655096,5.1249068 | Crop field |
| 24B-P1 | Furne | 49.4655096,5.1249068 | Crop field |
| 24B-P2 | Furne | 49.4655096,5.1249068 | Crop field |
| 24B-P3 | Furne | 49.4655096,5.1249068 | Crop field |
| 24B-P4 | Furne | 49.4655096,5.1249068 | Crop field |
| 24B-P5 | Furne | 49.4655096,5.1249068 | Crop field |
| 24B-P6 | Furne | 49.4655096,5.1249068 | Crop field |
| 24B-P7 | Furne | 49.4655096,5.1249068 | Crop field |
| 24B-P8 | Furne | 49.4655096,5.1249068 | Crop field |
| 24B-P9 | Furne | 49.4655096,5.1249068 | Crop field |
| 25B-P1 | Furne | 49.4655096,5.1249068 | Crop field |
| 25B-P2 | Furne | 49.4655096,5.1249068 | Crop field |
| 25B-P3 | Furne | 49.4655096,5.1249068 | Crop field |
| 25B-P4 | Furne | 49.4655096,5.1249068 | Crop field |
| 25B-P5 | Furne | 49.4655096,5.1249068 | Crop field |
| 25B-P6 | Furne | 49.4655096,5.1249068 | Crop field |
| 25B-P7 | Furne | 49.4655096,5.1249068 | Crop field |
| 25B-P8 | Furne | 49.4655096,5.1249068 | Crop field |
| 25B-P9 | Furne | 49.4655096,5.1249068 | Crop field |
| 26B-P1 | Furne | 49.4655096,5.1249068 | Crop field |
| 26B-P2 | Furne | 49.4655096,5.1249068 | Crop field |
| 26B-P3 | Furne | 49.4655096,5.1249068 | Crop field |
| 26B-P4 | Furne | 49.4655096,5.1249068 | Crop field |
| 26B-P5 | Furne | 49.4655096,5.1249068 | Crop field |
| 26B-P6 | Furne | 49.4655096,5.1249068 | Crop field |
| 26B-P7 | Furne | 49.4655096,5.1249068 | Crop field |
| 26B-P8 | Furne | 49.4655096,5.1249068 | Crop field |
| 26B-P9 | Furne | 49.4655096,5.1249068 | Crop field |
| 27B-P1 | Waremme | n/a | Crop field |
| 27B-P2 | Waremme | n/a | Crop field |
| 27B-P3 | Waremme | n/a | Crop field |
| 27B-P4 | Waremme | n/a | Crop field |
| 27B-P5 | Waremme | n/a | Crop field |
| 27B-P6 | Waremme | n/a | Crop field |
| 27B-P7 | Waremme | n/a | Crop field |
| 27B-P8 | Waremme | n/a | Crop field |
| 27B-P9 | Waremme | n/a | Crop field |
| 28B-P1 | Waremme | n/a | Crop field |
| 28B-P2 | Waremme | n/a | Crop field |
| 28B-P3 | Waremme | n/a | Crop field |
| 28B-P4 | Waremme | n/a | Crop field |
| 28B-P5 | Waremme | n/a | Crop field |
| 28B-P6 | Waremme | n/a | Crop field |
| 28B-P7 | Waremme | n/a | Crop field |
| 28B-P8 | Waremme | n/a | Crop field |
| 28B-P9 | Waremme | n/a | Crop field |
| 29B-P1 | Waremme | n/a | Crop field |
| 29B-P2 | Waremme | n/a | Crop field |
| 29B-P3 | Waremme | n/a | Crop field |
| 29B-P4 | Waremme | n/a | Crop field |
| 29B-P5 | Waremme | n/a | Crop field |
| 29B-P6 | Waremme | n/a | Crop field |
| 29B-P7 | Waremme | n/a | Crop field |
| 29B-P8 | Waremme | n/a | Crop field |
| 29B-P9 | Waremme | n/a | Crop field |
| 30B-P1 | Waremme | n/a | Crop field |
| 30B-P2 | Waremme | n/a | Crop field |
| 30B-P3 | Waremme | n/a | Crop field |
| 30B-P4 | Waremme | n/a | Crop field |
| 30B-P5 | Waremme | n/a | Crop field |
| 30B-P6 | Waremme | n/a | Crop field |
| 30B-P7 | Waremme | n/a | Crop field |
| 30B-P8 | Waremme | n/a | Crop field |
| 30B-P9 | Waremme | n/a | Crop field |
| 31B-P1 | Soumagne | 49.4655096,5.1249068 | Crop field |
| 31B-P2 | Soumagne | 49.4655096,5.1249068 | Crop field |
| 31B-P3 | Soumagne | 49.4655096,5.1249068 | Crop field |
| 31B-P4 | Soumagne | 49.4655096,5.1249068 | Crop field |
| 31B-P5 | Soumagne | 49.4655096,5.1249068 | Crop field |
| 31B-P6 | Soumagne | 49.4655096,5.1249068 | Crop field |
| 31B-P7 | Soumagne | 49.4655096,5.1249068 | Crop field |
| 31B-P8 | Soumagne | 49.4655096,5.1249068 | Crop field |
| 31B-P9 | Soumagne | 49.4655096,5.1249068 | Crop field |
| 32B-P1 | Soumagne | 49.4655096,5.1249068 | Crop field |
| 32B-P2 | Soumagne | 49.4655096,5.1249068 | Crop field |
| 32B-P3 | Soumagne | 49.4655096,5.1249068 | Crop field |
| 32B-P4 | Soumagne | 49.4655096,5.1249068 | Crop field |
| 32B-P5 | Soumagne | 49.4655096,5.1249068 | Crop field |
| 32B-P6 | Soumagne | 49.4655096,5.1249068 | Crop field |
| 32B-P7 | Soumagne | 49.4655096,5.1249068 | Crop field |
| 32B-P8 | Soumagne | 49.4655096,5.1249068 | Crop field |
| 32B-P9 | Soumagne | 49.4655096,5.1249068 | Crop field |
| 33B-P1 | Soumagne | 49.4655096,5.1249068 | Crop field |
| 33B-P2 | Soumagne | 49.4655096,5.1249068 | Crop field |
| 33B-P3 | Soumagne | 49.4655096,5.1249068 | Crop field |
| 33B-P4 | Soumagne | 49.4655096,5.1249068 | Crop field |
| 33B-P5 | Soumagne | 49.4655096,5.1249068 | Crop field |
| 33B-P6 | Soumagne | 49.4655096,5.1249068 | Crop field |
| 33B-P7 | Soumagne | 49.4655096,5.1249068 | Crop field |
| 33B-P8 | Soumagne | 49.4655096,5.1249068 | Crop field |
| 33B-P9 | Soumagne | 49.4655096,5.1249068 | Crop field |
| 34B-P1 | Soumagne | 49.4655096,5.1249068 | Crop field |
| 34B-P2 | Soumagne | 49.4655096,5.1249068 | Crop field |
| 34B-P3 | Soumagne | 49.4655096,5.1249068 | Crop field |
| 34B-P4 | Soumagne | 49.4655096,5.1249068 | Crop field |
| 34B-P5 | Soumagne | 49.4655096,5.1249068 | Crop field |
| 34B-P6 | Soumagne | 49.4655096,5.1249068 | Crop field |
| 34B-P7 | Soumagne | 49.4655096,5.1249068 | Crop field |
| 34B-P8 | Soumagne | 49.4655096,5.1249068 | Crop field |
| 34B-P9 | Soumagne | 49.4655096,5.1249068 | Crop field |
| 35B-P1 | Soumagne | 49.4655096,5.1249068 | Crop field |
| 35B-P2 | Soumagne | 49.4655096,5.1249068 | Crop field |
| 35B-P3 | Soumagne | 49.4655096,5.1249068 | Crop field |
| 35B-P4 | Soumagne | 49.4655096,5.1249068 | Crop field |
| 35B-P5 | Soumagne | 49.4655096,5.1249068 | Crop field |
| 35B-P6 | Soumagne | 49.4655096,5.1249068 | Crop field |
| 35B-P7 | Soumagne | 49.4655096,5.1249068 | Crop field |
| 35B-P8 | Soumagne | 49.4655096,5.1249068 | Crop field |
| 35B-P9 | Soumagne | 49.4655096,5.1249068 | Crop field |
| 36B-P1 | Rebecq | 49.4655096,5.1249068 | Crop field |
| 36B-P2 | Rebecq | 49.4655096,5.1249068 | Crop field |
| 36B-P3 | Rebecq | 49.4655096,5.1249068 | Crop field |
| 36B-P4 | Rebecq | 49.4655096,5.1249068 | Crop field |
| 36B-P5 | Rebecq | 49.4655096,5.1249068 | Crop field |
| 36B-P6 | Rebecq | 49.4655096,5.1249068 | Crop field |
| 36B-P7 | Rebecq | 49.4655096,5.1249068 | Crop field |
| 36B-P8 | Rebecq | 49.4655096,5.1249068 | Crop field |
| 36B-P9 | Rebecq | 49.4655096,5.1249068 | Crop field |
| 37B-P1 | Rebecq | 49.4655096,5.1249068 | Crop field |
| 37B-P2 | Rebecq | 49.4655096,5.1249068 | Crop field |
| 37B-P3 | Rebecq | 49.4655096,5.1249068 | Crop field |
| 37B-P4 | Rebecq | 49.4655096,5.1249068 | Crop field |
| 37B-P5 | Rebecq | 49.4655096,5.1249068 | Crop field |
| 37B-P6 | Rebecq | 49.4655096,5.1249068 | Crop field |
| 37B-P7 | Rebecq | 49.4655096,5.1249068 | Crop field |
| 37B-P8 | Rebecq | 49.4655096,5.1249068 | Crop field |
| 37B-P9 | Rebecq | 49.4655096,5.1249068 | Crop field |
| 38B-P1 | Rebecq | 49.4655096,5.1249068 | Crop field |
| 38B-P2 | Rebecq | 49.4655096,5.1249068 | Crop field |
| 38B-P3 | Rebecq | 49.4655096,5.1249068 | Crop field |
| 38B-P4 | Rebecq | 49.4655096,5.1249068 | Crop field |
| 38B-P5 | Rebecq | 49.4655096,5.1249068 | Crop field |
| 38B-P6 | Rebecq | 49.4655096,5.1249068 | Crop field |
| 38B-P7 | Rebecq | 49.4655096,5.1249068 | Crop field |
| 38B-P8 | Rebecq | 49.4655096,5.1249068 | Crop field |
| 38B-P9 | Rebecq | 49.4655096,5.1249068 | Crop field |
| 39B-P1 | Soignies | 49.4655096,5.1249068 | Crop field |
| 39B-P2 | Soignies | 49.4655096,5.1249068 | Crop field |
| 39B-P3 | Soignies | 49.4655096,5.1249068 | Crop field |
| 39B-P4 | Soignies | 49.4655096,5.1249068 | Crop field |
| 39B-P5 | Soignies | 49.4655096,5.1249068 | Crop field |
| 39B-P6 | Soignies | 49.4655096,5.1249068 | Crop field |
| 39B-P7 | Soignies | 49.4655096,5.1249068 | Crop field |
| 39B-P8 | Soignies | 49.4655096,5.1249068 | Crop field |
| 39B-P9 | Soignies | 49.4655096,5.1249068 | Crop field |
| 40B-P1 | Soignies | 49.4655096,5.1249068 | Crop field |
| 40B-P2 | Soignies | 49.4655096,5.1249068 | Crop field |
| 40B-P3 | Soignies | 49.4655096,5.1249068 | Crop field |
| 40B-P4 | Soignies | 49.4655096,5.1249068 | Crop field |
| 40B-P5 | Soignies | 49.4655096,5.1249068 | Crop field |
| 40B-P6 | Soignies | 49.4655096,5.1249068 | Crop field |
| 40B-P7 | Soignies | 49.4655096,5.1249068 | Crop field |
| 40B-P8 | Soignies | 49.4655096,5.1249068 | Crop field |
| 40B-P9 | Soignies | 49.4655096,5.1249068 | Crop field |
| 41B-P1 | Bertrix | 49.848617,5.239266 | Crop field |
| 41B-P2 | Bertrix | 49.848617,5.239266 | Crop field |
| 41B-P3 | Bertrix | 49.848617,5.239266 | Crop field |
| 41B-P4 | Bertrix | 49.848617,5.239266 | Crop field |
| 41B-P5 | Bertrix | 49.848617,5.239266 | Crop field |
| 41B-P6 | Bertrix | 49.848617,5.239266 | Crop field |
| 41B-P7 | Bertrix | 49.848617,5.239266 | Crop field |
| 41B-P8 | Bertrix | 49.848617,5.239266 | Crop field |
| 41B-P9 | Bertrix | 49.848617,5.239266 | Crop field |
| 42B-P1 | Bertrix | 49.85128,5.25257 | Crop field |
| 42B-P2 | Bertrix | 49.85128,5.25257 | Crop field |
| 42B-P3 | Bertrix | 49.85128,5.25257 | Crop field |
| 42B-P4 | Bertrix | 49.85128,5.25257 | Crop field |
| 42B-P5 | Bertrix | 49.85128,5.25257 | Crop field |
| 42B-P6 | Bertrix | 49.85128,5.25257 | Crop field |
| 42B-P7 | Bertrix | 49.85128,5.25257 | Crop field |
| 42B-P8 | Bertrix | 49.85128,5.25257 | Crop field |
| 42B-P9 | Bertrix | 49.85128,5.25257 | Crop field |
| 43B-P1 | Bertrix | 49.85128,5.25257 | Crop field |
| 43B-P2 | Bertrix | 49.85128,5.25257 | Crop field |
| 43B-P3 | Bertrix | 49.85128,5.25257 | Crop field |
| 43B-P4 | Bertrix | 49.85128,5.25257 | Crop field |
| 43B-P5 | Bertrix | 49.85128,5.25257 | Crop field |
| 43B-P6 | Bertrix | 49.85128,5.25257 | Crop field |
| 43B-P7 | Bertrix | 49.85128,5.25257 | Crop field |
| 43B-P8 | Bertrix | 49.85128,5.25257 | Crop field |
| 43B-P9 | Bertrix | 49.85128,5.25257 | Crop field |
| 44B-P1 | Bertrix | 49.84403,5.265884 | Crop field |
| 44B-P2 | Bertrix | 49.84403,5.265884 | Crop field |
| 44B-P3 | Bertrix | 49.84403,5.265884 | Crop field |
| 44B-P4 | Bertrix | 49.84403,5.265884 | Crop field |
| 44B-P5 | Bertrix | 49.84403,5.265884 | Crop field |
| 44B-P6 | Bertrix | 49.84403,5.265884 | Crop field |
| 44B-P7 | Bertrix | 49.84403,5.265884 | Crop field |
| 44B-P8 | Bertrix | 49.84403,5.265884 | Crop field |
| 44B-P9 | Bertrix | 49.84403,5.265884 | Crop field |
| 45B-P1 | Bertrix | 49.782615,5.213131 | Crop field |
| 45B-P2 | Bertrix | 49.782615,5.213131 | Crop field |
| 45B-P3 | Bertrix | 49.782615,5.213131 | Crop field |
| 45B-P4 | Bertrix | 49.782615,5.213131 | Crop field |
| 45B-P5 | Bertrix | 49.782615,5.213131 | Crop field |
| 45B-P6 | Bertrix | 49.782615,5.213131 | Crop field |
| 45B-P7 | Bertrix | 49.782615,5.213131 | Crop field |
| 45B-P8 | Bertrix | 49.782615,5.213131 | Crop field |
| 45B-P9 | Bertrix | 49.782615,5.213131 | Crop field |
| 46B-P1 | Ath | 50.645821,3.856544 | Crop field |
| 46B-P2 | Ath | 50.645821,3.856544 | Crop field |
| 46B-P3 | Ath | 50.645821,3.856544 | Crop field |
| 46B-P4 | Ath | 50.645821,3.856544 | Crop field |
| 46B-P5 | Ath | 50.645821,3.856544 | Crop field |
| 46B-P6 | Ath | 50.645821,3.856544 | Crop field |
| 46B-P7 | Ath | 50.645821,3.856544 | Crop field |
| 46B-P8 | Ath | 50.645821,3.856544 | Crop field |
| 46B-P9 | Ath | 50.645821,3.856544 | Crop field |
| 47B-P1 | Lens | 50.586615,3.906841 | Crop field |
| 47B-P2 | Lens | 50.586615,3.906841 | Crop field |
| 47B-P3 | Lens | 50.586615,3.906841 | Crop field |
| 47B-P4 | Lens | 50.586615,3.906841 | Crop field |
| 47B-P5 | Lens | 50.586615,3.906841 | Crop field |
| 47B-P6 | Lens | 50.586615,3.906841 | Crop field |
| 47B-P7 | Lens | 50.586615,3.906841 | Crop field |
| 47B-P8 | Lens | 50.586615,3.906841 | Crop field |
| 47B-P9 | Lens | 50.586615,3.906841 | Crop field |
| 48B-P1 | Soignies | 50.563978,4.017375 | Crop field |
| 48B-P2 | Soignies | 50.563978,4.017375 | Crop field |
| 48B-P3 | Soignies | 50.563978,4.017375 | Crop field |
| 48B-P4 | Soignies | 50.563978,4.017375 | Crop field |
| 48B-P5 | Soignies | 50.563978,4.017375 | Crop field |
| 48B-P6 | Soignies | 50.563978,4.017375 | Crop field |
| 48B-P7 | Soignies | 50.563978,4.017375 | Crop field |
| 48B-P8 | Soignies | 50.563978,4.017375 | Crop field |
| 48B-P9 | Soignies | 50.563978,4.017375 | Crop field |
| C1-P1 | Rebecq | n/a | Compost |
| C1-P2 | Rebecq | n/a | Compost |
| C1-P3 | Rebecq | n/a | Compost |
| C1-P4 | Rebecq | n/a | Compost |
| C1-P5 | Rebecq | n/a | Compost |
| C1-P6 | Rebecq | n/a | Compost |
| C1-P7 | Rebecq | n/a | Compost |
| C1-P8 | Rebecq | n/a | Compost |
| C1-P9 | Rebecq | n/a | Compost |
| C2-P1 | Bousval | n/a | Compost |
| C2-P2 | Bousval | n/a | Compost |
| C2-P3 | Bousval | n/a | Compost |
| C2-P4 | Bousval | n/a | Compost |
| C2-P5 | Bousval | n/a | Compost |
| C2-P6 | Bousval | n/a | Compost |
| C2-P7 | Bousval | n/a | Compost |
| C2-P8 | Bousval | n/a | Compost |
| C2-P9 | Bousval | n/a | Compost |
| C3-P1 | Ath | 51.076667, 4.427567 | Compost |
| C3-P2 | Ath | 51.076667, 4.427567 | Compost |
| C3-P3 | Ath | 51.076667, 4.427567 | Compost |
| C3-P4 | Ath | 51.076667, 4.427567 | Compost |
| C3-P5 | Ath | 51.076667, 4.427567 | Compost |
| C3-P6 | Ath | 51.076667, 4.427567 | Compost |
| C3-P7 | Ath | 51.076667, 4.427567 | Compost |
| C3-P8 | Ath | 51.076667, 4.427567 | Compost |
| C3-P9 | Ath | 51.076667, 4.427567 | Compost |
| C4-P1 | Habay | 49,73745, 5.61766 | Compost |
| C4-P2 | Habay | 49,73745, 5.61766 | Compost |
| C4-P3 | Habay | 49,73745, 5.61766 | Compost |
| C4-P4 | Habay | 49,73745, 5.61766 | Compost |
| C4-P5 | Habay | 49,73745, 5.61766 | Compost |
| C4-P6 | Habay | 49,73745, 5.61766 | Compost |
| C4-P7 | Habay | 49,73745, 5.61766 | Compost |
| C4-P8 | Habay | 49,73745, 5.61766 | Compost |
| C4-P9 | Habay | 49,73745, 5.61766 | Compost |
| F1-P1 | Rebecq | 50.647229,4.15516 | Manure |
| F1-P2 | Rebecq | 50.647229,4.15516 | Manure |
| F1-P3 | Rebecq | 50.647229,4.15516 | Manure |
| F1-P4 | Rebecq | 50.647229,4.15516 | Manure |
| F1-P5 | Rebecq | 50.647229,4.15516 | Manure |
| F1-P6 | Rebecq | 50.647229,4.15516 | Manure |
| F1-P7 | Rebecq | 50.647229,4.15516 | Manure |
| F1-P8 | Rebecq | 50.647229,4.15516 | Manure |
| F1-P9 | Rebecq | 50.647229,4.15516 | Manure |
| F2-P1 | Bertrix | 49.846932,5.238757 | Manure |
| F2-P2 | Bertrix | 49.846932,5.238757 | Manure |
| F2-P3 | Bertrix | 49.846932,5.238757 | Manure |
| F2-P4 | Bertrix | 49.846932,5.238757 | Manure |
| F2-P5 | Bertrix | 49.846932,5.238757 | Manure |
| F2-P6 | Bertrix | 49.846932,5.238757 | Manure |
| F2-P7 | Bertrix | 49.846932,5.238757 | Manure |
| F2-P8 | Bertrix | 49.846932,5.238757 | Manure |
| F2-P9 | Bertrix | 49.846932,5.238757 | Manure |
| F3-P1 | Bertrix | 49.846937,5.238751 | Manure |
| F3-P2 | Bertrix | 49.846937,5.238751 | Manure |
| F3-P3 | Bertrix | 49.846937,5.238751 | Manure |
| F3-P4 | Bertrix | 49.846937,5.238751 | Manure |
| F3-P5 | Bertrix | 49.846937,5.238751 | Manure |
| F3-P6 | Bertrix | 49.846937,5.238751 | Manure |
| F3-P7 | Bertrix | 49.846937,5.238751 | Manure |
| F3-P8 | Bertrix | 49.846937,5.238751 | Manure |
| F3-P9 | Bertrix | 49.846937,5.238751 | Manure |
| F4-P1 | Bertrix | 49.844075,5.265056 | Manure |
| F4-P2 | Bertrix | 49.844075,5.265056 | Manure |
| F4-P3 | Bertrix | 49.844075,5.265056 | Manure |
| F4-P4 | Bertrix | 49.844075,5.265056 | Manure |
| F4-P5 | Bertrix | 49.844075,5.265056 | Manure |
| F4-P6 | Bertrix | 49.844075,5.265056 | Manure |
| F4-P7 | Bertrix | 49.844075,5.265056 | Manure |
| F4-P8 | Bertrix | 49.844075,5.265056 | Manure |
| F4-P9 | Bertrix | 49.844075,5.265056 | Manure |
| F5-P1 | Bertrix | 49.782636,5.21363 | Manure |
| F5-P2 | Bertrix | 49.782636,5.21363 | Manure |
| F5-P3 | Bertrix | 49.782636,5.21363 | Manure |
| F5-P4 | Bertrix | 49.782636,5.21363 | Manure |
| F5-P5 | Bertrix | 49.782636,5.21363 | Manure |
| F5-P6 | Bertrix | 49.782636,5.21363 | Manure |
| F5-P7 | Bertrix | 49.782636,5.21363 | Manure |
| F5-P8 | Bertrix | 49.782636,5.21363 | Manure |
| F5-P9 | Bertrix | 49.782636,5.21363 | Manure |
| F6-P1 | Soignies | 50.56837, 4.01665 | Manure |
| F6-P2 | Soignies | 50.56837, 4.01665 | Manure |
| F6-P3 | Soignies | 50.56837, 4.01665 | Manure |
| F6-P4 | Soignies | 50.56837, 4.01665 | Manure |
| F6-P5 | Soignies | 50.56837, 4.01665 | Manure |
| F6-P6 | Soignies | 50.56837, 4.01665 | Manure |
| F6-P7 | Soignies | 50.56837, 4.01665 | Manure |
| F6-P8 | Soignies | 50.56837, 4.01665 | Manure |
| F6-P9 | Soignies | 50.56837, 4.01665 | Manure |
| F7-P1 | Brugelette | 50.59957, 3.839467 | Manure |
| F7-P2 | Brugelette | 50.59957, 3.839467 | Manure |
| F7-P3 | Brugelette | 50.59957, 3.839467 | Manure |
| F7-P4 | Brugelette | 50.59957, 3.839467 | Manure |
| F7-P5 | Brugelette | 50.59957, 3.839467 | Manure |
| F7-P6 | Brugelette | 50.59957, 3.839467 | Manure |
| F7-P7 | Brugelette | 50.59957, 3.839467 | Manure |
| F7-P8 | Brugelette | 50.59957, 3.839467 | Manure |
| F7-P9 | Brugelette | 50.59957, 3.839467 | Manure |
| F8-P1 | Habay | 49.739067, 5.615467 | Manure |
| F8-P2 | Habay | 49.739067, 5.615467 | Manure |
| F8-P3 | Habay | 49.739067, 5.615467 | Manure |
| F8-P4 | Habay | 49.739067, 5.615467 | Manure |
| F8-P5 | Habay | 49.739067, 5.615467 | Manure |
| F8-P6 | Habay | 49.739067, 5.615467 | Manure |
| F8-P7 | Habay | 49.739067, 5.615467 | Manure |
| F8-P8 | Habay | 49.739067, 5.615467 | Manure |
| F8-P9 | Habay | 49.739067, 5.615467 | Manure |
| 41R-P1 | Bertrix | 4.95054348, 5.142126 | Potato root |
| 41R-P2 | Bertrix | 4.95054348, 5.142126 | Potato root |
| 41R-P3 | Bertrix | 4.95054348, 5.142126 | Potato root |
| 41R-P4 | Bertrix | 4.95054348, 5.142126 | Potato root |
| 41R-P5 | Bertrix | 4.95054348, 5.142126 | Potato root |
| 41R-P6 | Bertrix | 4.95054348, 5.142126 | Potato root |
| 41R-P7 | Bertrix | 4.95054348, 5.142126 | Potato root |
| 41R-P8 | Bertrix | 4.95054348, 5.142126 | Potato root |
| 41R-P9 | Bertrix | 4.95054348, 5.142126 | Potato root |
| 41L-P1 | Bertrix | 4.95054348, 5.142126 | Potato leaf |
| 41L-P2 | Bertrix | 4.95054348, 5.142126 | Potato leaf |
| 41L-P3 | Bertrix | 4.95054348, 5.142126 | Potato leaf |
| 41L-P4 | Bertrix | 4.95054348, 5.142126 | Potato leaf |
| 41L-P5 | Bertrix | 4.95054348, 5.142126 | Potato leaf |
| 41L-P6 | Bertrix | 4.95054348, 5.142126 | Potato leaf |
| 41L-P7 | Bertrix | 4.95054348, 5.142126 | Potato leaf |
| 41L-P8 | Bertrix | 4.95054348, 5.142126 | Potato leaf |
| 41L-P9 | Bertrix | 4.95054348, 5.142126 | Potato leaf |
| 41T-P1 | Bertrix | 4.95054348, 5.142126 | Potato tuber |
| 41T-P2 | Bertrix | 4.95054348, 5.142126 | Potato tuber |
| 41T-P3 | Bertrix | 4.95054348, 5.142126 | Potato tuber |
| 41T-P4 | Bertrix | 4.95054348, 5.142126 | Potato tuber |
| 41T-P5 | Bertrix | 4.95054348, 5.142126 | Potato tuber |
| 41T-P6 | Bertrix | 4.95054348, 5.142126 | Potato tuber |
| 41T-P7 | Bertrix | 4.95054348, 5.142126 | Potato tuber |
| 41T-P8 | Bertrix | 4.95054348, 5.142126 | Potato tuber |
| 41T-P9 | Bertrix | 4.95054348, 5.142126 | Potato tuber |
| 42R-P1 | Bertrix | 49.4655096,5.1249068 | Potato root |
| 42R-P2 | Bertrix | 49.4655096,5.1249068 | Potato root |
| 42R-P3 | Bertrix | 49.4655096,5.1249068 | Potato root |
| 42R-P4 | Bertrix | 49.4655096,5.1249068 | Potato root |
| 42R-P5 | Bertrix | 49.4655096,5.1249068 | Potato root |
| 42R-P6 | Bertrix | 49.4655096,5.1249068 | Potato root |
| 42R-P7 | Bertrix | 49.4655096,5.1249068 | Potato root |
| 42R-P8 | Bertrix | 49.4655096,5.1249068 | Potato root |
| 42R-P9 | Bertrix | 49.4655096,5.1249068 | Potato root |
| 42L-P1 | Bertrix | 49.4655096,5.1249068 | Potato leaf |
| 42L-P2 | Bertrix | 49.4655096,5.1249068 | Potato leaf |
| 42L-P3 | Bertrix | 49.4655096,5.1249068 | Potato leaf |
| 42L-P4 | Bertrix | 49.4655096,5.1249068 | Potato leaf |
| 42L-P5 | Bertrix | 49.4655096,5.1249068 | Potato leaf |
| 42L-P6 | Bertrix | 49.4655096,5.1249068 | Potato leaf |
| 42L-P7 | Bertrix | 49.4655096,5.1249068 | Potato leaf |
| 42L-P8 | Bertrix | 49.4655096,5.1249068 | Potato leaf |
| 42L-P9 | Bertrix | 49.4655096,5.1249068 | Potato leaf |
| 42T-P1 | Bertrix | 49.4655096,5.1249068 | Potato tuber |
| 42T-P2 | Bertrix | 49.4655096,5.1249068 | Potato tuber |
| 42T-P3 | Bertrix | 49.4655096,5.1249068 | Potato tuber |
| 42T-P4 | Bertrix | 49.4655096,5.1249068 | Potato tuber |
| 42T-P5 | Bertrix | 49.4655096,5.1249068 | Potato tuber |
| 42T-P6 | Bertrix | 49.4655096,5.1249068 | Potato tuber |
| 42T-P7 | Bertrix | 49.4655096,5.1249068 | Potato tuber |
| 42T-P8 | Bertrix | 49.4655096,5.1249068 | Potato tuber |
| 42T-P9 | Bertrix | 49.4655096,5.1249068 | Potato tuber |
| 43R-P3 | Bertrix | 49.4655096,5.1249068 | Potato root |
| 43R-P4 | Bertrix | 49.4655096,5.1249068 | Potato root |
| 43R-P3 | Bertrix | 49.4655096,5.1249068 | Potato root |
| 43R-P4 | Bertrix | 49.4655096,5.1249068 | Potato root |
| 43R-P5 | Bertrix | 49.4655096,5.1249068 | Potato root |
| 43R-P6 | Bertrix | 49.4655096,5.1249068 | Potato root |
| 43R-P7 | Bertrix | 49.4655096,5.1249068 | Potato root |
| 43R-P8 | Bertrix | 49.4655096,5.1249068 | Potato root |
| 43R-P9 | Bertrix | 49.4655096,5.1249068 | Potato root |
| 43L-P3 | Bertrix | 49.4655096,5.1249068 | Potato leaf |
| 43L-P4 | Bertrix | 49.4655096,5.1249068 | Potato leaf |
| 43L-P3 | Bertrix | 49.4655096,5.1249068 | Potato leaf |
| 43L-P4 | Bertrix | 49.4655096,5.1249068 | Potato leaf |
| 43L-P5 | Bertrix | 49.4655096,5.1249068 | Potato leaf |
| 43L-P6 | Bertrix | 49.4655096,5.1249068 | Potato leaf |
| 43L-P7 | Bertrix | 49.4655096,5.1249068 | Potato leaf |
| 43L-P8 | Bertrix | 49.4655096,5.1249068 | Potato leaf |
| 43L-P9 | Bertrix | 49.4655096,5.1249068 | Potato leaf |
| 43T-P3 | Bertrix | 49.4655096,5.1249068 | Potato tuber |
| 43T-P4 | Bertrix | 49.4655096,5.1249068 | Potato tuber |
| 43T-P3 | Bertrix | 49.4655096,5.1249068 | Potato tuber |
| 43T-P4 | Bertrix | 49.4655096,5.1249068 | Potato tuber |
| 43T-P5 | Bertrix | 49.4655096,5.1249068 | Potato tuber |
| 43T-P6 | Bertrix | 49.4655096,5.1249068 | Potato tuber |
| 43T-P7 | Bertrix | 49.4655096,5.1249068 | Potato tuber |
| 43T-P8 | Bertrix | 49.4655096,5.1249068 | Potato tuber |
| 43T-P9 | Bertrix | 49.4655096,5.1249068 | Potato tuber |
| 44R-P3 | Bertrix | 49.4655096,5.1249068 | Potato root |
| 44R-P4 | Bertrix | 49.4655096,5.1249068 | Potato root |
| 44R-P3 | Bertrix | 49.4655096,5.1249068 | Potato root |
| 44R-P4 | Bertrix | 49.4655096,5.1249068 | Potato root |
| 44R-P5 | Bertrix | 49.4655096,5.1249068 | Potato root |
| 44R-P6 | Bertrix | 49.4655096,5.1249068 | Potato root |
| 44R-P7 | Bertrix | 49.4655096,5.1249068 | Potato root |
| 44R-P8 | Bertrix | 49.4655096,5.1249068 | Potato root |
| 44R-P9 | Bertrix | 49.4655096,5.1249068 | Potato root |
| 44L-P3 | Bertrix | 49.4655096,5.1249068 | Potato leaf |
| 44L-P4 | Bertrix | 49.4655096,5.1249068 | Potato leaf |
| 44L-P3 | Bertrix | 49.4655096,5.1249068 | Potato leaf |
| 44L-P4 | Bertrix | 49.4655096,5.1249068 | Potato leaf |
| 44L-P5 | Bertrix | 49.4655096,5.1249068 | Potato leaf |
| 44L-P6 | Bertrix | 49.4655096,5.1249068 | Potato leaf |
| 44L-P7 | Bertrix | 49.4655096,5.1249068 | Potato leaf |
| 44L-P8 | Bertrix | 49.4655096,5.1249068 | Potato leaf |
| 44L-P9 | Bertrix | 49.4655096,5.1249068 | Potato leaf |
| 44T-P3 | Bertrix | 49.4655096,5.1249068 | Potato tuber |
| 44T-P4 | Bertrix | 49.4655096,5.1249068 | Potato tuber |
| 44T-P3 | Bertrix | 49.4655096,5.1249068 | Potato tuber |
| 44T-P4 | Bertrix | 49.4655096,5.1249068 | Potato tuber |
| 44T-P5 | Bertrix | 49.4655096,5.1249068 | Potato tuber |
| 44T-P6 | Bertrix | 49.4655096,5.1249068 | Potato tuber |
| 44T-P7 | Bertrix | 49.4655096,5.1249068 | Potato tuber |
| 44T-P8 | Bertrix | 49.4655096,5.1249068 | Potato tuber |
| 44T-P9 | Bertrix | 49.4655096,5.1249068 | Potato tuber |
| 45R-P1 | Bertrix | 49.4655096,5.1249068 | Potato root |
| 45R-P2 | Bertrix | 49.4655096,5.1249068 | Potato root |
| 45R-P3 | Bertrix | 49.4655096,5.1249068 | Potato root |
| 45R-P4 | Bertrix | 49.4655096,5.1249068 | Potato root |
| 45R-P5 | Bertrix | 49.4655096,5.1249068 | Potato root |
| 45R-P6 | Bertrix | 49.4655096,5.1249068 | Potato root |
| 45R-P7 | Bertrix | 49.4655096,5.1249068 | Potato root |
| 45R-P8 | Bertrix | 49.4655096,5.1249068 | Potato root |
| 45R-P9 | Bertrix | 49.4655096,5.1249068 | Potato root |
| 45L-P1 | Bertrix | 49.4655096,5.1249068 | Potato leaf |
| 45L-P2 | Bertrix | 49.4655096,5.1249068 | Potato leaf |
| 45L-P3 | Bertrix | 49.4655096,5.1249068 | Potato leaf |
| 45L-P4 | Bertrix | 49.4655096,5.1249068 | Potato leaf |
| 45L-P5 | Bertrix | 49.4655096,5.1249068 | Potato leaf |
| 45L-P6 | Bertrix | 49.4655096,5.1249068 | Potato leaf |
| 45L-P7 | Bertrix | 49.4655096,5.1249068 | Potato leaf |
| 45L-P8 | Bertrix | 49.4655096,5.1249068 | Potato leaf |
| 45L-P9 | Bertrix | 49.4655096,5.1249068 | Potato leaf |
| 45T-P1 | Bertrix | 49.4655096,5.1249068 | Potato tuber |
| 45T-P2 | Bertrix | 49.4655096,5.1249068 | Potato tuber |
| 45T-P3 | Bertrix | 49.4655096,5.1249068 | Potato tuber |
| 45T-P4 | Bertrix | 49.4655096,5.1249068 | Potato tuber |
| 45T-P5 | Bertrix | 49.4655096,5.1249068 | Potato tuber |
| 45T-P6 | Bertrix | 49.4655096,5.1249068 | Potato tuber |
| 45T-P7 | Bertrix | 49.4655096,5.1249068 | Potato tuber |
| 45T-P8 | Bertrix | 49.4655096,5.1249068 | Potato tuber |
| 45T-P9 | Bertrix | 49.4655096,5.1249068 | Potato tuber |
| 46R-P1 | Ath | 50.645821,3.856544 | Potato root |
| 46R-P2 | Ath | 50.645821,3.856544 | Potato root |
| 46R-P3 | Ath | 50.645821,3.856544 | Potato root |
| 46R-P4 | Ath | 50.645821,3.856544 | Potato root |
| 46R-P5 | Ath | 50.645821,3.856544 | Potato root |
| 46R-P6 | Ath | 50.645821,3.856544 | Potato root |
| 46R-P7 | Ath | 50.645821,3.856544 | Potato root |
| 46R-P8 | Ath | 50.645821,3.856544 | Potato root |
| 46R-P9 | Ath | 50.645821,3.856544 | Potato root |
| 46L-P1 | Ath | 50.645821,3.856544 | Potato leaf |
| 46L-P2 | Ath | 50.645821,3.856544 | Potato leaf |
| 46L-P3 | Ath | 50.645821,3.856544 | Potato leaf |
| 46L-P4 | Ath | 50.645821,3.856544 | Potato leaf |
| 46L-P5 | Ath | 50.645821,3.856544 | Potato leaf |
| 46L-P6 | Ath | 50.645821,3.856544 | Potato leaf |
| 46L-P7 | Ath | 50.645821,3.856544 | Potato leaf |
| 46L-P8 | Ath | 50.645821,3.856544 | Potato leaf |
| 46L-P9 | Ath | 50.645821,3.856544 | Potato leaf |
| 46T-P1 | Ath | 50.645821,3.856544 | Potato tuber |
| 46T-P2 | Ath | 50.645821,3.856544 | Potato tuber |
| 46T-P3 | Ath | 50.645821,3.856544 | Potato tuber |
| 46T-P4 | Ath | 50.645821,3.856544 | Potato tuber |
| 46T-P5 | Ath | 50.645821,3.856544 | Potato tuber |
| 46T-P6 | Ath | 50.645821,3.856544 | Potato tuber |
| 46T-P7 | Ath | 50.645821,3.856544 | Potato tuber |
| 46T-P8 | Ath | 50.645821,3.856544 | Potato tuber |
| 46T-P9 | Ath | 50.645821,3.856544 | Potato tuber |
| 47R-P1 | Lens | 50.586615,3.906841 | Potato root |
| 47R-P2 | Lens | 50.586615,3.906841 | Potato root |
| 47R-P3 | Lens | 50.586615,3.906841 | Potato root |
| 47R-P4 | Lens | 50.586615,3.906841 | Potato root |
| 47R-P5 | Lens | 50.586615,3.906841 | Potato root |
| 47R-P6 | Lens | 50.586615,3.906841 | Potato root |
| 47R-P7 | Lens | 50.586615,3.906841 | Potato root |
| 47R-P8 | Lens | 50.586615,3.906841 | Potato root |
| 47R-P9 | Lens | 50.586615,3.906841 | Potato root |
| 47L-P1 | Lens | 50.586615,3.906841 | Potato leaf |
| 47L-P2 | Lens | 50.586615,3.906841 | Potato leaf |
| 47L-P3 | Lens | 50.586615,3.906841 | Potato leaf |
| 47L-P4 | Lens | 50.586615,3.906841 | Potato leaf |
| 47L-P5 | Lens | 50.586615,3.906841 | Potato leaf |
| 47L-P6 | Lens | 50.586615,3.906841 | Potato leaf |
| 47L-P7 | Lens | 50.586615,3.906841 | Potato leaf |
| 47L-P8 | Lens | 50.586615,3.906841 | Potato leaf |
| 47L-P9 | Lens | 50.586615,3.906841 | Potato leaf |
| 47T-P1 | Lens | 50.586615,3.906841 | Potato tuber |
| 47T-P2 | Lens | 50.586615,3.906841 | Potato tuber |
| 47T-P3 | Lens | 50.586615,3.906841 | Potato tuber |
| 47T-P4 | Lens | 50.586615,3.906841 | Potato tuber |
| 47T-P5 | Lens | 50.586615,3.906841 | Potato tuber |
| 47T-P6 | Lens | 50.586615,3.906841 | Potato tuber |
| 47T-P7 | Lens | 50.586615,3.906841 | Potato tuber |
| 47T-P8 | Lens | 50.586615,3.906841 | Potato tuber |
| 47T-P9 | Lens | 50.586615,3.906841 | Potato tuber |
| 48R-P1 | Soignies | 50.563978,4.017375 | Potato root |
| 48R-P2 | Soignies | 50.563978,4.017375 | Potato root |
| 48R-P3 | Soignies | 50.563978,4.017375 | Potato root |
| 48R-P4 | Soignies | 50.563978,4.017375 | Potato root |
| 48R-P5 | Soignies | 50.563978,4.017375 | Potato root |
| 48R-P6 | Soignies | 50.563978,4.017375 | Potato root |
| 48R-P7 | Soignies | 50.563978,4.017375 | Potato root |
| 48R-P8 | Soignies | 50.563978,4.017375 | Potato root |
| 48R-P9 | Soignies | 50.563978,4.017375 | Potato root |
| 48L-P1 | Soignies | 50.563978,4.017375 | Potato leaf |
| 48L-P2 | Soignies | 50.563978,4.017375 | Potato leaf |
| 48L-P3 | Soignies | 50.563978,4.017375 | Potato leaf |
| 48L-P4 | Soignies | 50.563978,4.017375 | Potato leaf |
| 48L-P5 | Soignies | 50.563978,4.017375 | Potato leaf |
| 48L-P6 | Soignies | 50.563978,4.017375 | Potato leaf |
| 48L-P7 | Soignies | 50.563978,4.017375 | Potato leaf |
| 48L-P8 | Soignies | 50.563978,4.017375 | Potato leaf |
| 48L-P9 | Soignies | 50.563978,4.017375 | Potato leaf |
| 48T-P1 | Soignies | 50.563978,4.017375 | Potato tuber |
| 48T-P2 | Soignies | 50.563978,4.017375 | Potato tuber |
| 48T-P3 | Soignies | 50.563978,4.017375 | Potato tuber |
| 48T-P4 | Soignies | 50.563978,4.017375 | Potato tuber |
| 48T-P5 | Soignies | 50.563978,4.017375 | Potato tuber |
| 48T-P6 | Soignies | 50.563978,4.017375 | Potato tuber |
| 48T-P7 | Soignies | 50.563978,4.017375 | Potato tuber |
| 48T-P8 | Soignies | 50.563978,4.017375 | Potato tuber |
| 48T-P9 | Soignies | 50.563978,4.017375 | Potato tuber |
| 49R-P3 | Chièvres | 50.550877,3.825468 | Potato root |
| 49R-P4 | Chièvres | 50.550877,3.825468 | Potato root |
| 49R-P3 | Chièvres | 50.550877,3.825468 | Potato root |
| 49R-P4 | Chièvres | 50.550877,3.825468 | Potato root |
| 49R-P5 | Chièvres | 50.550877,3.825468 | Potato root |
| 49R-P6 | Chièvres | 50.550877,3.825468 | Potato root |
| 49R-P7 | Chièvres | 50.550877,3.825468 | Potato root |
| 49R-P8 | Chièvres | 50.550877,3.825468 | Potato root |
| 49R-P9 | Chièvres | 50.550877,3.825468 | Potato root |
| 49L-P3 | Chièvres | 50.550877,3.825468 | Potato leaf |
| 49L-P4 | Chièvres | 50.550877,3.825468 | Potato leaf |
| 49L-P3 | Chièvres | 50.550877,3.825468 | Potato leaf |
| 49L-P4 | Chièvres | 50.550877,3.825468 | Potato leaf |
| 49L-P5 | Chièvres | 50.550877,3.825468 | Potato leaf |
| 49L-P6 | Chièvres | 50.550877,3.825468 | Potato leaf |
| 49L-P7 | Chièvres | 50.550877,3.825468 | Potato leaf |
| 49L-P8 | Chièvres | 50.550877,3.825468 | Potato leaf |
| 49L-P9 | Chièvres | 50.550877,3.825468 | Potato leaf |
| 49T-P3 | Chièvres | 50.550877,3.825468 | Potato tuber |
| 49T-P4 | Chièvres | 50.550877,3.825468 | Potato tuber |
| 49T-P3 | Chièvres | 50.550877,3.825468 | Potato tuber |
| 49T-P4 | Chièvres | 50.550877,3.825468 | Potato tuber |
| 49T-P5 | Chièvres | 50.550877,3.825468 | Potato tuber |
| 49T-P6 | Chièvres | 50.550877,3.825468 | Potato tuber |
| 49T-P7 | Chièvres | 50.550877,3.825468 | Potato tuber |
| 49T-P8 | Chièvres | 50.550877,3.825468 | Potato tuber |
| 49T-P9 | Chièvres | 50.550877,3.825468 | Potato tuber |
| 50R-P3 | Ath | 50.595933,3.710418 | Potato root |
| 50R-P4 | Ath | 50.595933,3.710418 | Potato root |
| 50R-P3 | Ath | 50.595933,3.710418 | Potato root |
| 50R-P4 | Ath | 50.595933,3.710418 | Potato root |
| 50R-P5 | Ath | 50.595933,3.710418 | Potato root |
| 50R-P6 | Ath | 50.595933,3.710418 | Potato root |
| 50R-P7 | Ath | 50.595933,3.710418 | Potato root |
| 50R-P8 | Ath | 50.595933,3.710418 | Potato root |
| 50R-P9 | Ath | 50.595933,3.710418 | Potato root |
| 50L-P3 | Ath | 50.595933,3.710418 | Potato leaf |
| 50L-P4 | Ath | 50.595933,3.710418 | Potato leaf |
| 50L-P3 | Ath | 50.595933,3.710418 | Potato leaf |
| 50L-P4 | Ath | 50.595933,3.710418 | Potato leaf |
| 50L-P5 | Ath | 50.595933,3.710418 | Potato leaf |
| 50L-P6 | Ath | 50.595933,3.710418 | Potato leaf |
| 50L-P7 | Ath | 50.595933,3.710418 | Potato leaf |
| 50L-P8 | Ath | 50.595933,3.710418 | Potato leaf |
| 50L-P9 | Ath | 50.595933,3.710418 | Potato leaf |
| 50T-P3 | Ath | 50.595933,3.710418 | Potato tuber |
| 50T-P4 | Ath | 50.595933,3.710418 | Potato tuber |
| 50T-P3 | Ath | 50.595933,3.710418 | Potato tuber |
| 50T-P4 | Ath | 50.595933,3.710418 | Potato tuber |
| 50T-P5 | Ath | 50.595933,3.710418 | Potato tuber |
| 50T-P6 | Ath | 50.595933,3.710418 | Potato tuber |
| 50T-P7 | Ath | 50.595933,3.710418 | Potato tuber |
| 50T-P8 | Ath | 50.595933,3.710418 | Potato tuber |
| 50T-P9 | Ath | 50.595933,3.710418 | Potato tuber |
| 51R-P1 | Habay | 49.737759,5.616503 | Potato root |
| 51R-P2 | Habay | 49.737759,5.616503 | Potato root |
| 51R-P3 | Habay | 49.737759,5.616503 | Potato root |
| 51R-P4 | Habay | 49.737759,5.616503 | Potato root |
| 51R-P5 | Habay | 49.737759,5.616503 | Potato root |
| 51R-P6 | Habay | 49.737759,5.616503 | Potato root |
| 51R-P7 | Habay | 49.737759,5.616503 | Potato root |
| 51R-P8 | Habay | 49.737759,5.616503 | Potato root |
| 51R-P9 | Habay | 49.737759,5.616503 | Potato root |
| 51L-P1 | Habay | 49.737759,5.616503 | Potato leaf |
| 51L-P2 | Habay | 49.737759,5.616503 | Potato leaf |
| 51L-P3 | Habay | 49.737759,5.616503 | Potato leaf |
| 51L-P4 | Habay | 49.737759,5.616503 | Potato leaf |
| 51L-P5 | Habay | 49.737759,5.616503 | Potato leaf |
| 51L-P6 | Habay | 49.737759,5.616503 | Potato leaf |
| 51L-P7 | Habay | 49.737759,5.616503 | Potato leaf |
| 51L-P8 | Habay | 49.737759,5.616503 | Potato leaf |
| 51L-P9 | Habay | 49.737759,5.616503 | Potato leaf |
| 51T-P1 | Habay | 49.737759,5.616503 | Potato tuber |
| 51T-P2 | Habay | 49.737759,5.616503 | Potato tuber |
| 51T-P3 | Habay | 49.737759,5.616503 | Potato tuber |
| 51T-P4 | Habay | 49.737759,5.616503 | Potato tuber |
| 51T-P5 | Habay | 49.737759,5.616503 | Potato tuber |
| 51T-P6 | Habay | 49.737759,5.616503 | Potato tuber |
| 51T-P7 | Habay | 49.737759,5.616503 | Potato tuber |
| 51T-P8 | Habay | 49.737759,5.616503 | Potato tuber |
| 51T-P9 | Habay | 49.737759,5.616503 | Potato tuber |
| 52R-P1 | Habay | 49.737829,5.615934 | Potato root |
| 52R-P2 | Habay | 49.737829,5.615934 | Potato root |
| 52R-P3 | Habay | 49.737829,5.615934 | Potato root |
| 52R-P4 | Habay | 49.737829,5.615934 | Potato root |
| 52R-P5 | Habay | 49.737829,5.615934 | Potato root |
| 52R-P6 | Habay | 49.737829,5.615934 | Potato root |
| 52R-P7 | Habay | 49.737829,5.615934 | Potato root |
| 52R-P8 | Habay | 49.737829,5.615934 | Potato root |
| 52R-P9 | Habay | 49.737829,5.615934 | Potato root |
| 52L-P1 | Habay | 49.737829,5.615934 | Potato leaf |
| 52L-P2 | Habay | 49.737829,5.615934 | Potato leaf |
| 52L-P3 | Habay | 49.737829,5.615934 | Potato leaf |
| 52L-P4 | Habay | 49.737829,5.615934 | Potato leaf |
| 52L-P5 | Habay | 49.737829,5.615934 | Potato leaf |
| 52L-P6 | Habay | 49.737829,5.615934 | Potato leaf |
| 52L-P7 | Habay | 49.737829,5.615934 | Potato leaf |
| 52L-P8 | Habay | 49.737829,5.615934 | Potato leaf |
| 52L-P9 | Habay | 49.737829,5.615934 | Potato leaf |
| 52T-P1 | Habay | 49.737829,5.615934 | Potato tuber |
| 52T-P2 | Habay | 49.737829,5.615934 | Potato tuber |
| 52T-P3 | Habay | 49.737829,5.615934 | Potato tuber |
| 52T-P4 | Habay | 49.737829,5.615934 | Potato tuber |
| 52T-P5 | Habay | 49.737829,5.615934 | Potato tuber |
| 52T-P6 | Habay | 49.737829,5.615934 | Potato tuber |
| 52T-P7 | Habay | 49.737829,5.615934 | Potato tuber |
| 52T-P8 | Habay | 49.737829,5.615934 | Potato tuber |
| 52T-P9 | Habay | 49.737829,5.615934 | Potato tuber |
| 53R-P1 | Habay | 49.724403,5.601751 | Potato root |
| 53R-P2 | Habay | 49.724403,5.601751 | Potato root |
| 53R-P3 | Habay | 49.724403,5.601751 | Potato root |
| 53R-P4 | Habay | 49.724403,5.601751 | Potato root |
| 53R-P5 | Habay | 49.724403,5.601751 | Potato root |
| 53R-P6 | Habay | 49.724403,5.601751 | Potato root |
| 53R-P7 | Habay | 49.724403,5.601751 | Potato root |
| 53R-P8 | Habay | 49.724403,5.601751 | Potato root |
| 53R-P9 | Habay | 49.724403,5.601751 | Potato root |
| 53L-P1 | Habay | 49.724403,5.601751 | Potato leaf |
| 53L-P2 | Habay | 49.724403,5.601751 | Potato leaf |
| 53L-P3 | Habay | 49.724403,5.601751 | Potato leaf |
| 53L-P4 | Habay | 49.724403,5.601751 | Potato leaf |
| 53L-P5 | Habay | 49.724403,5.601751 | Potato leaf |
| 53L-P6 | Habay | 49.724403,5.601751 | Potato leaf |
| 53L-P7 | Habay | 49.724403,5.601751 | Potato leaf |
| 53L-P8 | Habay | 49.724403,5.601751 | Potato leaf |
| 53L-P9 | Habay | 49.724403,5.601751 | Potato leaf |
| 53T-P1 | Habay | 49.724403,5.601751 | Potato tuber |
| 53T-P2 | Habay | 49.724403,5.601751 | Potato tuber |
| 53T-P3 | Habay | 49.724403,5.601751 | Potato tuber |
| 53T-P4 | Habay | 49.724403,5.601751 | Potato tuber |
| 53T-P5 | Habay | 49.724403,5.601751 | Potato tuber |
| 53T-P6 | Habay | 49.724403,5.601751 | Potato tuber |
| 53T-P7 | Habay | 49.724403,5.601751 | Potato tuber |
| 53T-P8 | Habay | 49.724403,5.601751 | Potato tuber |
| 53T-P9 | Habay | 49.724403,5.601751 | Potato tuber |
| P1A-P1 | Beauraing | 50.107616,4.974082 | Vegetable garden |
| P1A-P2 | Beauraing | 50.107616,4.974082 | Vegetable garden |
| P1A-P3 | Beauraing | 50.107616,4.974082 | Vegetable garden |
| P1A-P4 | Beauraing | 50.107616,4.974082 | Vegetable garden |
| P1A-P5 | Beauraing | 50.107616,4.974082 | Vegetable garden |
| P1A-P6 | Beauraing | 50.107616,4.974082 | Vegetable garden |
| P1A-P7 | Beauraing | 50.107616,4.974082 | Vegetable garden |
| P1A-P8 | Beauraing | 50.107616,4.974082 | Vegetable garden |
| P1A-P9 | Beauraing | 50.107616,4.974082 | Vegetable garden |
| P2A-P1 | Bertrix | 49.85128,5.25257 | Vegetable garden |
| P2A-P2 | Bertrix | 49.85128,5.25257 | Vegetable garden |
| P2A-P3 | Bertrix | 49.85128,5.25257 | Vegetable garden |
| P2A-P4 | Bertrix | 49.85128,5.25257 | Vegetable garden |
| P2A-P5 | Bertrix | 49.85128,5.25257 | Vegetable garden |
| P2A-P6 | Bertrix | 49.85128,5.25257 | Vegetable garden |
| P2A-P7 | Bertrix | 49.85128,5.25257 | Vegetable garden |
| P2A-P8 | Bertrix | 49.85128,5.25257 | Vegetable garden |
| P2A-P9 | Bertrix | 49.85128,5.25257 | Vegetable garden |
| P3A-P1 | Bertrix | 49.85128,5.25257 | Vegetable garden |
| P3A-P2 | Bertrix | 49.85128,5.25257 | Vegetable garden |
| P3A-P3 | Bertrix | 49.85128,5.25257 | Vegetable garden |
| P3A-P4 | Bertrix | 49.85128,5.25257 | Vegetable garden |
| P3A-P5 | Bertrix | 49.85128,5.25257 | Vegetable garden |
| P3A-P6 | Bertrix | 49.85128,5.25257 | Vegetable garden |
| P3A-P7 | Bertrix | 49.85128,5.25257 | Vegetable garden |
| P3A-P8 | Bertrix | 49.85128,5.25257 | Vegetable garden |
| P3A-P9 | Bertrix | 49.85128,5.25257 | Vegetable garden |
| P4A-P1 | Bertrix | 49.84403,5.265884 | Vegetable garden |
| P4A-P2 | Bertrix | 49.84403,5.265884 | Vegetable garden |
| P4A-P3 | Bertrix | 49.84403,5.265884 | Vegetable garden |
| P4A-P4 | Bertrix | 49.84403,5.265884 | Vegetable garden |
| P4A-P5 | Bertrix | 49.84403,5.265884 | Vegetable garden |
| P4A-P6 | Bertrix | 49.84403,5.265884 | Vegetable garden |
| P4A-P7 | Bertrix | 49.84403,5.265884 | Vegetable garden |
| P4A-P8 | Bertrix | 49.84403,5.265884 | Vegetable garden |
| P4A-P9 | Bertrix | 49.84403,5.265884 | Vegetable garden |
| P5A-P1 | Bertrix | 49.782615,5.213131 | Vegetable garden |
| P5A-P2 | Bertrix | 49.782615,5.213131 | Vegetable garden |
| P5A-P3 | Bertrix | 49.782615,5.213131 | Vegetable garden |
| P5A-P4 | Bertrix | 49.782615,5.213131 | Vegetable garden |
| P5A-P5 | Bertrix | 49.782615,5.213131 | Vegetable garden |
| P5A-P6 | Bertrix | 49.782615,5.213131 | Vegetable garden |
| P5A-P7 | Bertrix | 49.782615,5.213131 | Vegetable garden |
| P5A-P8 | Bertrix | 49.782615,5.213131 | Vegetable garden |
| P5A-P9 | Bertrix | 49.782615,5.213131 | Vegetable garden |
| P1B-P1 | Beauraing | 50.107616,4.974082 | Vegetable garden |
| P1B-P2 | Beauraing | 50.107616,4.974082 | Vegetable garden |
| P1B-P3 | Beauraing | 50.107616,4.974082 | Vegetable garden |
| P1B-P4 | Beauraing | 50.107616,4.974082 | Vegetable garden |
| P1B-P5 | Beauraing | 50.107616,4.974082 | Vegetable garden |
| P1B-P6 | Beauraing | 50.107616,4.974082 | Vegetable garden |
| P1B-P7 | Beauraing | 50.107616,4.974082 | Vegetable garden |
| P1B-P8 | Beauraing | 50.107616,4.974082 | Vegetable garden |
| P1B-P9 | Beauraing | 50.107616,4.974082 | Vegetable garden |
| P2B-P1 | Bertrix | 49.85128,5.25257 | Vegetable garden |
| P2B-P2 | Bertrix | 49.85128,5.25257 | Vegetable garden |
| P2B-P3 | Bertrix | 49.85128,5.25257 | Vegetable garden |
| P2B-P4 | Bertrix | 49.85128,5.25257 | Vegetable garden |
| P2B-P5 | Bertrix | 49.85128,5.25257 | Vegetable garden |
| P2B-P6 | Bertrix | 49.85128,5.25257 | Vegetable garden |
| P2B-P7 | Bertrix | 49.85128,5.25257 | Vegetable garden |
| P2B-P8 | Bertrix | 49.85128,5.25257 | Vegetable garden |
| P2B-P9 | Bertrix | 49.85128,5.25257 | Vegetable garden |
| P3B-P1 | Bertrix | 49.85128,5.25257 | Vegetable garden |
| P3B-P2 | Bertrix | 49.85128,5.25257 | Vegetable garden |
| P3B-P3 | Bertrix | 49.85128,5.25257 | Vegetable garden |
| P3B-P4 | Bertrix | 49.85128,5.25257 | Vegetable garden |
| P3B-P5 | Bertrix | 49.85128,5.25257 | Vegetable garden |
| P3B-P6 | Bertrix | 49.85128,5.25257 | Vegetable garden |
| P3B-P7 | Bertrix | 49.85128,5.25257 | Vegetable garden |
| P3B-P8 | Bertrix | 49.85128,5.25257 | Vegetable garden |
| P3B-P9 | Bertrix | 49.85128,5.25257 | Vegetable garden |
| P4B-P1 | Bertrix | 49.84403,5.265884 | Vegetable garden |
| P4B-P2 | Bertrix | 49.84403,5.265884 | Vegetable garden |
| P4B-P3 | Bertrix | 49.84403,5.265884 | Vegetable garden |
| P4B-P4 | Bertrix | 49.84403,5.265884 | Vegetable garden |
| P4B-P5 | Bertrix | 49.84403,5.265884 | Vegetable garden |
| P4B-P6 | Bertrix | 49.84403,5.265884 | Vegetable garden |
| P4B-P7 | Bertrix | 49.84403,5.265884 | Vegetable garden |
| P4B-P8 | Bertrix | 49.84403,5.265884 | Vegetable garden |
| P4B-P9 | Bertrix | 49.84403,5.265884 | Vegetable garden |
| P5B-P1 | Bertrix | 49.782615,5.213131 | Vegetable garden |
| P5B-P2 | Bertrix | 49.782615,5.213131 | Vegetable garden |
| P5B-P3 | Bertrix | 49.782615,5.213131 | Vegetable garden |
| P5B-P4 | Bertrix | 49.782615,5.213131 | Vegetable garden |
| P5B-P5 | Bertrix | 49.782615,5.213131 | Vegetable garden |
| P5B-P6 | Bertrix | 49.782615,5.213131 | Vegetable garden |
| P5B-P7 | Bertrix | 49.782615,5.213131 | Vegetable garden |
| P5B-P8 | Bertrix | 49.782615,5.213131 | Vegetable garden |
| P5B-P9 | Bertrix | 49.782615,5.213131 | Vegetable garden |

**Table S2.** Characteristics of primer pairs used in this study

| **Family** | **Antagonistic molecule** | **Primer pair** | **Sequence (5’ to 3’) ^a^** | **Expected**  **amplicon size (bp)** | **Annealing temp. (°C)** | **Positive control ^b^** | **Reference** |
| --- | --- | --- | --- | --- | --- | --- | --- |
| ***Bacillus* spp.** | | | | | | | |
| **Enzymes** | **AHL-lactonases** | Lac F  Lac R | ATCGGATCCATGACAGTAAAGAAGCTTTATTTCG  GTCGAATTCCTCAACAAGATACTCCTAATGATGT | ~ 1000 | 50 | *Btk* HD1 | (Dong et al., 2000) |
|  | **Exochitinase** | Chi36F Chi36R | GATGTTAAACAGGTTCAA  TTATTTTTGCAAGGAAAG | 1083 | 50 | *Btk* HD1 | (Arora et al., 2003) |
|  |  | Chi1  Chi3 | ATGGCTATGAGGTCTCAAAAATTCA  CTAACAGGTGACTATCTTCTTATAT | ~ 2300 | 48 | *Btk* HD1 | (Driss et al., 2005) |
|  | **Glucanase** | Glucanase F Glucanase R | TTCTTGTCACTGGATTGT  CGCACCAGTCATAATGAGCG | 684 | 56 | *Bam* ATCC 23350 | (Raddadi et al., 2009) |
|  | **Malonyl-CoA ACP transacylase** | MalacptransacF MalacptransacR | ATTGTATTTATGTTTCCTGGCGTA  TTTGTTCTCTCTCTTTAATCTGTT | 1172 | 60 | Btk HD1 | (Raddadi et al., 2009) |
| **Lipopeptides** | **Bacillomycin D** | BAMC-F1 BAM-R1 | AGTAAATGAACGCGCCA CCCTCTCCTGCCACATAGAG | 957 | 57 | - | (Chung et al., 2008) |
|  | **Fengycin** | FENDF FENDR | GGCCCGTTCTCTAAATCCAT GTCATGCTGACGAGAGCAAA | 269 | 58 | *Bam* ATCC 23350 | (Mora et al., 2011) |
|  | **Iturin**  **(A, B, C, D)** | ITUP1-F ITUP2-R | AGCTTAGGGAACAATTGTCATCGGGGCTTC  TCAGATAGGCCGCC ATATCGGAATGATTCG | ~ 2000 | 60 | *Bam* ATCC 23350 | (Tsuge et al., 2005) |
|  |  | *lpa-*14f *lpa-*14r | ATGAAAATTTACGGAGTATA TTATAACAGCTCTTCATACG | 675 | 50 | *Bam* ATCC 23350 | (Hsieh et al., 2008) |
|  | **Plipastatin** | Plipastatin synF1 Plipastatin syn R1 | CTGTTCACGGTTCCATGTTG  GCCGCAGCTTAGCTATATG | 2153 | 63 | *B. subtilis* ATCC 6051 | This work |
|  |  | Plipastatin syn F2 Plipastatin syn R2 | CCGGACGTCGTTTATCTGTT  TGTTTCACAAGCGCATCTTC | 2054 | 63 | *B. subtilis* ATCC 6051 | This work |
|  | **Surfactin** | SRFA1-F SRFA1-R | AGAGCACATTGAGCGTTACAAA CAGCATCTCGTTCAACTTTCAC | 626 | 57 | *B. subtilis* ATCC 6051 | (Chung et al., 2008) |
|  |  | SRFA2-F  SRFA2-R | TCGGGACAGGAAGACATCAT CCACTCAAACGGATAATCCTGA | 201 | 58 | *B. subtilis* ATCC 6051 | (Mora et al., 2011) |
|  |  | sfp-F sfp-R | ATGAAGATTTACGGAATTTA TTATAAAAGCTCTTCGTACG | 675 | 46 | *B. subtilis* ATCC 6051 | (Hsieh et al., 2004) |

**Table S2. (Continued)**

| **Family** | **Antagonistic molecule** | **Primer pair** | **Sequence (5’ to 3’) ^a^** | **Expected**  **amplicon size (bp)** | **Annealing temp. (°C)** | **Positive control ^b^** | **Reference** |
| --- | --- | --- | --- | --- | --- | --- | --- |
| ***Bacillus* spp.** | | | | | | | |
| **Peptides** | **Bacilysin** | BACab-F1  BACab-R1 | CTTCTCCAAGGGGTGAACAG TGTAGGTTTCACCGGCTTTC | 815 | 52 | *Bam* ATCC 23350 | (Hsieh et al., 2008) |
|  |  | BACD-F1 BACD-R1 | AAAAACAGTATTGGTYATCGCTGA CCATGATGCCTTCKATRCTGAT | 749 | 55 | *Bam* ATCC 23350 | (Hsieh et al., 2008) |
|  | **Zwittermicin A** | A0677 A0678 | ATGTGCACTTGTATGGGCAG TAAAGCTCGTCCCTCTTCAG | ~ 1000 | 63 | *B. cereus* UW85 | (Raffel et al., 1996) |
| **Polyketides** | **Difficidin** | dfnAF1  dfnAR1 | GGTGCGGCATGAAGATTTGAGATCACCG  GGAGAGCACTTCAATTCCGACGTTGACC | ~1900 | 60 | *Bam* GA1 | (Arguelles-Arias et al., 2009b) |
|  |  | dfnMF1 dfnMR1 | CGGAGTGAAACCGTGCCGGGATAAAGA  GACCATTCAGAGCGGAAAGCTCC | ~1300 | 60 | *Bam* GA1 | (Arguelles-Arias et al., 2009b) |
|  | **Macrolactin** | mlnAF1 mlnAR1 | CGGCTGCGGGGGAAAAGATCCG  CAGCATCAGGGCGTGTATGACCTTC | ~1300 | 60 | *Bam* GA1 | (Arguelles-Arias et al., 2009b) |
|  |  | mlnIF1 mlnIR1 | GGAAGAAAAACAGTCGAGGCGATGCTG  GAGAAGCTCCGCCGTCACCAGTG | ~1300 | 60 | *Bam* GA1 | (Arguelles-Arias et al., 2009b) |
| **Virulence factors** | **Cereulide** | CesF1  CesR2 | GGTGACACATTATCATATAAGGTG  GTAAGCGAACCTGTCTGTAACAACA | 1271 | 60 | *B. cereus* 5975c | (Ehling-Schulz et al., 2005) |
|  |  | CER1  EMT1 | ATCATAAAGGTGCGAACAAGA  AAGATCAACCGAATGCAACTG | 188 | 55 | *B. cereus* 5975c | (Horwood et al., 2004) |
|  |  | EM1F  EM1R | GACAAGAGAAATTTCTACGAGCAAGTACAAT  GCAGCCTTCCAATTACTCCTTCTGCCACAGT | 635 | 60 | *B. cereus* 5975c | (Ehling-Schulz et al., 2004) |
|  | **Cereolysin O** | Hem41  Hem42 | ACGTCACCAGTMGATATWTC  TCTCCACCATTCCCAWGCAAG | 1146 | 60 | *B. cereus* 5975c | (Michelet, 2005) |
|  | **Non-Hemolytic enterotoxin (NheA)** | NheAF  NheAR | GTTAGGATCACAATCACCGC  ACGAATGTAATTTGAGTCGC | 755 | 56 | *B. cereus* 5975c | (Guinebretière et al., 2002) |

**Table S2. (Continued)**

| **Family** | **Antagonistic molecule** | **Primer pair** | **Sequence (5’ to 3’) ^a^** | **Expected**  **amplicon size (bp)** | **Annealing temp. (°C)** | **Positive control ^b^** | **Reference** |
| --- | --- | --- | --- | --- | --- | --- | --- |
| ***Bacillus* spp.** | | | | | | | |
| **Virulence factors** | **Hemolysin BL (HBLD and HBLA)** | HD2F  HA4R | GTAAATTAIGATGAICAATTTC  AGAATAGGCATTCATAGATT | 1091 | 56 | *B. cereus* ATCC 14579 | (Ehling-Schulz et al., 2006) |
|  | **Cytotoxin K1** | CK1F  CK1R | CAATTCCAGGGGCAAGTGTC  CCTCGTGCATCTGTTTCATGAG | 426 | 61 | *B. cytotoxicus LMG26718^T^* | (Guinebretière et al., 2006) |
|  | **Cytotoxin K2** | CK2F  CK2R | CAATCCCTGGCGCTAGTGCA  GTGIAGCCTGGACGAAGTTGG | 585 | 61 | *B. cereus* ATCC 14579 | (Guinebretière et al., 2006) |
| ***Pseudomonas* spp.** | | | | | | | |
| **Amino acid derived** | **Phenazin** | PCA2a  PCA3b | TTGCCAAGCCTCGCTCCAAC  CCGCGTTGTTCCTCGTTCAT | 1150 | 64 | *P. fluorescens* 2-79 * | (Raaijmakers et al., 1997) |
|  |  | PHZ1  PHZ2 | GGCGACATGGTCAACGG  CGGCTGGCGGCGTATAT | 1408 | 62 | *P. aureofaciens* 30-84 * | (Delaney et al., 2001) |
|  |  | phzIF  phzIR | CATCAGCTTAGCAATCCC CGGAGAAACTTTTCCCTC | 392 | 54 | *P. aeruginosa* LMG1242 | (Finnan et al., 2004) |
|  |  | phzSF  phzSR | TCGCCATGACCGATACGCTC  ACAACCTGAGCCAGCCTTCC | 1752 | 62 | *P. aeruginosa* LMG1242 | (Finnan et al., 2004) |
|  | **Pyrrolnitrin** | PrnCF  PrnCR | CCACAAGCCCGGCCAGGAGC  GAGAAGAGCGGGTCGATGAAGCC | 719 | 68 | *P. fluorescens* Pf-5 * | (Mavrodi et al., 2001) |

**Table S2. (Continued)**

| **Family** | **Antagonistic molecule** | **Primer pair** | **Sequence (5’ to 3’) ^a^** | **Expected**  **amplicon size (bp)** | **Annealing temp. (°C)** | **Positive control ^b^** | **Reference** |
| --- | --- | --- | --- | --- | --- | --- | --- |
| ***Pseudomonas* spp.** | | | | | | | |
| **Lipopeptides** | **Orfamide A** | OfaF  OfaR | GGTACTGGGGCGTACCAGCG  GCTCCCAGGTACTGCGGTCG | 980 | 67 | *P. protegens* PGNR1 * | This work |
|  |  | OfaBF  OfaBR | TTCAACTGGCGGGACGTGTGG  CGGCCGGGGGATCACATAGG | 825 | 68 | *P. protegens* PGNR1 * | This work |
|  | **Putisolvin** | PsoaF  PsoaR | AACGCCTGGCCTACCTGCTG  CCCCTTGCCGCCCACATACA | 755 | 65 | *P. putida* PCL1445 * | This work |
|  |  | PsobF  PsobR | ACCCTGAGTACCTGCGCCAA  GGCGGGAGGTCAGGGTCACT | 966 | 65 | *P. putida* PCL1445 * | This work |
|  | **Syringopeptin** | SypAF  SypAR | TGGACCCGGCAGTGATTGCTC  GGCACTACAACTTCCGTTCCGC | 958 | 65 | *P. syringae* pv*. syringae* B301D * | This work |
|  |  | SypBF  SypBR | GTGAACCAGGCAACGGGGAA  CTCTCCGCCCAGCATACGGC | 980 | 67 | *P. syringae* pv*. syringae* B301D * | This work |
|  | **Syringotoxin** | SyrBF  SyrBR | CTTTCCGTGGTCTTGATGAGG  TCGATTTTGCCGTGATGAGTC | 752 | 61 | *P. syringae* pv*. syringae* B301D * | (Sorensen et al., 1998) |
|  | **Viscosin** | ViscAF  ViscAR | GGGCGGTGCGCGTGAAATAC  CGGCCAGCTTCCCCCTTACC | 774 | 68 | *P. fluorescens* BBc6R8 * | This work |
|  |  | ViscBF  ViscBR | GCGGCGTACTGCCGGATACA  CGCTTCGGTGGGGCCATACA | 800 | 68 | *P. fluorescens* BBc6R8 * | This work |
|  |  | ViscCF  ViscCR | ACGTATGGGGGCCGTTGCTC  ATCGGCTGGCGCCTCGAAAT | 923 | 68 | *P. fluorescens* BBc6R8 * | This work |

**Table S2. (Continued)**

| **Family** | **Antagonistic molecule** | **Primer pair** | **Sequence (5’ to 3’) ^a^** | **Expected**  **amplicon size (bp)** | **Annealing temp. (°C)** | **Positive control ^b^** | **Reference** |
| --- | --- | --- | --- | --- | --- | --- | --- |
| ***Pseudomonas* spp.** | | | | | | | |
| **Polyketides** | **2,4-diacetyl-phloroglunicol** | Phl2a  Phl2b | GAGGACGTCGAAGACCACCA  ACCGCAGCATCGTGTATGAG | 745 | 62 | *P. fluorescens*  Q2-87 * | (Raaijmakers et al., 1997) |
|  | **Pyoluteorin** | PltBf  Pltbr | CGGAGCATGGACCCCCAGC  GTGCCCGATATTGGTCTTGACC | 773 | 64 | *P. fluorescens* Pf-5 * | (Mavrodi et al., 2001) |
|  |  | PLTC1  PLTC2 | AACAGATCGCCCCGGTACAGAACG  AGGCCCGGACACTCAAGAAACTCG | 436 | 68 | *P. fluorescens* Pf-5 * | (de Souza and Raaijmakers, 2003) |
| **Virulence factors** | **Elastase** | lasAF  lasAR | GCAGCACAAAAGATCCC  GAAATGCAGGTGCGGTC | 1075 | 56 | *P. aeruginosa* PAO1 | (Finnan et al., 2004) |
|  |  | lasB f  lasB r | GGAATGAACGAAGCGTTCTC  GGTCCAGTAGTAGCGGTTGG | 300 | 587 | *P. aeruginosa* PAO1 | (Holban et al., 2013a) |
|  | **Exotoxin S** | ExoS1  ExoS2 | ATCGCTTCAGCAGAGTCCGTC  CAGGCCAGATCAAGGCCGCGC | 1352 | 63 | *P. aeruginosa* PAO1 | (Holban et al., 2013a) |
|  | **Exotoxin T** | exoTF  exoTR | CAATCATCTCAGCAGAACCC  TGTCGTAGAGGATCTCCTG | 1159 | 55 | *P. aeruginosa* PAO1 | (Finnan et al., 2004) |
|  | **Exotoxin U** | exoUF  exoUR | GATTCCATCACAGGCTCG  CTAGCAATGGCACTAATCG | 3308 | 53 | *P. aeruginosa* PAO1 | (Finnan et al., 2004) |
|  | **Exotoxin Y** | exoYF  exoYR | TATCGACGGTCATCGTCAGGT  TTGATGCACTCGACCAGCAAG | 1035 | 62 | *P. aeruginosa* PAO1 | (Finnan et al., 2004) |
|  | **Pili** | pilAF  pilAR | ACAGCATCCAACTGAGCG  TTGACTTCCTCCAGGCTG | 1675 | 56 | *P. aeruginosa* PAO1 | (Finnan et al., 2004) |
|  |  | pilBF  pilBR | TCGAACTGATGATCGTGG  CTTTCGGAGTGAACATCG | 408 | 54 | *P. aeruginosa* PAO1 | (Finnan et al., 2004) |
|  | **Protease Alkaline** | aprF  aprR | TGTCCAGCAATTCTCTTGC  CGTTTTCCACGGTGACC | 1017 | 57 | *P. aeruginosa* LMG1242 | (Finnan et al., 2004) |
|  | **Protease IV** | Protease IV-1  Protease IV-2 | TATTTCGCCGACTCCCTGTA  GAATAGACGCCGCTGAAATC | 752 | 59 | *P. aeruginosa* LMG1242 | (Holban et al., 2013a) |
|  | **Pyoverdin** | pvdAF  pvdAR | GACTCAGGCAACTGCAAC  TTCAGGTGCTGGTACAGG | 1281 | 60 | *P. aeruginosa* PAO1 | (Finnan et al., 2004) |

**References**

Arguelles-Arias, A., Ongena, M., Halimi, B., Lara, Y., Brans, A., Joris, B., Fickers, P., 2009. *Bacillus amyloliquefaciens* GA1 as a source of potent antibiotics and other secondary metabolites for biocontrol of plant pathogens. Microb Cell Fact 8**,** 63.

Arora, N., Ahmad, T., Rajagopal, R., Bhatnagar, R.K., 2003. A constitutively expressed 36 kDa exochitinase from *Bacillus thuringiensis* HD-1. Biochem Biophys Res Commun 307**,** 620-625.

Chung, S., Kong, H., Buyer, J.S., Lakshman, D.K., Lydon, J., Kim, S.-D., Roberts, D.P., 2008. Isolation and partial characterization of *Bacillus subtilis* ME488 for suppression of soilborne pathogens of cucumber and pepper. Appl Microbiol Biotechnol 80**,** 115-123.

de Souza, J.T., Raaijmakers, J.M., 2003. Polymorphisms within the *prnD* and *pltC* genes from pyrrolnitrin and pyoluteorin-producing *Pseudomonas* and *Burkholderia* spp. FEMS Microbiol Ecol 43**,** 21-34.

Delaney, S.M., Mavrodi, D.V., Bonsall, R.F., Thomashow, L.S., 2001. *phzO*, a gene for biosynthesis of 2-hydroxylated phenazine compounds in *Pseudomonas aureofaciens* 30-84. J Bacteriol 183**,** 318-327.

Dong, Y.-H., Xu, J.-L., Li, X.-Z., Zhang, L.-H., 2000. AiiA, an enzyme that inactivates the acylhomoserine lactone quorum-sensing signal and attenuates the virulence of *Erwinia carotovora*. Proc Natl Acad Sci 97**,** 3526-3531.

Driss, F., Kallassy-Awad, M., Zouari, N., Jaoua, S., 2005. Molecular characterization of a novel chitinase from *Bacillus thuringiensis* subsp. *kurstaki*. J Appl Microbiol 99**,** 945-953.

Ehling-Schulz, M., Fricker, M., Scherer, S., 2004. Identification of emetic toxin producing *Bacillus cereus* strains by a novel molecular assay. FEMS Microbiol Lett 232**,** 189-195.

Ehling-Schulz, M., Guinebretiere, M.H., Monthan, A., Berge, O., Fricker, M., Svensson, B., 2006. Toxin gene profiling of enterotoxic and emetic *Bacillus cereus*. FEMS Microbiol Lett 260**,** 232-240.

Ehling-Schulz, M., Vukov, N., Schulz, A., Shaheen, R., Andersson, M., Martlbauer, E., Scherer, S., 2005. Identification and partial characterization of the nonribosomal peptide synthetase gene responsible for cereulide production in emetic *Bacillus cereus*. Appl Environ Microbiol 71**,** 105-113.

Finnan, S., Morrissey, J.P., O'Gara, F., Boyd, E.F., 2004. Genome diversity of *Pseudomonas aeruginosa* isolates from cystic fibrosis patients and the hospital environment. J Clin Microbiol 42**,** 5783-5792.

Guinebretière, M.-H., Broussolle, V., Nguyen-The, C., 2002. Enterotoxigenic profiles of food-poisoning and food-borne *Bacillus cereus* strains. J Clin Microbiol 40**,** 3053-3056.

Guinebretiere, M.-H., Fagerlund, A., Granum, P.E., Nguyen-The, C., 2006. Rapid discrimination of *cytK-1* and *cytK-2* genes in *Bacillus cereus* strains by a novel duplex PCR system. FEMS Microbiol Lett 259**,** 74-80.

Holban, A.-M., Chifiriuc, M.C., Cotar, A.I., Bleotu, C., Grumezescu, A.M., Banu, O., Lazar, V., 2013. Virulence markers in *Pseudomonas aeruginosa* isolates from hospital-acquired infections occurred in patients with underlying cardiovascular disease. Rom Biotechnol Letters 18**,** 8843-8854.

Horwood, P.F., Burgess, G.W., Oakey, H.J., 2004. Evidence for non-ribosomal peptide synthetase production of cereulide (the emetic toxin) in *Bacillus cereus*. FEMS Microbiol Lett 236**,** 319-324.

Hsieh, F.-C., Li, M.-C., Lin, T.-C., Kao, S.-S., 2004. Rapid detection and characterization of surfactin-producing *Bacillus subtilis* and closely related species based on PCR. Curr Microbiol 49**,** 186-191.

Hsieh, F.C., Lin, T.C., Meng, M., Kao, S.S., 2008. Comparing methods for identifying *Bacillus* strains capable of producing the antifungal lipopeptide iturin A. Curr Microbiol 56**,** 1-5.

Mavrodi, O.V., McSpadden Gardener, B.B., Mavrodi, D.V., Bonsall, R.F., Weller, D.M., Thomashow, L.S., 2001. Genetic diversity of *phlD* from 2,4-diacetylphloroglucinol-producing fluorescent *Pseudomonas* spp. Phytopathology 91**,** 35-43.

Michelet, N., 2005. Genetic and genomic characterization of Cereolysin O, a hemolysin of *Bacillus cereus sensu lato*. Ph.D. thesis. Université catholique de Louvain. 153 pp.

Mora, I., Cabrefiga, J., Montesinos, E., 2011. Antimicrobial peptide genes in *Bacillus* strains from plant environments. Int Microbiol 14**,** 213-223.

Raaijmakers, J.M., Weller, D.M., Thomashow, L.S., 1997. Frequency of antibiotic-producing *Pseudomonas* spp. in natural environments. Appl Environ Microbiol 63**,** 881-887.

Raddadi, N., Belaouis, A., Tamagnini, I., Hansen, B.M., Hendriksen, N.B., Boudabous, A., Cherif, A., Daffonchio, D., 2009. Characterization of polyvalent and safe *Bacillus thuringiensis* strains with potential use for biocontrol. J Basic Microbiol 49**,** 293-303.

Raffel, S.J., Stabb, E.V., Milner, J.L., Handelsman, J., 1996. Genotypic and phenotypic analysis of zwittermicin A-producing strains of *Bacillus cereus*. Microbiology 142**,** 3425-3436.

Sorensen, K.N., Kim, K.-H., Takemoto, J.Y., 1998. PCR detection of cyclic lipodepsinonapeptide-producing *Pseudomonas syringae* pv. *syringae* and similarity of strains. Appl Environ Microbiol 64**,** 226-230.

Tsuge, K., Inoue, S., Ano, T., Itaya, M., Shoda, M., 2005. Horizontal transfer of iturin A operon, *itu*, to *Bacillus subtilis* 168 and conversion into an iturin A producer. Antimicrob Agents Chemother 49**,** 4641-4648.
