# Supplementary figures and images for: Versatile Antagonistic Activities of Soil-Borne Bacillus spp. and Pseudomonas spp. against Phytophthora infestans and Other Potato Pathogens
Source: Front Microbiol. 2018 Feb 13;9:143. doi: 10.3389/fmicb.2018.00143 (PMC5816801; doi:10.3389/fmicb.2018.00143)

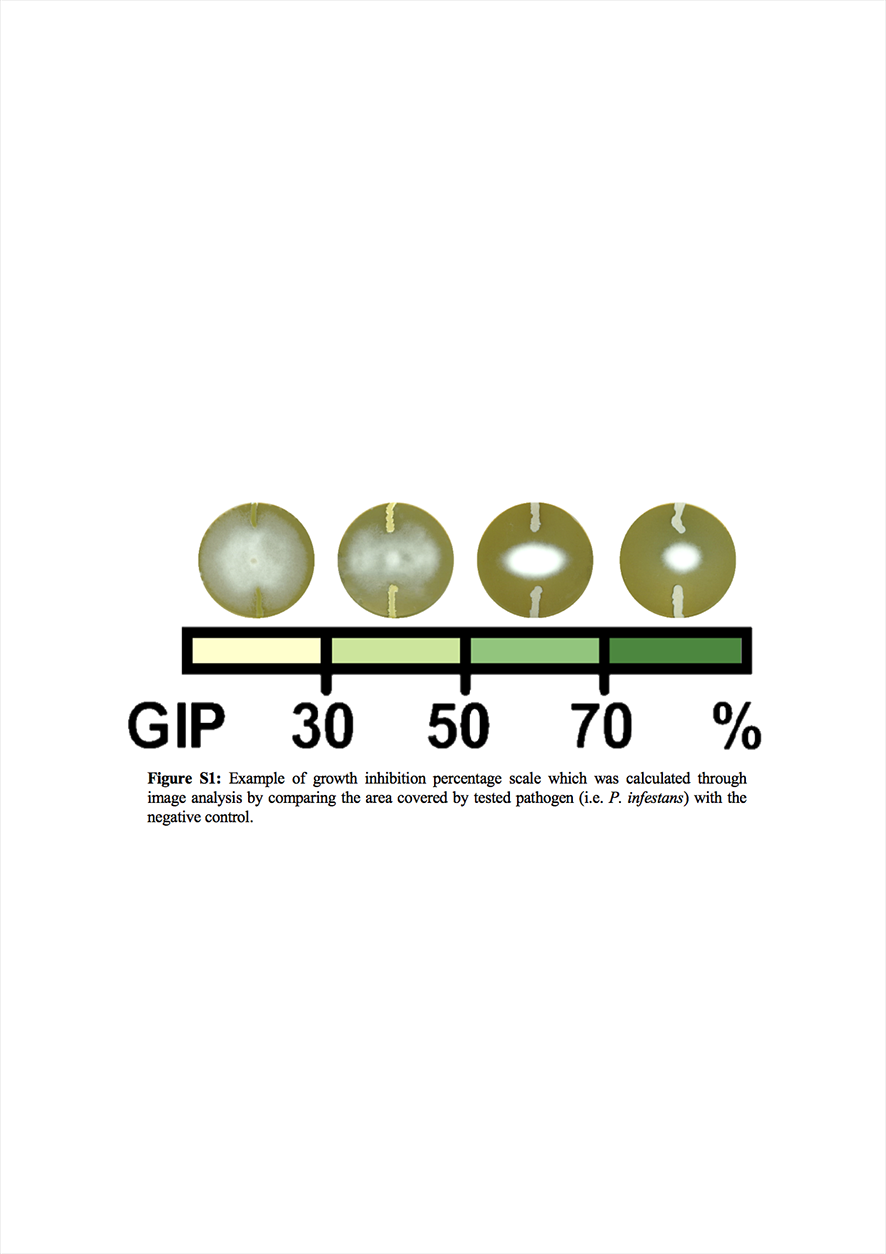

Supplement: Supplementary file 2 [file Image1.TIFF]

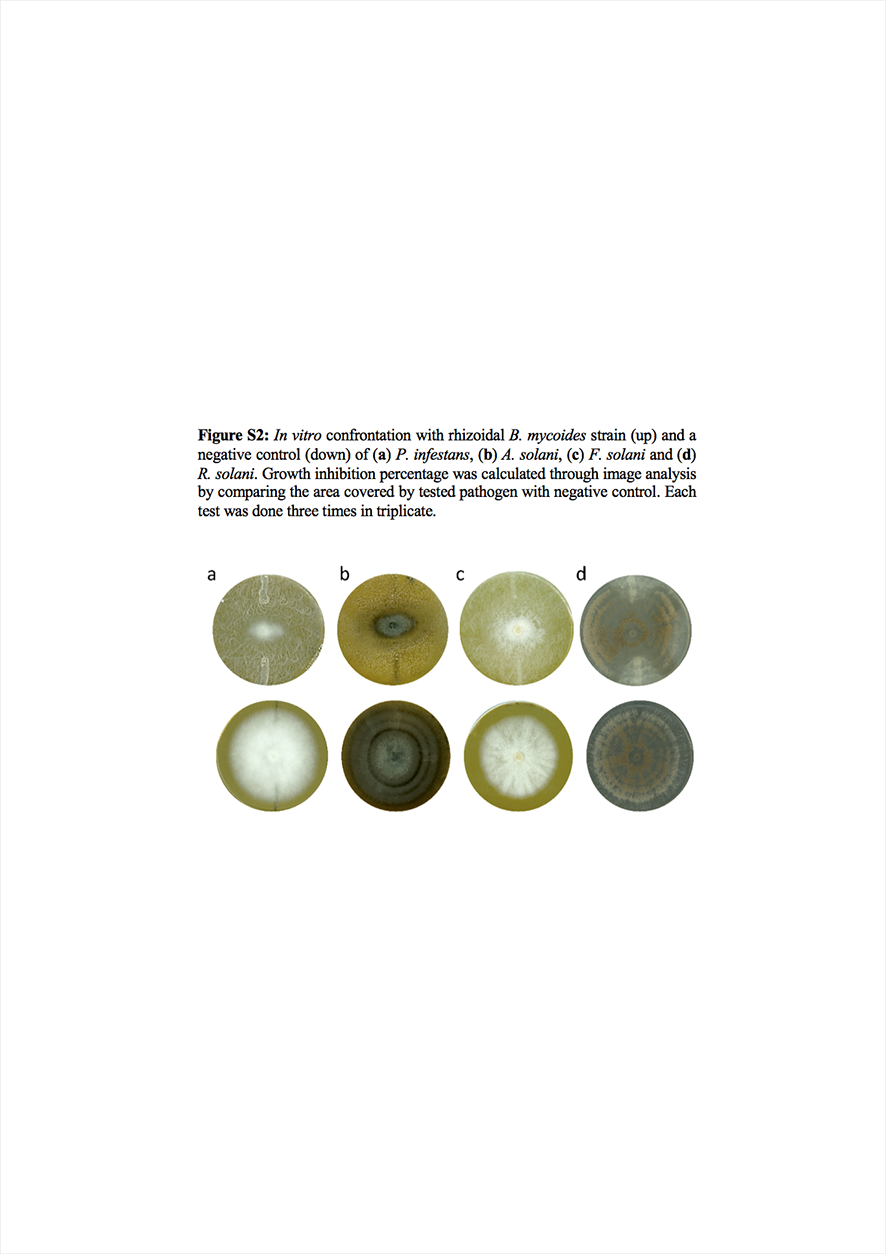

Supplement: Supplementary file 3 [file Image2.TIFF]

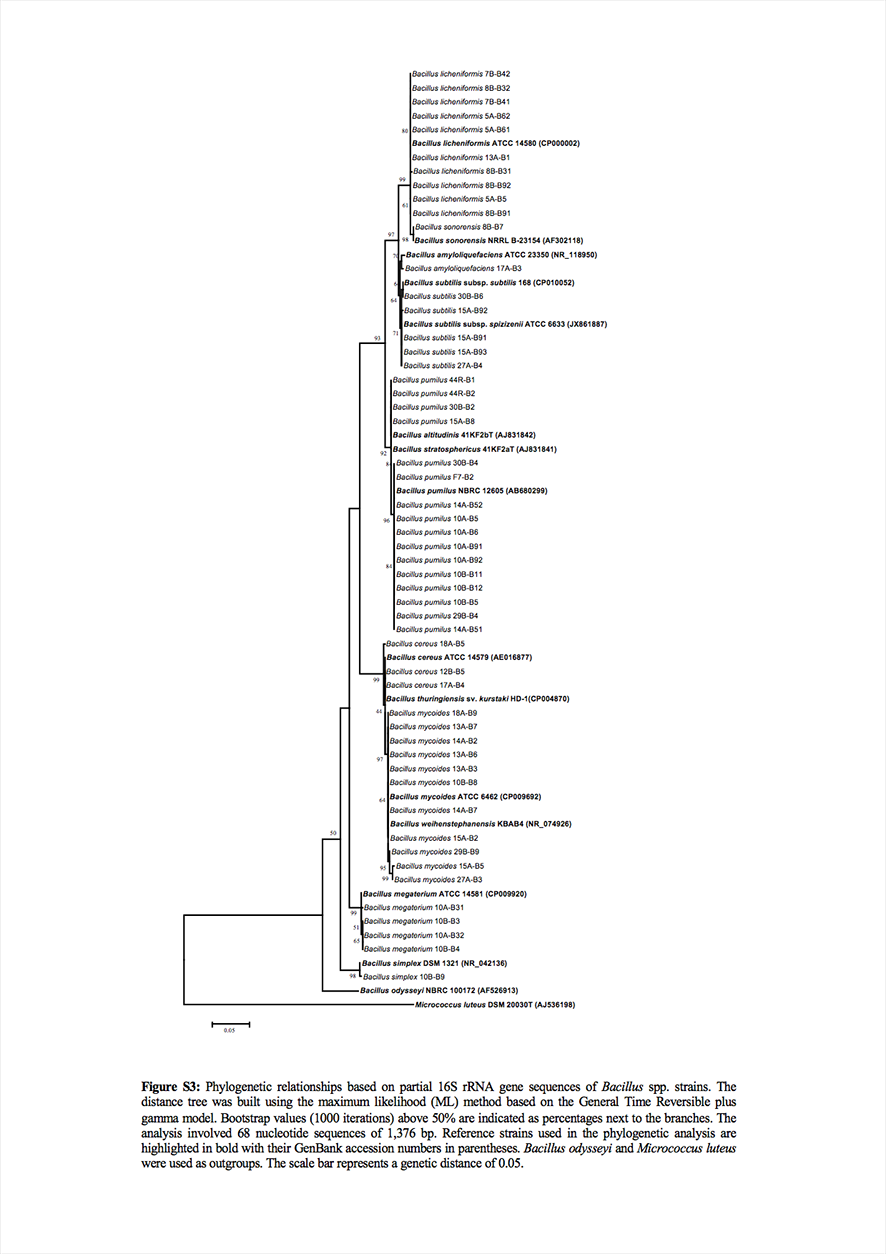

Supplement: Supplementary file 4 [file Image3.TIFF]

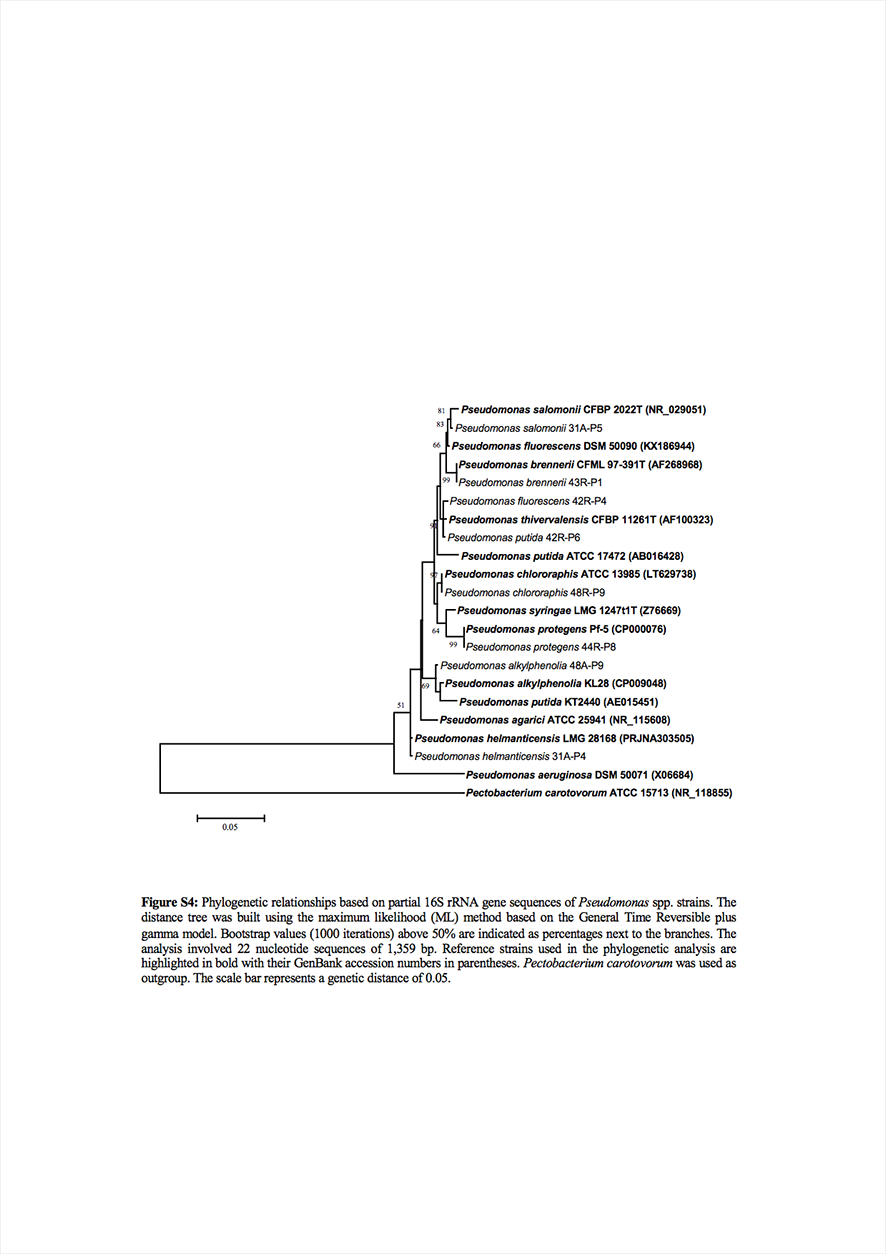

Supplement: Supplementary file 5 [file Image4.TIFF]

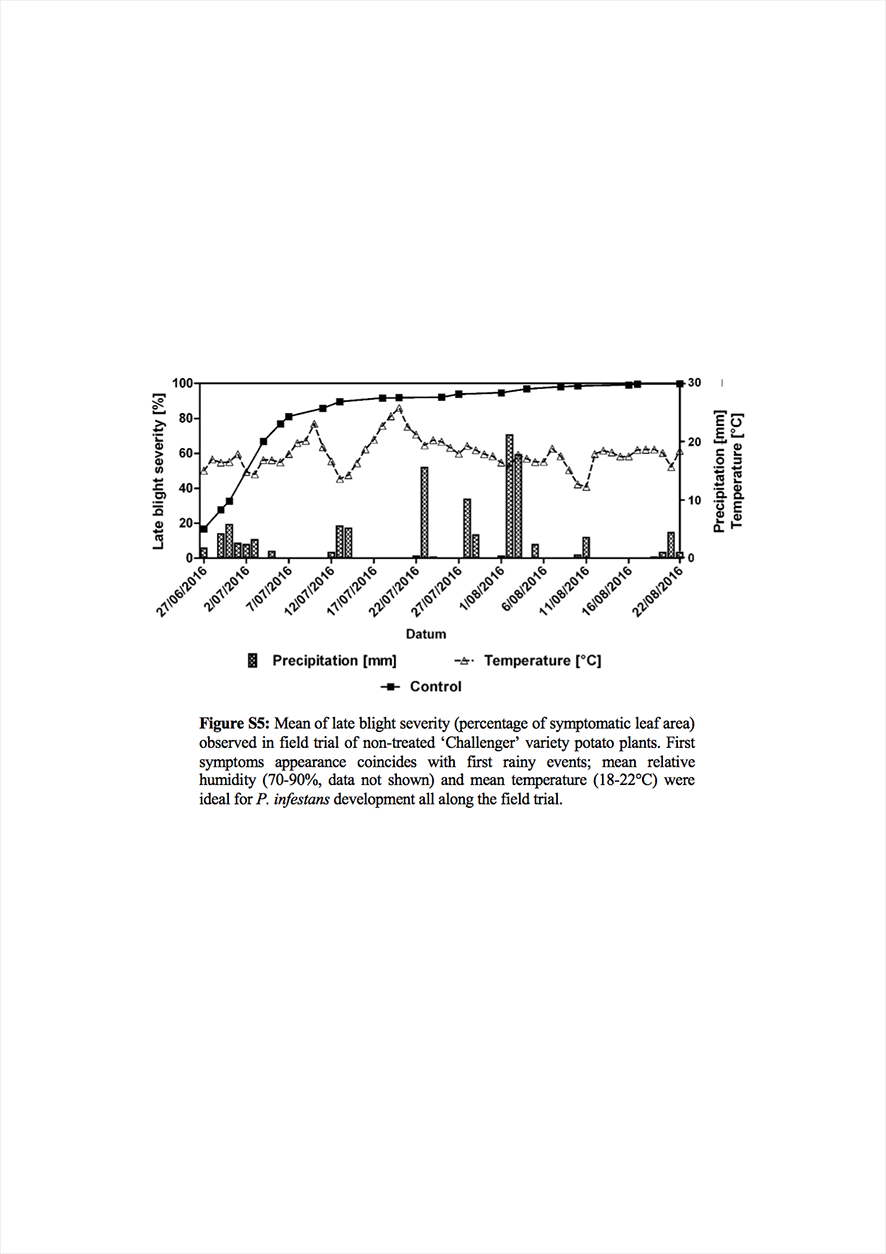

Supplement: Supplementary file 6 [file Image5.TIFF]
